# Synthesis and Characterization of Late Transition Metal Complexes of Mono-Acetate Pendant Armed Ethylene Cross-Bridged Tetraazamacrocycles with Promise as Oxidation Catalysts for Dye Bleaching

**DOI:** 10.3390/molecules28010232

**Published:** 2022-12-27

**Authors:** Tuyet Hoang, Somrita Mondal, Michael B. Allen, Leslie Garcia, Jeanette A. Krause, Allen G. Oliver, Timothy J. Prior, Timothy J. Hubin

**Affiliations:** 1 Department of Chemistry and Physics, Southwestern Oklahoma State University, Weatherford, OK 73096, USA; 2 Department of Chemistry, University of Cincinnati, Cincinnati, OH 45220, USA; 3 Department of Chemistry and Biochemistry, University of Notre Dame, Notre Dame, IN 46556, USA; 4 Department of Chemistry, University of Hull, Kingston Upon Hull HU6 7RX, UK

**Keywords:** oxidation catalyst, dye bleaching, cross-bridged tetraazamacrocycle, transition metal complex

## Abstract

Ethylene cross-bridged tetraazamacrocycles are known to produce kinetically stable transition metal complexes that can act as robust oxidation catalysts under harsh aqueous conditions. We have synthesized ligand analogs with single acetate pendant arms that act as pentadentate ligands to Mn, Fe, Co, Ni, Cu, and Zn. These complexes have been synthesized and characterized, including the structural characterization of four Co and Cu complexes. Cyclic voltammetry demonstrates that multiple oxidation states are stabilized by these rigid, bicyclic ligands. Yet, redox potentials of the metal complexes are modified compared to the “parent” ligands due to the pendant acetate arm. Similarly, gains in kinetic stability under harsh acidic conditions, compared to parent complexes without the pendant acetate arm, were demonstrated by a half-life seven times longer for the cyclam copper complex. Due to the reversible, high oxidation states available for the Mn and Fe complexes, the Mn and Fe complexes were examined as catalysts for the bleaching of three commonly used pollutant model dyes (methylene blue, methyl orange, and Rhodamine B) in water with hydrogen peroxide as oxidant. The efficient bleaching of these dyes was observed.

## 1. Introduction

Organic dyes have been widely used for industrial and commercial purposes, e.g., textiles, foods, pharmaceuticals, and cosmetics, as well as in scientific research [1,2,3,4]. However, many organic dyes are potentially toxic, carcinogenic, and often non-biodegradable. At high concentrations, strongly-absorbing dyes can reduce the light that is transmitted through natural bodies of water. Therefore, wastewater-containing organic dyes pose a potential threat to the aquatic environment as well as living organisms, including, ultimately, humans. Consequently, the elimination of organic dyes from wastewater is an environmental, water purification, and reuse necessity.

To address this environmental and water reuse concern, dye-bleaching has been broadly explored over the past decade [5,6,7]. “Advanced Oxidation” processes have been developed in which transition metal ions, particularly iron-aqua complexes, can efficiently oxidize “contaminants of emerging concern” (CEC), such as organic dyes, by using strong oxidants that then result in radical or “Fenton” mechanisms [8]. Unfortunately, these types of catalysts are only stable within a narrow, acid pH range that may not be applicable in large-scale water purification and reuse applications. Additionally, consideration of solid-state heterogeneous oxidation catalysts is needed that could be removed by filtration and recycled or used in a cartridge for large-scale water purification.

Other conventional methods include photocatalysis involving semiconductor metal oxides and sulfides [9,10,11,12]. However, their practical application is limited due to their large bandgap, which essentially restricts their usage, in most cases, to UV radiation. Among the metal oxides and sulfides, nanosized TiO_2_ is the most efficient for dye-bleaching [13]. However, in practice, nanosized TiO_2_ is limited due to its low surface area, high cost, and limited absorption ability [14].

Transition metal complexes having cross-bridged macrocyclic ligands have been extensively studied both in academia and industry. The role of cross-bridged tetraazamacrocycle transition metal complexes is well established as potent and mechanistically flexible oxidation catalysts [15,16,17,18,19,20,21,22,23,24,25,26,27,28,29,30,31]. Ligand topological constraint and rigidity factors render these catalysts extremely kinetically stable under harsh conditions where most transition metal complexes would lose their ligand(s) and/or form metal oxide/hydroxide decomposition products, rendering them useless [32]. Due to their kinetic stability, ethylene cross-bridged tetraazamacrocycle transition metal complexes have also been investigated in other applications, such as biomedical imaging [33,34,35,36,37,38,39] and anti-cancer therapy [40,41,42,43], where long-term stability is an asset. Nevertheless, their application is yet to be explored in dye-bleaching studies.

The original ethylene cross-bridged ligand catalysts were Mn and Fe complexes dimethylated at the non-cross-bridged nitrogen atoms referred to as the Busch catalysts, a patented bleach catalyst for activating peroxide or oxygen in water [44,45,46,47]. The Hubin group has produced additional analogs of these original catalysts with a range of pendant arms added to modify the electronic and steric properties, and thus the reactivity, of the catalysts produced [48,49,50].

**Figure 1:**
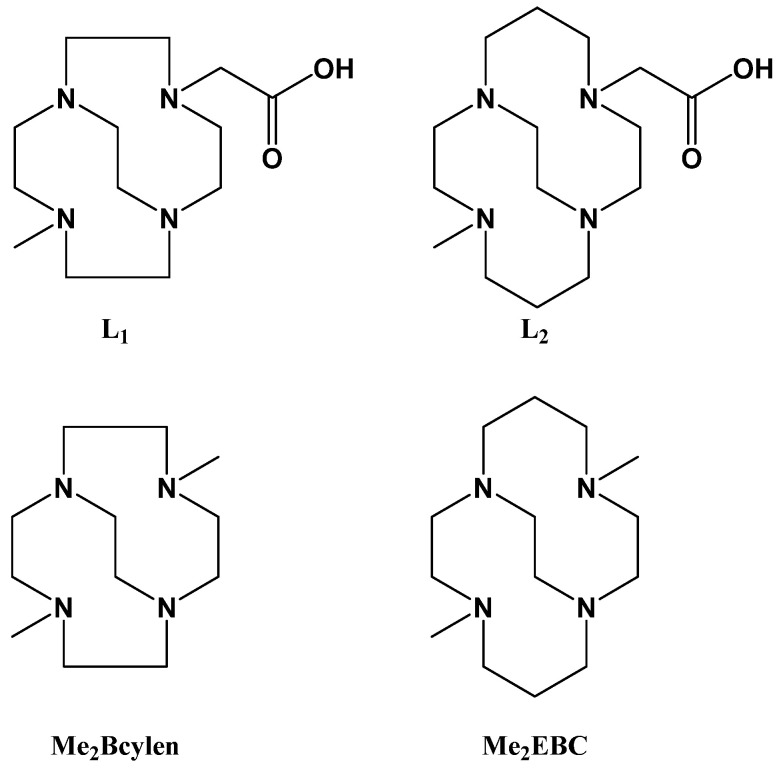
Ligands **L_1_** and **L_2_**, inspired by the **Me_2_Bcyclen** and **Me_2_EBC** analogs.

Herein, we report a facile synthesis of (OAc)MeBCyclen (**L_1_**) and (OAc)MeBCyclam (**L_2_**) ligands with their respective Mn, Fe, Co, Ni, Cu, and Zn complexes (Figure 1). The synthetic route is cost-effective and highly efficient, with appreciable synthetic yields, leading to pure crystalline products. The ligands and complexes were characterized by ^1^H and ^13^C NMR, LCMS, elemental analysis, UV-vis, and X-ray crystallography. The complexes are shown to be robust, as demonstrated by the kinetic stability of the Cu complex in highly acidic conditions at elevated temperatures.

Finally, we have explored the role of these cross-bridged metal complexes as oxidation catalysts, specifically for the bleaching of organic dyes, namely, methylene blue (MB), methyl orange (MO), and Rhodamine-B (Rhd-B). It was observed that the Mn and Fe complexes exhibited significant activity in bleaching MB, MO, and Rhd-B in ~1 mM concentration of the dyes. Thus, in practice, these complexes can be useful for cost-effective dye-bleaching, which can significantly overcome the drawback of conventional dye-bleaching photocatalysts. Moreover, this study can further pave the way for the design of a series of stable, cost-effective materials for purifying wastewater from organic pollutants.

## 2. Results and Discussion

### 2.1. Ligand Synthesis and Metal Complexation

One new ligand, L_1,_ and one previously published ligand, L_2_ [51], were prepared in good yield and purity from known ethylene cross-bridged precursors. Debenzylation of A_1_, monomethyl-monobenzyl-cross-bridged cyclen (BnMeBcyclen) [43] produced B_1_, HMeBcyclen, a new compound according to a SciFinder^n^© search. The analogous reaction starting from A_2_ (BnMeBcyclam) [41] yielded the known B_2_ analog [51,52]. Both B_1_ and B_2_ were prepared with good yield (88–93%) and purity. Iodoethyl acetate was used to modify both B_1_ and B_2_ according to typical conditions for the alkylation of secondary amines in cross-bridged tetraazamacrocycles [53]. The resulting new C_1_ and known C_2_ [51] ethyl esters were then hydrolyzed using a strongly basic ion exchange resin [51], resulting in the final ligands L_1_ and L_2_, respectively.

**L_1_** and **L_2_** were targeted as they would provide pentadentate analogs of the well-known tetradentate **Me_2_Bcyclen** and **Me_2_EBC** ethylene cross-bridged ligands (Figure 1). They would leave an open coordination site (assuming six-coordinate geometry as possible for all first-row transition metals) while providing a predicted gain in complex stability due to the acetate pendant arm. This allows for tuning of the electronic properties of the metal complexes with the negatively charged acetate pendant arm bound to the metal and potentially stabilizing higher oxidation states for catalysis.

Complexation with first-row transition metal dichloride salts, Mn through Zn, was carried out using typical procedures for cross-bridged tetraazamacrocyles that included dry, aprotic solvents under inert atmosphere conditions to limit the oxidation of air-sensitive metals (Fe, Mn) and prevent protonation of the ligands. The complexations were successful and yielded satisfactory amounts of the 12 complexes in high purity, which were then used for further characterization and catalytic reactivity studies.

### 2.2. X-ray Crystallography

Single crystals of each of the samples were grown by the methods described above for all four copper and cobalt complexes and studied using routine laboratory X-ray diffraction methods. Table 1 lists selected bond lengths and angles.

**Figure 2:**
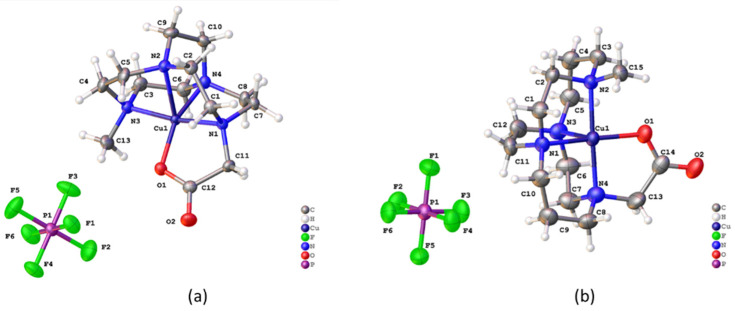
The asymmetric unit of (**a**) [Cu**L_1_**]PF_6_ and (**b**) [Cu**L_2_**]PF_6_ with atoms shown as 50% probability ellipsoids.

[Cu**L_1_**]PF_6_ (Figure 2A, Tables S8–S14) features five-coordinate Cu^2+^ in a distorted square pyramidal arrangement (τ_5_ = 0.223) [54]. All four nitrogen atoms and the carboxylate of the ligand coordinate to the metal with distances in the 1.9522(11)–2.189(13 Å range. Cu1 lies very slightly (0.14 Å) above the N1-N3-N4 mean plane. N2 is situated below this plane and rather distant from the metal (Cu1-N2 = 2.1891(13) Å), and the N-Cu-N angles involving N2 are 86.20(5), 85.38(5), and 80.73(5)°. The pendant carboxylate group coordinates with Cu1 and is strictly monodentate but is situated above the N1-N3-N4 plane such that the N2-Cu1-O1 angle is 119.60(5)°. In the solid state, the complexes are arranged in stacks parallel to the crystallographic b-axis, and there are C-H∙∙∙O interactions between adjacent complexes. The PF_6_^−^ anions are located between these stacks; there are C-H∙∙∙F interactions between the metal complexes and these anions.

The basic coordination of the copper in [Cu**L_2_**]PF_6_ (Figure 2B, Tables S22–S28) is similar to that in [Cu**L_1_**]PF_6_; the copper is five-coordinate with approximate square pyramidal coordination geometry (τ_5_ = 0.119). The copper-nitrogen and copper-oxygen distances fall in the 1.941(3)–2.219(3) Å range. The greater bite angle of the six-membered chelate rings means the pendant acetate is nearer to coplanarity with the plane of the three nitrogen atoms. The copper lies very slightly above (0.043 Å) the N2-N3-N4 plane, but the N1-Cu1-O1 angle is 170.34(13)°. In the solid state, the packing is similar to [Cu**L_2_**]PF_6_. There are stacks of metal complexes that run parallel to the b-axis, and between these are found C-H∙∙∙O interactions. Between the stacks are the PF_6_^−^ anions, and these form C-H∙∙∙F interactions with the complexes.

**Figure 3:**
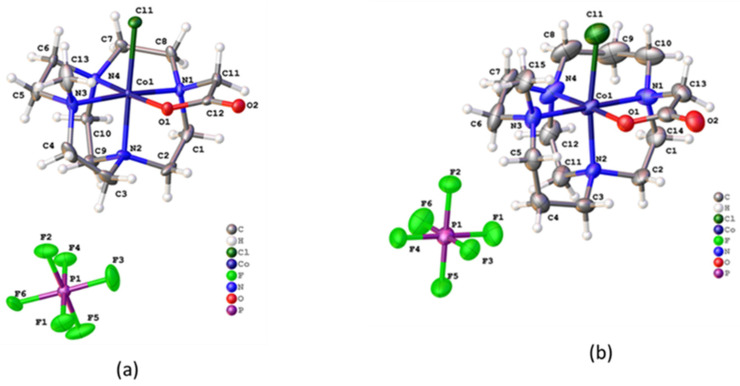
The asymmetric unit of (**a**) [Co**L_1_**Cl]PF_6_ and (**b**) [Co**L_2_**Cl]PF_6_ with atoms shown as 50% probability ellipsoids.

[Co**L_1_**Cl]PF_6_ (Figure 3A, Tables S1–S7) crystallizes in the centric space group P2_1_/n with a single octahedral cobalt in the asymmetric unit that is coordinated by five donor atoms from the ligand and a single chloride. The Co-Cl bond is substantially longer (2.2854(7) Å) than the other bonds about cobalt (1.922(2)–1.939(2) Å) with Co1-N3 as the exception (1.997(3) Å). In general, cis M-L bonds are very close to 90°; however, each of the N-Co-N angles in [Co**L_1_**Cl]^+^ is less than 90° (lie in the range 84.87(12) to 89.26(10)°). The r.m.s. deviation at the metal center for all cis angles from 90° is 3.55°. As observed for the Cu complexes, in the solid state, the Co complex also adopts a stacked motif that runs parallel to the a-axis, and between these are found C-H∙∙∙O interactions. Between the stacks are the PF_6_^−^ anions, and these form C-H∙∙∙F interactions with the complexes.

[Co**L_2_**Cl]PF_6_ (Figure 3B, Tables S15–S21) crystallizes in space group P2_1_/c of the monoclinic crystal system. The molecular structure is comparable to [Co**L_1_**Cl]PF_6_ and features an octahedral cobalt surrounded by five coordinating atoms of the macrocyclic ligand (Co-N,O = 1.901(3)-2.035(4) Å) and a single chloride, Co1-Cl1 = 2.3014(7) Å. N-Co-N angles for cis bonds are 93.48(17) and 93.90(13)° for the six-membered chelate rings and lie in the range 85.55(12)–89.00(18)° for the 5-membered chelates. The r.m.s. deviation from 90° for all cis ligands is 3.12°. The use of the program OctaDist [55] to analyze octahedral geometry distortions about a metal ion highlights the long Co-Cl bond but otherwise suggests the distortion observed is small, and there is no substantial difference between the coordination of cobalt in the two complexes [55]. In the solid state, there are stacks of metal complexes that run parallel to the a-axis, and between these are found C-H∙∙∙O interactions. Between the stacks are stacks of PF_6_^−^ anions, and these form C-H∙∙∙F interactions with the complexes.

### 2.3. Cyclic Voltammetry

**Figure 4:**
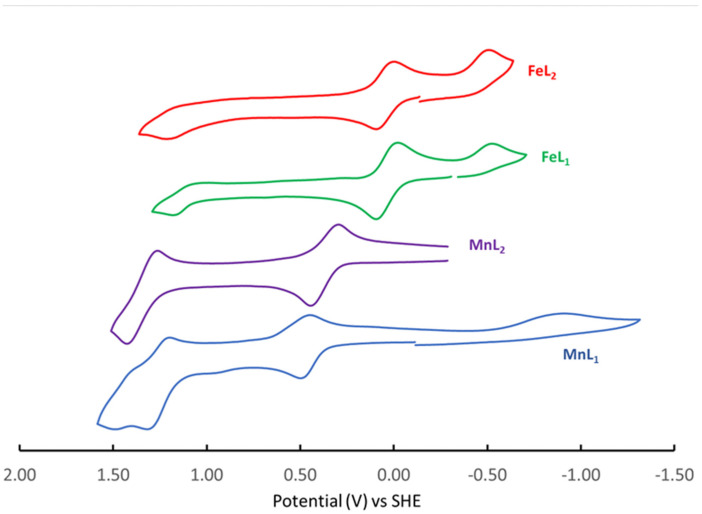
Cyclic voltammograms in acetonitrile with potentials relative to SHE for Mn**L_1_** (blue), Mn**L_2_** (purple), **FeL_1_** (green), and Fe**L_2_** (red).

Cyclic voltammetry on 0.001 M samples with TBAPF_6_ as a supporting electrolyte was obtained to determine redox potentials that might suggest or rationalize the use of the complexes as potential oxidation catalysts. Potentials are given versus SHE and were determined using ferrocene (+0.400 V vs. SHE) or acetylferrocene (+0.680 V vs. SHE) as an internal reference. Table 2 contains the redox potentials for processes associated with the redox active metal complexes (Zn^2+^ complexes were not examined). Figures S9–S18 contain the cyclic voltammograms for all examined complexes. Figure 4 contains the cyclic voltammograms for the iron and manganese complexes, which are the most relevant to the dye-bleaching examined in this work.

Interestingly, the cobalt complexes of **L_1_** and **L_2_** lose access to some irreversible Co^3+^/^4+^ oxidations and irreversible Co^2+^/^1+^ reductions that **Me_2_Bcyclen** and **Me_2_EBC** allow. Instead, the **L_1_** and **L_2_** complexes are limited to only one reversible Co^3+^/^2+^ redox couple, suggesting that the Co^3+^ and Co^2+^ ions are both stabilized by the pendant-armed ligands. Both the **L_1_** and **L_2_** couples are approximately 200 mV more negative than in the non-pendant armed ligands, showing that this reduction is more difficult to achieve with the acetate oxygen donor rather than the chloride. The oxygen donor is perhaps a better electronic match for the hard Co^3+^ ion, disfavoring reduction compared with having a softer chloride ligand in its place.

The nickel complexes of **L_1_** and **L_2_** exhibit similar behavior changes compared with their **Me_2_Bcyclen** and **Me_2_EBC** counterparts, as did the cobalt complexes, with loss of access to Ni^4+^ and Ni^+^, at least in the case of **L_2_**. In the **L_1_** nickel complex, a quasi-reversible Ni^3+/4+^ couple is observed. Perhaps the smaller size of the cyclen-based **L_1_** stabilizes the small Ni^4+^ ion, whereas the larger cyclam-based **L_2_** cannot. The non-pendant armed Ni(**Me_2_Bcyclen**)Cl_2_ and Ni(**Me_2_EBC**)Cl_2_ likely do not have reversibility for Ni^4+^ and Ni^+^ because the chloride ligands are not maintained at these extreme oxidation states, whereas, at least for **L_1_**, the complex does not change greatly (although this couple is only *quasi-reversible*) going to Ni^4+^. [Ni(**L_2_**)Cl]^+^ is significantly more difficult to oxidize to Ni^3+^ (+1.030 V) than is [Ni(**L_1_**)Cl]^+^ (+0.895 V), presumably reflecting the better fit of Ni^2+^ for the large cyclam-based ligand and the better fit of Ni^3+^ for the smaller cyclen-based ligand.

All copper complexes in Table 3 are five-coordinate, based on X-ray crystallography, with the **L_1_** and **L_2_** complexes’ fifth donor being acetate oxygen. At the same time, that site is a monodentate chloride in the **Me_2_Bcyclen** and **Me_2_EBC** complexes. [Cu(**Me_2_Bcyclen**)Cl]^+^ exhibits only an irreversible reduction to Cu^+^, with likely loss of the Cl^−^ ligand. [Cu(**Me_2_EBC**)Cl]^+^ exhibits a reversible Cu^2+/1+^ couple, as the larger cyclam ligand appears to stabilize the larger Cu^+^ cation. Both copper complexes of **L_1_** and **L_2_** exhibit reversible reductions to Cu^+^, perhaps illustrating the overall stability imparted by the pendant arm as compared to the non-pendant armed ligands. Interestingly, [Cu(**L_2_**)Cl]^+^ is ~150 mV more difficult to reduce than [Cu(**L_1_**)Cl]^+^ (−1.025 V to −0.846 V, respectively), which is the opposite trend of the non-pendant armed ligand complexes where the larger cyclam ligand accommodates the larger Cu^+^ more easily. Perhaps [Cu(**L_2_**)Cl]^+^ is particularly suited for Cu^2+^, as the kinetic stability data below indicates.

The manganese and iron complexes were chosen to conduct dye-bleaching oxidation studies, as their literature analogs are well-known oxidation catalysts [15,16,17,18,19,20,21,22,23,24,25,26,27,28,29,30,31]. Figure 4 shows the cyclic voltammograms for the catalytically active **L_1_** and **L_2_** complexes. All four manganese complexes in Table 2 exhibit reversible Mn^2+/3+^ and Mn^3+/4+^ redox couples, which have been used to explain the utility of this class of compounds in catalytic oxidation. We designed ligands **L_1_** and **L_2_** to potentially alter the electrochemistry of the manganese and iron complexes, as well as to add to the kinetic stability of the catalysts by adding a pendant donor arm. It appears we have not appreciably altered the oxidation potentials, as the two cyclen-based complexes (**L_1_** and **Me_2_Bcyclen**) have nearly identical redox potentials and reversibility. However, Mn(**L_1_**)Cl has added irreversible oxidation, perhaps from speciation of the Mn^3+^ complex with a slightly different ligand set, at +1.451 V, only slightly higher than the Mn^3+/4+^ reversible couple at +1.235 V. These values are too close to indicate a higher Mn^5+^ species. Hence, speciation in solution is more likely. Mn(**L_2_**)Cl exhibits neither of these added irreversible signals and mimics the redox behavior of Mn(**Me_2_EBC**)Cl_2_, the most well-known catalyst of this class. Mn(**L_2_**)Cl is ~150 mV easier to oxidize to Mn^3+^ than is Mn(**Me_2_EBC**)Cl_2_, but the oxidation to Mn^4+^ is nearly identical for these two complexes, suggesting a similar oxidizing power. As will be discussed in the section below, both Mn(**L_1_**)Cl and Mn(**L_2_**)Cl are efficient dye-bleaching catalysts, as might be expected of complexes with this access to higher oxidation states in a reversible fashion.

Finally, the iron complexes of **L_1_** and **L_2_** both exhibit the typical reversible Fe^2+/3+^ redox couple for complexes with cross-bridged tetraazamacrocyclic ligands, like Fe(**Me_2_Bcyclen**)Cl_2_ and Fe(**Me_2_EBC**)Cl_2_. Fe(**L_1_**)Cl has this couple at nearly the identical potential as does Fe(**Me_2_Bcyclen**)Cl_2_, indicating little effect of the acetate arm in place of a chloride ligand. Similarly, Fe(**L_2_**)Cl and Fe(**Me_2_EBC**)Cl_2_ differ by only 0.063 V, again indicating little difference between the electronic effects of the pendant acetate donor and a chloride. A major difference between the **L_1_** and **L_2_** complexes and that of their non-pendant armed analogs is the appearance of both an irreversible oxidation to Fe^4+^ and an irreversible reduction to Fe^+^. Neither of these signals is reported for either Fe(**Me_2_Bcyclen**)Cl_2_ or Fe(**Me_2_EBC**)Cl_2_. Because they are irreversible, the complex produced after each redox process is likely unstable. However, it is revealing that they occur at all. The pendant acetate arm appears to facilitate both processes within the potential window of acetonitrile for both Fe complexes, whereas the non-pendant armed ligands do not.

### 2.4. Kinetic Stability

Acid decomplexation studies of copper complexes revealed their stability in aqueous media [58,59]. Weisman et al. have established the temperature and acid environment conditions for studying the stability of cross-bridged copper complexes in a standard systematic way [18,48,52,54,60]. By examining the kinetic stability of the copper complex of a new ethylene cross-bridge ligand, one can discern the effect of ligand alteration(s) on the ability of the copper complex to survive harsh conditions. It is expected that the relative difference between the stabilities of the copper complexes will translate to other metal complexes of the examined ethylene cross-bridged ligands. Herein, we have calculated the half-lives following pseudo-first-order kinetics of the copper metal complexes in the presence of acid. Table 3 contains the half-life values for the copper complexes at different temperatures and in different acid environments.

**Table 3:** Half-life values for selected copper complexes in an acid environment.

Ligand	Pseudo-First Order Half-Life for Decomplexation at Various Conditions						
	30 °C, 1 M HCl	30 °C, 1 M HClO_4_	40 °C, 1 M HClO_4_	50 °C 5M HCl	70 °C, 5M HCl	90 °C, 5M HCl	References
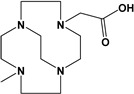 **L_1_**	9 min	33.94 h	16.0 h	<1 min			This work
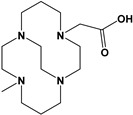 **L_2_**				18.5 days	6.95 days	9.01 h	This work
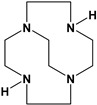 **H_2_Bcyclen**	<1 min						[31]
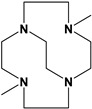 **Me_2_Bcyclen**	36 min		30 h	<1 min			[57]
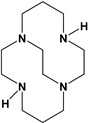 **H_2_Bcyclam**						11.8 min	[60]
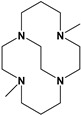 **Me_2_EBC**			>6 years	7.3 day		79 min	[17,57]
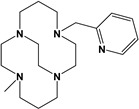 **Py_1_Me_1_Bcyclam**				14.7 min		<2 min	[17]
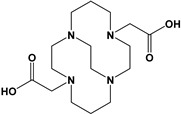 **CB-TE2A**						154 h	[60]
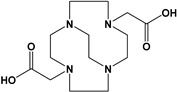 **CB-DO2A**	4 h						[34,60]
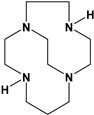 **H_2_B13N4**	4.8 h						[18]
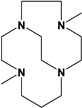 **Me_2_B13N4**	7.7 day			30.1 min		<2 min	[31]
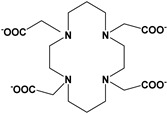 **TETA**				3.2 h		4.5 min	[60]

Comparison of the half-lives of cyclen-based complexes suggests that the unsubstituted cyclen (**H_2_Bcyclen**) complex decomposes very quickly, even at 30 °C in 1 M HCl solution [31]. Substitution with two methyl groups improves the stability significantly; the **Me_2_Bcyclen** complex has a half-life of 30 h at 40 °C in 1 M HClO_4_ solution. However, it was not very stable in 1 M HCl solution with a half-life of just 36 min [57]. Substitution with two acetate groups augments the stability significantly; **CB-DO2A** has a half-life of 4 h even at 30 °C in 1 M HCl solution [34,60]. In the present case, substitution with one methyl group and one acetate group of **L_1_** gave rise to a half-life of only 9 min at 30 °C in 1 M HCl solution. Since the [Cu**L_1_**] complex was not very stable in HCl, we examined its behavior in HClO_4_. [Cu**L_1_**] exhibited a significantly longer half-life of 33.94 h at 30 °C in 1 M HClO_4_ solution, which demonstrates that H^+^ alone in the acid solutions is not solely responsible for the decomplexation rate and that Cl^−^ must play some role. The influence of Cl^−^ on the kinetic stability of cross-bridged tetraazamacrocycles is well-known and may be attributed to the metal binding ability of Cl^−^, considered a coordinating ligand, compared to ClO_4_^−^, considered a non-coordinating ligand [31,61]. We repeated the decomplexation in HClO_4_ at 40 °C for direct comparison to Cu(**Me_2_Bcyclen**)Cl^+^. At this temperature, [Cu**L_1_**] had a half-life of only 16.0 h as compared to the 30 h half-life of Cu(**Me_2_Bcyclen**)Cl^+^ at 40 °C in 1 M HClO_4_ solution. We expected [Cu**L_1_**] to be longer lived than the Cu(**Me_2_Bcyclen**)Cl^+^ due to the pendant acetate replacing a non-binding methyl group, but we observed the opposite. The reasoning behind this result is not well understood at this point but could be the result of a mismatch in geometry and topology between the metal and ligand in [Cu**L_1_**] [17]. One may speculate that the acetate pendant arm may assist in the decomplexation by serving as a shuttle for H^+^ to move from the solution to the ligand. A similar phenomenon was observed for **PyMeBcyclam** [17], which destabilized its copper complex (half-life less than 2 min at 90 °C in 5 M HCl) vs. the **Me_2_EBC** ligand (half-life of 79 min at 90 °C in 5 M HCl). Clearly, the presence of a pendant arm as the fifth donor is not sufficient to guarantee increased stability compared to the tetradentate “parent” ligand.

Comparing the half-lives of the cyclam-based complexes indicates that the **H_2_Bcyclam** complex decomposed the fastest (half-life of 11.8 min at 90 °C in 5 M HCl solution) [60]. It would seem that unsubstituted **H_2_Bcyclam** has a huge stability advantage over **H_2_Bcyclen** even at higher temperatures and acid concentrations. This is likely due to a size and geometry match of the larger cyclam ring with Cu^2+^. Substitution with two methyl groups, as in the **Me_2_EBC** complex, increased the half-life to 79 min at 90 °C and 7.3 days at 50 °C in 5 M HCl solution. The **Me_2_EBC** complex was extraordinarily stable at 40 °C in 1 M HClO_4_ solution with a half-life of >6 years [17]. Substituting the methyl groups with two acetate groups leads to remarkable stability; the **CB-TE2A** copper complex has a half-life of 154 h even at 90 °C in 5M HCl solution [60]. In this work, we substituted **H_2_Bcyclam** with one methyl and one acetate group, generating **L_2_**. Interestingly, the [Cu**L_2_**] complex exhibited half-lives between the **Me_2_EBC** and **CB-TE2A** complexes (half-life of 18.5 days at 50 °C, 6.95 days at 70 °C, and 9 h at 90 °C in 5M HCl solution), as one might predict.

A recent paper by Iglesias et al. [62] details experiments on the dissociation and stability of copper complexes with many of the ligands in Table 3, as well as others. Their goal was to understand the mechanisms leading to dissociation in biological systems. Hence conditions studied are not directly comparable to those in Table 3, which were meant to demonstrate ligand structure vs. kinetic stability under harsh conditions.

### 2.5. Dye Degradation Studies

Since our goal is to treat wastewater through the effective oxidation of organic contaminants (contaminants of emerging concern, CEC), we have chosen three organic dyes, namely, methylene blue (MB), methyl orange (MO), and Rhodamine B (RhB) as model substrates for our oxidation catalyst—the Mn and Fe complexes of **L_1_** and **L_2_**. They are widely used dyes in several industries, e.g., foods, textiles, paper, cosmetics, rubber, pharmaceutics, plastics, and leather, as well as scientific research [63]. Methylene Blue (MB) and Rhodamine B (RhB) are cationic dyes, whereas methyl orange is an anionic dye. Another reason to choose these dyes is the presence of diverse functional groups, namely = NMe_2_^+^ in MB, -NMe_2_^+^ and -SO_3_^−^ groups in MO, -COOH group, and = NEt_2_ ^+^ groups in RhB.

Intriguingly, our metal complex oxidation catalysts were able to degrade all three dyes regardless of the functional group. At pH = 7, changes in dye absorbance were monitored by UV-vis spectroscopy at different time intervals in the absence and presence of metal catalysts. Control experiments were performed using hydrogen peroxide and metal chloride salts. Conversion % and turnover frequency (TOF) values for hydrogen peroxide were very low in the absence of the metal catalysts. In studies with only FeCl_2_, the TOF values were low for MB and RhB decolorization but high for MO degradation. In comparison, TOF values for the degradation of all three dyes using MnCl_2_ were lower than for the Mn-containing catalysts.

**Figure 5:**
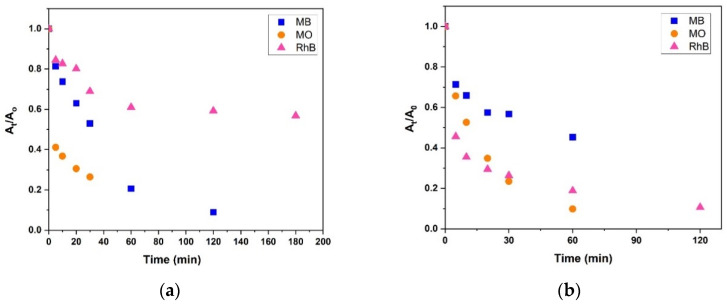
Plot of A_t_/A_0_ vs. time for decolorization of 3 dyes (MB, MO, and RhB) using(**a**) [Mn**L_1_**Cl] and (**b**) [Fe**L_1_**Cl] as catalysts. [Note: The time = 0 initial points for all three dyes are congruent, as the time = 0 point is defined as 1.0 in the ratio A*_t_*/A_0_ for all three dyes. Individual plots of each dye are available in the supplementary information.].

Graphing A_t_/A_0_ vs. time for [Mn**L_1_**Cl] (Figure 5a) shows that the degradation of MO was the fastest and the degradation of RhB was the slowest. Moreover, when using [Mn**L_1_**Cl], the TOF for MB and MO de-coloration was satisfactory but extremely low for RhB (Table 4). Table 4 shows that conversion % for MB (91%) and MO (77%) were high for this catalyst but low for RhB (37%).

Figure 5b shows that the bleaching of MO was the fastest and MB was the slowest using the [Fe**L_1_**Cl] catalyst. TOF for the degradation of MB was satisfactory, TOF for MO was relatively high, and TOF for RhB was low (Table 4). Comparing the TOF values of the Fe and Mn complexes, [Fe**L_1_**Cl] is more efficient (based on TOF) than the Mn complex for the degradation of all three dyes. However, the conversion % for this catalyst was lower for MB (49%) than for its Mn analog, while the conversion % for MO (89%) and RhB (90%) were increased.

**Figure 6:**
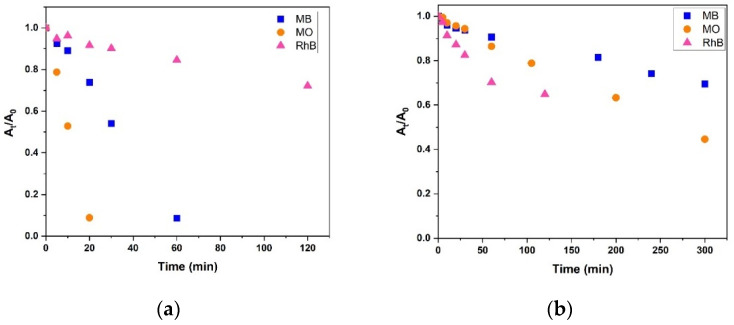
Plot of A_t_/A_0_ vs. time for decolorization of 3 dyes (MB, MO, and RhB) using (**a**) [Mn**L_2_**Cl] and (**b**) [Fe**L_2_**Cl] as catalysts. [Note: The time = 0 initial points for all three dyes are congruent, as the time = 0 point is defined as 1.0 in the ratio A_t_/A_0_ for all three dyes. Individual plots of each dye are available in the supplementary information.].

Figure 6 displays the A_t_/A_0_ vs. time graph for the degradation of the dyes using [Mn**L_2_**Cl] and [Fe**L_2_**Cl] catalysts. Similarly, [Mn**L_2_**Cl] showed the fastest degradation for MO and the slowest for RhB; whereas the dye degradation rate was comparable for all three dyes using [Fe**L_2_**Cl]. The TOF values were low for all three dyes using [Fe**L_2_**Cl] (Table 4). TOF values for the [Fe**L_2_**Cl] were even lower than those when the FeCl_2_ catalyst was employed. Conversion % was also low for MB (20%) and MO (17%), although better (68%) for RhB, which seems more susceptible to our iron catalysts.

In contrast, [Mn**L_2_**Cl] exhibited satisfactory catalytic efficiency for the degradation of MB (Figure 6a). TOF for degradation of MO using Mn-cyclam complex was extremely high, with the MO dye being almost completely decomposed within 30 min. However, TOF values for the degradation of RhB were low for [Mn**L_2_**Cl] (Table 4). Conversion % for the three dyes were MB (100%), MO (93%), and RhB (37%). It appears that the different metal ions have different selectivity for the dyes, with Mn more selective for MB and MO, with Fe more selective for RhB. Perhaps a combination of both metal ion catalysts would be possible to provide the broadest dye bleaching possible.

Yin et al. reported the degradation of MB, MO, and RhB using Mn(**Me_2_EBC**)Cl_2_ as the catalyst [64]. However, in their work, the bleaching of MO and RhB was very fast, while the bleaching of MB was slow. In addition, they studied the MB decolorization at different pHs and found the MB degradation was fast at pH 1.3 and 12.7 and moderate at pH 1.9, 3.1, 9.4, and 11.0. However, MB decolorization using Mn(**Me_2_EBC**)Cl_2_ was very slow at neutral pH (6.7). In contrast, our catalysts exhibited satisfactory dye-bleaching rates at neutral pH. We calculated TOF values for the Yin Mn(**Me_2_EBC**)Cl_2_ catalyst for all three dyes at neutral pH and found MB was very low (TOF value 0.067) compared to our catalysts. TOF for MO was comparable to our case (TOF value 7.344), and RhB was high (TOF value 5.928) (Table 4). Therefore, our catalysts may be superior in practical applications of degradation of MB at neutral pH.

It was interesting to observe that [Fe**L_1_**Cl] exhibited better catalytic efficiency than [Mn**L_1_**Cl] for the degradation of all three dyes, whereas [Mn**L_2_**Cl] was clearly superior to [Fe**L_2_**Cl] overall. There is no clear trend in terms of a single metal (Mn or Fe) or ligand (**L_1_** or **L_2_**) for superior degradation of all three dyes. This work represents our entry into the field of dye bleaching and only contains preliminary data at one pH for this small set of catalysts. Though we have not gained a full understanding of the parameters governing metal/ligand/dye selection to optimize a given degradation reaction, we have gained preliminary data suggesting that ethylene cross-bridged transition metal complexes are worthy of continued development toward the degradation of CECs, which we plan to continue to pursue.

## 3. Experimental

### 3.1. General Procedures

Pd (5%) on activated carbon was purchased from Strem Chemical. Anhydrous metal salts (MCl_2_, M = Mn, Fe, Co, Ni, Cu, Zn), glyoxal (40% wt in water), methyl iodide (99%), iodoethyl acetate (98%) and sodium borohydride (98%) were purchased from Aldrich Chemical Co (St. Louis, MO, USA). All solvents were of reagent grade and were dried, when necessary, by accepted procedures. Elemental analyses were performed on a Perkin-Elmer EA2400 elemental analyzer (Perkin-Elmer, Waltham, MA, USA). Electrospray mass spectra were collected on a Shimadzu LCMS 2020 Electrospray Mass Spectrometer (Shimadzu, Kyoto, Japan). NMR spectra were obtained on a Varian Bruker AVANCE II 300 MHz NMR Spectrometer instrument (Varian Bruker, Billerica, MA, USA). Electronic spectra were recorded using a Shimadzu UV-240 UV-Vis Spectrometer (Shimadzu, Kyoto, Japan). Electrochemical experiments were performed on a BAS100B Electrochemical Analyzer (BASi, West Lafayette, IN, USA). A button Pt electrode was used as the working electrode with a Pt-wire counter electrode and an Ag-wire pseudo-reference electrode. Scans were taken at 200 mV/s. Acetonitrile solutions of the complexes (1 mM) with tetrabutylammonium hexafluorophosphate (0.1 M) as a supporting electrolyte were used. The measured potentials were referenced to SHE with ferrocene (+0.400 V vs. SHE) or acetylferrocene (+0.680 V vs. SHE) as an internal standard. All electrochemical measurements were carried out under N_2_.

### 3.2. Synthesis

**Scheme 1:**
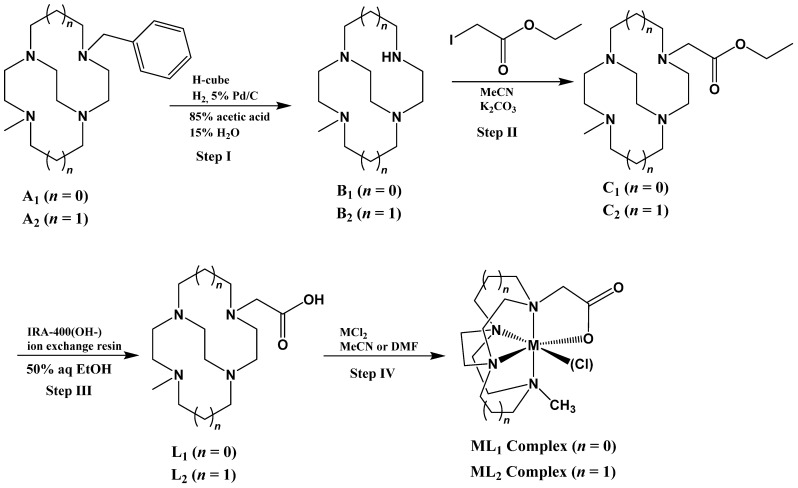
Combined synthetic scheme for the synthesis of L_1_, L_2_, and their metal complexes.

Precursors A (Scheme 1) have previously been published (**A_1_** [43], **A_2_** [41]) and served as the starting points for the syntheses of **L_1_** and **L_2_**.

#### 3.2.1. Synthesis of Ligands L_1_ and L_2_

Step I. Debenzylation.

**A_1_** (6.742 g, 22.3 mmol) was dissolved in 340 mL of 85% acetic acid (15% DI water) and filtered through celite (see Scheme 1). The clear solution was debenzylated in an H-cube Mini+ flow cell reactor using 5% Pd/C catalyst (~0.475 g of 50%Pd/C—50%H_2_O in a 70 mm CatCart), elevated H_2_ pressure, and elevated temperature in a continuous flow hydrogenation system (H-Cube Mini+, Thales Nano, Budapest, Hungary). The reaction parameters for the H-cube were 80 °C, 80 bar H_2_ pressure, and 1 mL/min flow rate. After evaporation of the product solution, the product was dissolved in 200 mL 30% KOH and extracted with benzene (5 × 100 mL). The organic layer was dried over anhydrous sodium sulfate overnight. After filtering out the solid sodium sulfate, the benzene solvent was removed using a rotary evaporator, and the collected white solid product was dried under vacuum, yielding 4.402 g (93%) of **B_1_**. Elemental analysis (%): calcd C_11_H_24_N_4_. 0.06 C_6_H_6_. 0.4 H_2_O (224.23 g·mol^−1^): C 60.85, H 11.31, N 24.99; Found C 61.24, H 11.65, N 25.33. MS (ES) *m*/*z* = 213 (LH^+^).

**A_2_** (6.230 g, 18.9 mmol) was debenzylated under identical conditions. The yield of the colorless oil was 3.989 g (88%) of **B_2_**. Elemental analysis (%): calcd C_13_H_28_N_4_. 0.3 H_2_O (245.79 g·mol^−1^): C 63.53, H 11.73, N 22.73; Found C 63.39, H 11.42, N 22.42. MS (ES) *m*/*z* = 241 (LH^+^).

Step II. Addition of Ethyl Acetate arm. **B_1_** (1.961 g, 9.24 mmol) was dissolved in 350 mL acetonitrile. 4 eq. potassium carbonate (5.105 g, 36.9 mmol) was added. 1 eq. ethyl iodoacetate (1.976 g, 1.093 mL, 9.24 mmol), and the reaction was stirred at room temperature overnight (see Scheme 1). The solid excess potassium carbonate was filtered out of the solution and washed with 50 mL acetonitrile. The combined acetonitrile fractions were evaporated and dried under vacuum, yielding 2.728 g (99%) of **C_1_** as a pale yellow oil. Elemental analysis (%): calcd C_15_H_30_N_4_O_2_. 0.2 H_2_O (312.84 g·mol^−1^): C 57.59, H 10.18, N 17.91; Found C 57.73, H 9.92, N 17.81. MS (ES) *m*/*z* = 299 (LH^+^).

**B_2_** (3.290 g, 13.7 mmol) was reacted under identical conditions. Yield **C_2_** as a pale yellow oil was 4.245 g (95%). Elemental analysis (%): calcd C_17_H_34_N_4_O_2_.1.2 H_2_O (348.10 g·mol^−1^): C 58.66, H 10.54, N 16.10; Found C 58.53, H 10.25, N 15.93. MS (ES) *m*/*z* = 327 (LH^+^).

Step III. Hydrolysis of Ethyl Acetate arm to Acid. An IRA-400(OH^−^) ion exchange resin was used to hydrolyze the ester. The resin was prepared by soaking and stirring 150 mL IRA-400(OH^−^) resin in 250 mL sodium hydroxide solution (2M) twice for 24 h each (see Scheme 1). This was followed by washing with 500 mL of deionized H_2_O. **C_1_** (2.728 g, 9.32 mmol) was then dissolved in 200 mL 50% ethanol/water in a 500 mL round bottom flask and stirred with 150 mL of the ion exchange resin overnight. The resin was removed by filtration, and the solvent was evaporated and dried under vacuum overnight, yielding **L_1_** (1.865 g, 74%) as a colorless oil. Elemental analysis (%): calcd C_13_H_26_N_4_O_2_. 0.4 C_2_H_6_O. 3 H_2_O (342.85 g·mol^−1^): C 48.35, H 10.11, N 16.34; Found C 48.44, H 9.71, N 16.55. MS (ES) *m*/*z* = 271 (LH^+^).

**C_2_** (4.245 g, 13.7 mmol) was reacted under identical conditions. The yield of **L_2_** as a colorless oil was 3.453 g (89%). Elemental analysis (%): calcd C_15_H_30_N_4_O_2_. 0.3 CHCl_3_. 3.8 H_2_O (390.76 g·mol^−1^): C 46.72, H 9.75, N 14.34; Found C 47.00, H 10.06, N 14.06. MS (ES) *m*/*z* = 299 (LH^+^).

#### 3.2.2. Step IV. Synthesis of Metal Complexes

**L_1_** (0.270 g, 0.001 mol) or **L_2_** (0.298 g, 0.001 mol) were loaded into a four-dram vial and pumped into an inert atmosphere glovebox (see Scheme 1). Each metal salt reaction consisted of adding one of the following salts MnCl_2_, FeCl_2_, CoCl_2_, NiCl_2_, CuCl_2_, and ZnCl_2_ (0.126 g to 0.136 g, 0.001 mol) to the vial. Acetonitrile (15 mL) was used for each reaction, except DMF was used in the case of NiCl_2_ due to poor solubility in acetonitrile. The reactions were then stirred overnight in the glovebox.

The reaction vials were removed from the glovebox, with the exception of the Mn and Fe complexes. The Mn and Fe complex solutions were filtered to remove any solids. The filtrates were allowed to evaporate in the glovebox to produce the pure Mn and Fe products. After removal from the glovebox, the other metal complexations were also filtered, and the filtrates evaporated to dryness. The residue was re-dissolved in a minimum amount of methanol and placed in a 100-mL round bottom flask. In a separate vial, 5 eq. ammonium hexafluorophosphate (0.815 g, 0.005 mol) was dissolved in a minimum amount of methanol. While constantly stirring, the NH_4_PF_6_ solution was added via filter pipet to the metal complex solutions. The round bottom flasks were kept in a freezer for a day to complete product precipitation. The metal complexes were then collected by filtration. The filtrate was washed with minimal cold methanol, followed by diethyl ether, before being dried under a vacuum. Mass spec and elemental analyses for the individual complexes are given below:

[Mn(**L_1_**)Cl]Cl·0.5 H_2_O: wine red powder. 38% (0.154 g) Elemental analysis (%): calcd Mn(C_13_H_26_N_4_O_2_)Cl_2_·0.5H_2_O (405.22 g·mol^−1^): C 39.41, H 6.61, N 14.14; Found C 38.39, H 7.05, N 13.78. MS (ES) *m*/*z* = 327 (LMn^+^).

[Fe(**L_1_**)Cl]Cl·0.5 H_2_O: dark brown powder. 35% (0.142 g) Elemental analysis (%): calcd [Fe(C_13_H_26_N_4_O_2_)Cl_2_]·0.5 H_2_O (406.13 g·mol^−1^): C 39.32, H 6.60, N 14.11; Found C 38.39, H 6.91, N 13.92. MS (ES) *m*/*z* = 357 (LFeCl^+^).

[Co(**L_1_**)Cl_2_](PF_6_)·0.5 H_2_O: pink powder. 30% (0.167 g) Elemental analysis (%): calcd

[Co(C_13_H_26_N_4_O_2_)Cl](PF_6_)·0.5 H_2_O: (554.18 g·mol^−1^): C 28.17, H 4.91, N 10.11; Found C 27.90, H 4.81, N 10.37. MS (ES) *m*/*z* = 363 (LCoCl^+^). X-ray-quality crystals were grown from the evaporation of an acetone solution.

[Ni(**L_1_-H**)Cl](PF_6_): brown powder. 30% (0.153 g) Elemental analysis (%): calcd

[Ni(C_13_H_25_N_4_O_2_)Cl](PF_6_)]: (509.48 g·mol^−1^): C 30.71, H 4.96, N 11.02; Found C 30.88, H 5.35, N 11.42. MS (ES) *m*/*z* = 327 (LNi^+^).

[Cu(**L_1_**)](PF_6_)·1.6H_2_O: violet powder. 32% (0.160 g) Elemental analysis (%): calcd

[Cu(L-H)(PF_6_)·1.6H_2_O]: (506.70 g·mol^−1^): C 30.82, H 5.61, N 11.06; Found C 30.64, H 5.14, N 11.33. MS (ES) *m*/*z* = 332 (LCu^+^). X-ray quality crystals were grown from ether diffusion into a methanol solution.

[Zn(**L_1_-H**)](PF_6_)·0.2 NH_4_PF_6_: white powder. 28% (0.142 g) Elemental analysis (%): calcd [Zn(C_13_H_26_N_4_O_2_)Cl](PF_6_)·0.2 NH_4_PF_6_: (512.31 g·mol^−1^); C 30.48, H 5.08, N 11.48; Found C 30.31, H 5.10, N 11.45. MS (ES) *m*/*z* = 333 (LZn^+^).

[Mn(**L_2_**)Cl]·0.85CH_3_CN·2H_2_O: light pink powder. 28% (0.131 g) Elemental analysis (%): calcd [Mn(C_15_H_31_N_4_O_2_)Cl]·0.85CH_3_CN·2H_2_O; (458.73 g mol^−1^); C 43.72, H 7.81, N 14.81; Found C 44.07, H 7.39, N 15.15. MS (ES) *m*/*z* = 352 (LMn^+^).

[Fe(**L_2_**)Cl_2_]: dark brown powder. 34% 0.145 g Elemental analysis (%): calcd

[Fe(C_15_H_30_N_4_O_2_)Cl_2_]; (425.18 g·mol^−1^); C 42.37, H 7.11, N 13.18; Found C 42.61, H 7.51, N 12.97. MS (ES) *m*/*z* = 385 (LFeCl^+^); *m*/*z* = 371 (LFeOH^+^).

[Co(**L_2_**)Cl](PF_6_): dark violet powder. 20% (0.108 g) Elemental analysis (%): calcd

[Co(C_15_H_30_N_4_O_2_)Cl](PF_6_): (537.77 g·mol^−1^); C 33.50, H 5.62, N 10.42; Found C 33.46, H 5.44, N 10.17. MS (ES) *m*/*z* = 391 (LCoCl^+^); *m*/*z* = 373 (LCoOH^+^). X-ray quality crystals were grown from ether diffusion into an acetonitrile solution.

[Ni(**L_2_**)Cl](PF_6_)·0.8H_2_O: pale pink powder. 32% (0.180 g) Elemental analysis (%): calcd [Ni(C_15_H_30_N_4_O_2_ Cl](PF_6_)·0.8H_2_O; (551.95 g·mol^−1^); C 32.64, H 5.77, N 10.63; Found C 32.55, H 5.42, N 10.34. MS (ES) *m*/*z* = 355 (LNi^+^).

[Cu(**L_2_-H**)](PF_6_)·0.2NH_4_PF_6_: pale blue powder. 26% (0.140 g) Elemental analysis (%): calcd Cu(C_15_H_30_N_4_O_2_)(PF_6_)·0.2NH_4_PF_6_; (538.53 g·mol^−1^); C 33.45, H 5.58, N 10.92; Found C 33.68, H 5.30, N 10.75. MS (ES) *m*/*z* = 360 (LCu^+^). X-ray quality crystals were grown from ether diffusion into a DMF solution.

[Zn(**L_2_**)Cl](PF_6_)·2H_2_O; white powder. 28% (0.162 g) Elemental analysis (%): calcd

[Zn(C_15_H_30_N_4_O_2_)Cl](PF_6_)·2H_2_O; (580.26 g·mol^−1^); C 32.56, H 5.65, N 10.13; Found C 32.25, H 5.43, N 10.40. MS (ES) *m*/*z* = 361 (LZn^+^).

### 3.3. Characterization

Acid Decomplexation Studies. This experiment has become a standard method for determining kinetic stability to compare new cross-bridged ligands. In order to determine their kinetic stability, the d-d absorption band (generally near 600 nm) of the copper complexes (1 mM) in highly concentrated acidic solutions (generally 5 M HCl) [65] was followed on a Shimadzu UV-3600 UV−vis−near-IR spectrophotometer at increasing temperatures until conditions were found that decomposed the complex in a matter of hours/days. For **L_1_**, decomplexation in 5 M HCl was very fast, even at 30 °C. Therefore, we used 1 M HCl at 30 °C and then 1 M HClO_4_ at 30 °C and 40 °C to obtain decomplexation rates comparable to those of related ligands reported in the literature. Chloride is known to assist acidic decomplexation, whereas perchlorate is not. Thus, both were used to provide as many literature comparisons as possible. The reaction rate pattern followed pseudo-first-order kinetics. Half-lives of the metal complexes were calculated from the slope of ln absorbance vs. time plots.

X-Ray Crystallography Studies. X-ray scattering data from crystals of the samples were collected using a dual source Bruker APEX-II diffractometer with a PHOTON-II detector. For three samples, the structures were determined using Mo Kα radiation. For a single twinned sample, data were collected using Cu Kα radiation. Data were scaled and merged, and a multi-scan absorption correction was applied. Structures were solved using dual-space methods in SHELXT. Structures were refined using SHELXL-2018 [66,67] implemented within Olex2 [68]. All non-hydrogen atoms were refined using anisotropic displacement parameters. Hydrogen atoms were placed at calculated positions using a riding model. For the structure of L_2_Cu-PF_6_, the diffraction images clearly showed the presence of two twin domains; the longer wavelength of the copper source was used to help separate the spots of these domains. Intensity from the two domains was integrated using standard methods, and the structure was refined using the HKLF5 formalism. Crystal data for the complexes are summarized below. CCDC-2182279-2182282 contains the supplementary crystallographic data for this paper. These data can be obtained free of charge from The Cambridge Crystallographic Data Centre via www.ccdc.cam.ac.uk/data_request/cif accessed on 26 December 2022.

**[CoL_1_Cl]PF_6_:** C_13_H_25_ClCoF_6_N_4_O_2_P (*M* = 508.72 g/mol): monoclinic, space group P2_1_/n (no. 14), *a* = 7.9000(2) Å, *b* = 19.3699(5) Å, *c* = 12.2722(3) Å, *β* = 98.2620(10)°, *V* = 1858.43(8) Å^3^, *Z* = 4, *T* = 126.98 K, μ(MoKα) = 1.230 mm^−1^, *Dcalc* = 1.818 g/cm^3^, 45,049 reflections measured (3.958° ≤ 2Θ ≤ 56.564°), 4620 unique (*R*_int_ = 0.0546, R_sigma_ = 0.0293) which were used in all calculations. The final *R*_1_ was 0.0500 (I > 2σ(I)), and *wR*_2_ was 0.0979 (all data).

**[CuL_1_]PF_6_:** C_13_H_25_CuF_6_N_4_O_2_P (*M* = 477.88 g/mol): triclinic, space group P-1 (no. 2), *a* = 8.2596(8) Å, *b* = 8.7606(9) Å, *c* = 13.0051(12) Å, *α* = 77.500(3)°, *β* = 81.329(3)°, *γ* = 87.627(3)°, *V* = 908.19(15) Å^3^, *Z* = 2, *T* = 119.99 K, μ(MoKα) = 1.367 mm^−1^, *Dcalc* = 1.748 g/cm^3^, 24,102 reflections measured (4.762° ≤ 2Θ ≤ 56.614°), 4507 unique (*R*_int_ = 0.0283, R_sigma_ = 0.0217) which were used in all calculations. The final R_1_ was 0.0238 (I > 2σ(I)), and *w*R_2_ was 0.0574 (all data).

**[CoL_2_Cl]PF_6_:** C_15_H_29_ClCoF_6_N_4_O_2_P (*M* = 536.77 g/mol): monoclinic, space group P2_1_/c (no. 14), *a* = 8.6543(8) Å, *b* = 12.5675(12) Å, *c* = 18.5750(18) Å, *β* = 92.778(2)°, *V* = 2017.9(3) Å^3^, *Z* = 4, *T* = 120.05 K, μ(MoKα) = 1.138 mm^−1^, *Dcalc* = 1.767 g/cm^3^, 41,924 reflections measured (3.914° ≤ 2Θ ≤ 54.454°), 4491 unique (*R*_int_ = 0.0507, R_sigma_ = 0.0317) which were used in all calculations. The final *R*_1_ was 0.0681 (I > 2σ(I)), and *wR*_2_ was 0.1793 (all data).

**[CuL_2_]PF_6_:** C_15_H_29_CuF_6_N_4_O_2_P (*M* = 505.93 g/mol): monoclinic, space group P2_1_/c (no. 14), *a* = 9.5256(3) Å, *b* = 8.0586(3) Å, *c* = 25.2288(8) Å, *β* = 99.776(2)°, *V* = 1908.52(11) Å^3^, *Z* = 4, *T* = 127.01 K, μ(CuKα) = 3.145 mm^−1^, *Dcalc* = 1.761 g/cm^3^, 3322 reflections measured (7.11° ≤ 2Θ ≤ 133.158°), 3322 unique (*R*_int_ = 0.0552, R_sigma_ = 0.0424) which were used in all calculations. The final *R*_1_ was 0.0417 (I > 2σ(I)), and *wR*_2_ was 0.1034 (all data).

### 3.4. Dye Bleaching Studies

All studies were performed in an aqueous solution at neutral pH following the procedure reported by Yin [64]. The concentrations of the stock solution of the three dyes, namely, methylene blue (MB), methylene orange (MO), and Rhodamine B (RhB), were 3.35 mM, 3.83 mM, and 2.60 mM, respectively. The concentration of the metal complex stock solutions was 1.00 mM. In each set of experiments, 10.00 mL of metal complex stock solution was added to 10.00 mL of dye stock solution. The pH was adjusted to 7 using 0.100 M HCl or 0.100 M NaOH. Finally, water was added to a final volume of 25 mL. The final concentrations of MB, MO, RhB, and the metal complex were 1.34 mM, 1.53 mM, 1.04 mM, and 0.40 mM, respectively. Typically, 0.25 mL 30% H_2_O_2_ was added to each solution to initiate dye decolorization. To study the extent of reaction, 1 mL aliquots were removed from the reaction mixture at each time point. Each aliquot was diluted to 100 mL aqueous solution, which quenched the reaction, and the absorbance spectra were recorded for each diluted aliquot. The absorbance spectra were recorded at 664 nm, 465 nm, and 553 nm for the reaction mixture containing MB, MO, and RhB, respectively. The time of H_2_O_2_ addition was assumed as time zero. The reaction was monitored at 5, 10, 20, 30, 60, 120, 180, 240, and 300 min (and could be extended if needed for slower reactions) following the addition of H_2_O_2_. In general, reactions were continued until the absorbance of the dye reached ~10% of its initial absorption, although this target was not always reached. All reactions were performed in duplicate with the average values reported. Note: the concentration of catalyst was selected to match the concentration used by Yin in the only previously published dye bleaching study with cross-bridged tetraazamacrocycle catalysts. The concentration of the catalyst is ~1/3 that of the dye, which is not economically feasible but matches the literature for comparison and provides an initial screening experiment to identify if dye bleaching is feasible.

## 4. Conclusions

New, late first-row transition metal complexes of two monoacetate pendant-armed cross-bridged tetraazamacrocycles were synthesized and characterized. X-ray crystal structures of the Cu and Co complexes demonstrated the ability of the pendant arm to bind the metal ion. In the case of the Cu complexes, the kinetic stability of the cyclam-based ligand complex was enhanced over the dimethyl analog. In contrast, surprisingly, the kinetic stability of the cyclen-based ligand complex was reduced—perhaps due to the poor complementarity of this smaller ligand. Cyclic voltammetry demonstrated reversible access to 3+ and 4+ oxidation states for the Mn and Fe complexes and suggested they should be active oxidation catalysts. Dye bleaching of three common CEC dyes confirmed the catalytic oxidation ability of these complexes.

## Figures and Tables

**Table 1:** Selected bond lengths (Å) and Angles (°).

**[CoL_1_Cl]PF_6_**
**Bond lengths:**
Co(1)-Cl(1) 2.2854(7) Co(1)-N(4) 1.922(2)
Co(1)-O(1) 1.924(2) Co(1)-N(2) 1.931(2)
Co(1)-N(1) 1.939(2) Co(1)-N(3) 1.997(3)
**Bond angles:**
O(1)-Co(1)-Cl(1) 87.89(7) N(4)-Co(1)-N(1) 86.22(10)
O(1)-Co(1)-N(1) 87.43(9) N(4)-Co(1)-N(2) 89.26(10)
O(1)-Co(1)-N(2) 90.35(10) N(4)-Co(1)-N(3) 88.52(10)
O(1)-Co(1)-N(3) 97.77(9) N(2)-Co(1)-Cl(1) 178.21(8)
N(1)-Co(1)-Cl(1) 91.60(7) N(2)-Co(1)-N(1) 88.64(11)
N(1)-Co(1)-N(3) 171.70(11) N(2)-Co(1)-N(3) 84.87(12)
N(4)-Co(1)-Cl(1) 92.52(7) N(3)-Co(1)-Cl(1) 95.05(8)
N(4)-Co(1)-O(1) 173.64(9) N(4)-Co(1)-N(1) 86.22(10)
O(1)-Co(1)-Cl(1) 87.89(7) N(4)-Co(1)-N(2) 89.26(10)
O(1)-Co(1)-N(1) 87.43(9)
**[CuL_1_]PF_6_**
**Bond lengths:**
Cu(1)-O(1) 1.9522(11) Cu(1)-N(1) 1.9640(12)
Cu(1)-N(3) 1.9876(13) Cu(1)-O(1) 1.9522(11)
Cu(1)-N(2) 2.1891(13)
**Bond angles:**
O(1)-Cu(1)-N(3) 100.26 (5) N(3)-Cu(1)-N(4) 88.88(5)
O(1)-Cu(1)-N(2) 119.60(5) N(1)-Cu(1)-N(3) 171.41(5)
O(1)-Cu(1)-N(1) 85.39(5) N(1)-Cu(1)-N(2) 86.20(5)
O(1)-Cu(1)-N(4) 158.06(5) N(1)-Cu(1)-N(4) 88.09(5)
N(3)-Cu(1)-N(2) 85.38(5) N(4)-Cu(1)-N(2) 80.73(5)
**[CoL_2_Cl]PF_6_**
**Bond lengths:**
Co(1)-O(1) 1.901(3) Co(1)-N(1) 1.986(4)
Co(1)-N(2) 2.002(4) Co(1)-N(4) 1.961(4)
Co(1)-N(3) 2.035(4) Co(1)-Cl(1) 2.3014(17)
**Bond angles:**
O(1)-Co(1)-N(2) 88.92(15) N(1)-Co(1)-N(2) 86.96(16)
O(1)-Co(1)-N(3) 90.85(15) N(1)-Co(1)-N(3) 178.04(17)
O(1)-Co(1)-N(1) 87.26(16) N(1)-Co(1)-Cl(1) 85.55(12)
O(1)-Co(1)-N(4) 177.55(18) N(4)-Co(1)-N(2) 89.00(18)
O(1)-Co(1)-Cl(1) 87.36(11) N(4)-Co(1)-N(3) 87.97(18)
N(2)-Co(1)-N(3) 93.48(17) N(4)-Co(1)-N(1) 93.94(19)
N(2)-Co(1)-Cl(1) 171.78(12) N(4)-Co(1)-Cl(1) 94.87(15)
N(3)-Co(1)-Cl(1) 93.90(13) N(1)-Co(1)-N(2) 86.96(16)
O(1)-Co(1)-N(2) 88.92(15)
O(1)-Co(1)-N(3) 90.85(15)
**[CuL_2_]PF_6_**
**Bond lengths:**
Cu(1)-O(1) 1.941(3) Cu(1)-N(3) 2.219(3)
Cu(1)-N(1) 2.028(3) Cu(1)-N(4) 2.057(3)
Cu(1)-N(2) 2.049(3) Cu(1)-O(1) 1.941(3)
**Bond angles:**
O(1)-Cu(1)-N(1) 170.34(13) N(1)-Cu(1)-N(3) 86.27(13
O(1)-Cu(1)-N(2) 91.23(12) N(1)-Cu(1)-N(4) 94.46(13)
O(1)-Cu(1)-N(3) 103.38(12) N(2)-Cu(1)-N(3) 93.48(12)
O(1)-Cu(1)-N(4) 86.65(12) N(2)-Cu(1)-N(4) 177.48(12)
N(1)-Cu(1)-N(2) 87.86(13) N(4)-Cu(1)-N(3) 85.69(13)

**Table 2:** Redox potentials vs. SHE for M**L_1_** and M**L_2_** complexes (0.001 M in acetonitrile). Reference compounds of the same metals with **Me_2_Bcyclen** and **Me_2_EBC** ligands (see Figure 1) are included for comparison.

Complex	M^1+/2+^ E_red_ [V]	M^1+/2+^ E_1/2_ [V] (ΔE [mV])	M^2+/3+^ E_1/2_ [V] (ΔE [mV])	M^3+/4+^ E_1/2_ [V] (ΔE [mV])	M^3+/4+^ E_ox_ [V]	Reference
Mn**(Me_2_Bcyclen**)Cl_2_	-----	-----	+0.466 (70)	+1.232 (102)	-----	[20]
Mn(**Me_2_EBC**)Cl_2_	-----	------	+0.585 (61)	+1.343 (65)	-----	[20]
Mn(**L_1_**)Cl	−0.913	-----	+0.463 (60)	+1.235 (116)	+1.451	This work
Mn(**L_2_**)Cl	-----	-----	+0.371 (87)	+1.328 (88)	-----	This work
Fe(**Me_2_Bcyclen**)Cl_2_	-----	-----	+0.036 (64)	-----	-----	[20]
Fe(**Me_2_EBC**)Cl_2_	-----	-----	+0.110 (63)	-----	-----	[20]
Fe(**L_1_**)Cl	−0.526	-----	+0.037 (117)	-----	+1.182	This work
Fe(**L_2_**)Cl	−0.502	-----	+0.047 (87)	-----	+1.210	This work
Co(**Me_2_Bcyclen**)Cl_2_	−2.202	-----	−0.157 (288)	-----	+0.983	[16]
Co(**Me_2_EBC**)Cl_2_	−2.198	-----	+0.173 (103)	-----	+1.051	[16]
[Co(**L_1_**)Cl^+^]	-----	-----	−0.386 (156)	-----	-----	This work
[Co(**L_2_**)Cl^+^]	-----	-----	−0.157 (130)	-----	-----	This work
Ni(**Me_2_Bcyclen**)Cl_2_	−2.036	-----	+0.863 (68)	-----	+1.450	[56]
Ni(**Me_2_EBC**)Cl_2_	−1.894	-----	+0.991 (154)	-----	+1.325	[56]
[Ni(**L_1_**)Cl^+^]	-----	-----	+0.895 (161)	+1.373 (194)	-----	This work
[Ni(**L_2_**)Cl^+^]	-----	-----	+1.030 (182)	-----	-----	This work
[Cu(**Me_2_Bcyclen**)Cl^+^]	−0.651	-----	-----	-----	-----	[57]
[Cu(**Me_2_EBC**)Cl^+^]	-----	−0.544 (97)	+1.530 (irreversible)	-----	-----	[57]
[Cu(**L_1_**)^+^]	* E_cat_ = −0.303	−0.846 (104)	-----	-----	-----	This work
[Cu(**L_2_**)^+^]	-----	−1.025 (81)	-----	-----	-----	This work

* This catalytic peak is likely not associated with the free complex in solution; more likely an electrode surface phenomenon.

**Table 4:** Turnover Frequency (TOF) values for control and all four metal-complex catalysts for degradation of MB, MO, and RhB at pH = 7.

Catalyst	Average Conversion% For MB	Average TOF for MB (h^−1^)	Average Conversion% For MO	Average TOF for MO (h^−1^)	Average Conversion% For RhB	Average TOF for RhB (h^−1^)
H_2_O_2_ only	3	0.018	2	0.034	2	0.002
FeCl_2_ only	3	0.111	97	0.926	9	0.055
MnCl_2_ only	3	0.003	27	0.518	2	0.047
[Mn**L_1_**Cl]	91	1.050	77	2.034	37	0.327
[Fe**L_1_**Cl]	49	1.289	89	2.568	90	0.626
[Mn**L_2_**Cl]	100	1.657	93	10.749	35	0.365
[Fe**L_2_**Cl]	20	0.208	17	0.376	68	0.459
Mn(**Me_2_EBC**)Cl_2_ [64]	10	0.067	96	7.344	95	5.928

## Data Availability

Most of the data presented in this study are available in the supplementary material. Additional data presented in this study are available on request from the corresponding author.

## Supplementary Materials

The following supporting information can be downloaded at: https://www.mdpi.com/article/10.3390/molecules28010232/s1, Figures S1–S8: NMR spectra for L1 and L2 and their precursors and Zn complexes; Figures S9–S18: cyclic voltammograms of Mn, Fe, Co, Ni, and Cu complexes of **L_1_** and **L_2_**; Figure S19: an example of the determination of kinetic stability for **CuL_1_** in 1 M HClO_4_ at 40 °C; Figures S20–S31: Dye Bleaching plots; Tables S1–S28: X-ray crystallography data for [Co**L_1_**Cl]PF_6_, [Cu**L_1_**]PF_6_, [Co**L_2_**Cl]PF_6_, and [Cu**L_2_**]PF_6_.

## Sample Availability

Samples of the compounds are not available.
