# Supplementary material for: Synthesis and Characterization of Late Transition Metal Complexes of Mono-Acetate Pendant Armed Ethylene Cross-Bridged Tetraazamacrocycles with Promise as Oxidation Catalysts for Dye Bleaching
Source: Molecules. 2022 Dec 27;28(1):232. doi: 10.3390/molecules28010232 (PMC9822179; doi:10.3390/molecules28010232)
Supplement: Supplementary file 1 [file molecules-28-00232-s001.zip › molecules-2060354-supplementary.pdf]

Synthesis and Characterization of Late Transition Metal Complexes of Mono-Acetate Pendant Armed Ethylene Cross-Bridged Tetraazamacrocycles with Promise as Oxidation Catalysts for Dye Bleaching

Tuyet Hoang [1], Somrita Mondal [1], Michael B. Allen [1], Leslie Garcia [1], Jeanette A. Krause [2], Allen G. Oliver [3], Timothy J. Prior [4], and Timothy J. Hubin [1,*]

Supplementary Information

Table of Contents **Page**

1. Figures S1-S8. ^{1}H and ^{13}C NMR Spectra of $\mathbf{L_1}$ and $\mathbf{L_2}$ ligands and precursors and Zn complexes. 2-9
2. Figures S9-S18. Cyclic Voltammograms of $\mathbf{L_1}$ and $\mathbf{L_2}$ Metal Complexes 10-14
3. Figure S19. Example: kinetics of dissociation plots at 40 °C in 1 M $HClO_4$ for [Cu$\mathbf{L_1}$] 15
4. Figures S20-S31. Plots of A_t/A_0 vs time for dye bleaching by Fe and Mn catalysts. 16 – 21
5. Tables S1-S7. X-Ray Crystallography for [Co$\mathbf{L_1}$Cl]PF_6 22-28
6. Tables S8-S14. X-Ray Crystallography for [Cu$\mathbf{L_1}$]PF_6 29-34
7. Tables S15-S21. X-Ray Crystallography for [Co$\mathbf{L_2}$Cl]PF_6 35-40
8. Tables S22-S28. X-Ray Crystallography for [Cu$\mathbf{L_2}$]PF_6 41-46

Figure S1. a) ^{1}H NMR (a) and ^{13}C NMR (b) spectra for $\mathbf{B_1}$.

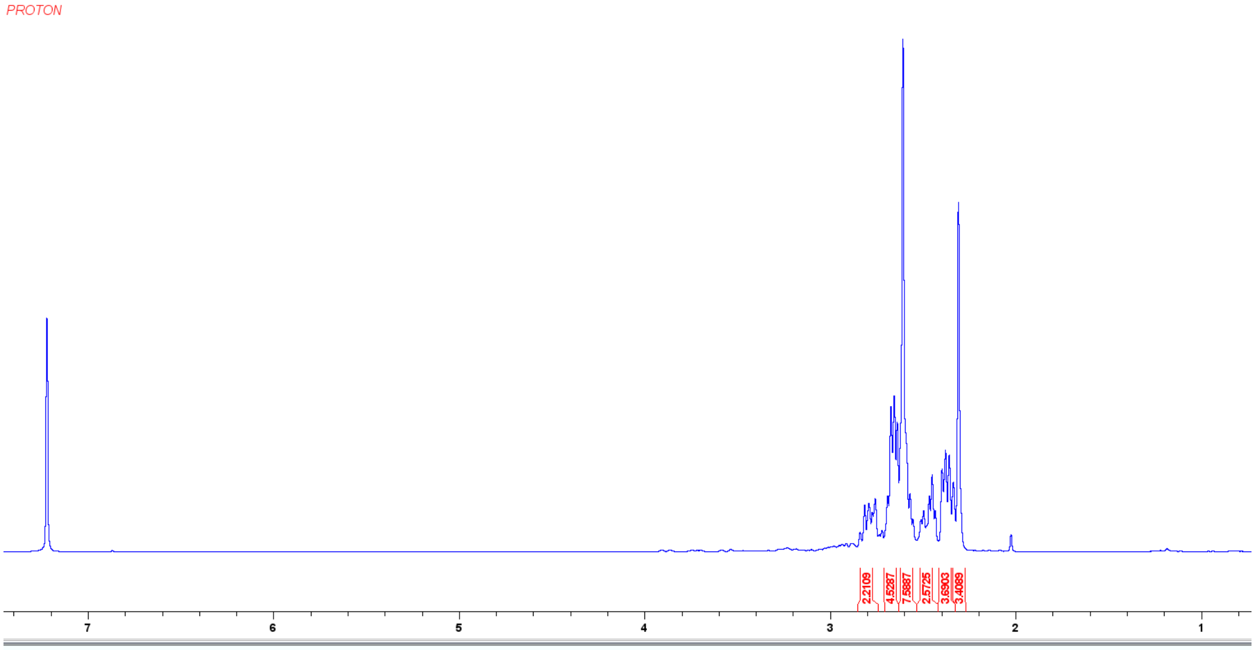

(a)

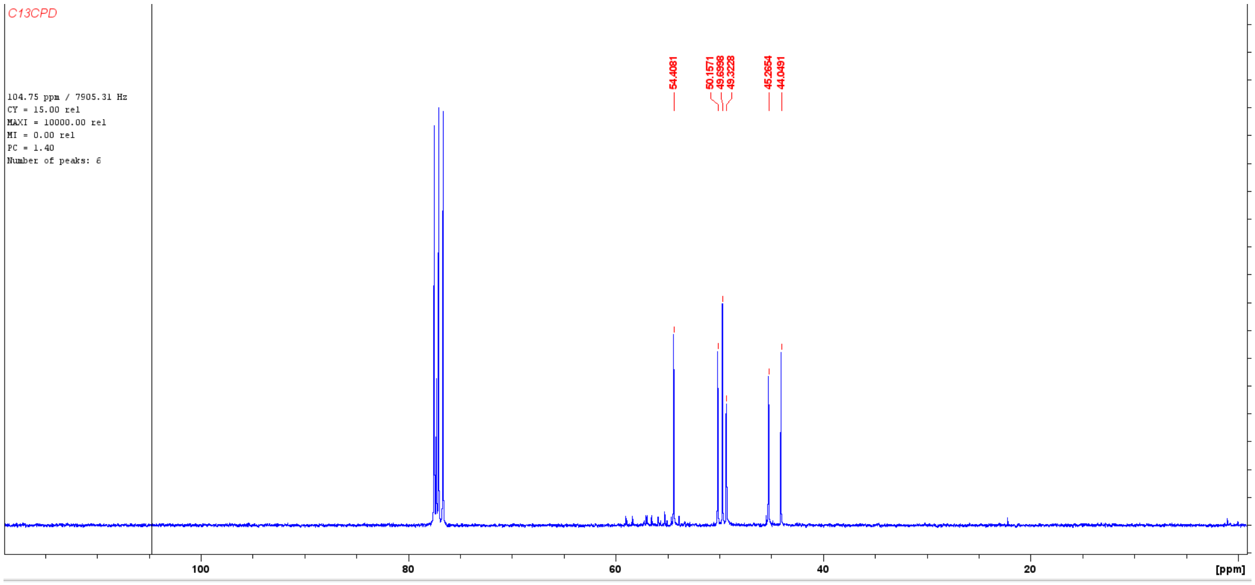

(b)

Figure S2. a) ^{1}H NMR (a) and ^{13}C NMR (b) spectra for **C_1**. 13C NOT CLEAN; ???

Tuyet never ran NMR's. TJH found TH123 which is the right compound and is taking NMR's right now

(a)

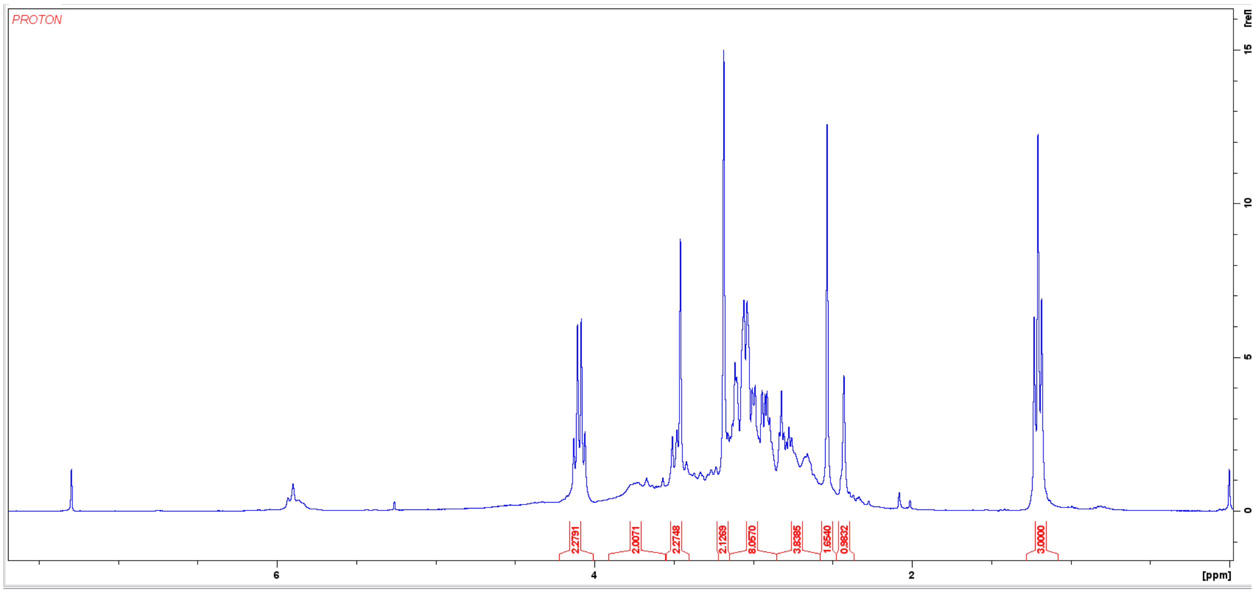

(b)

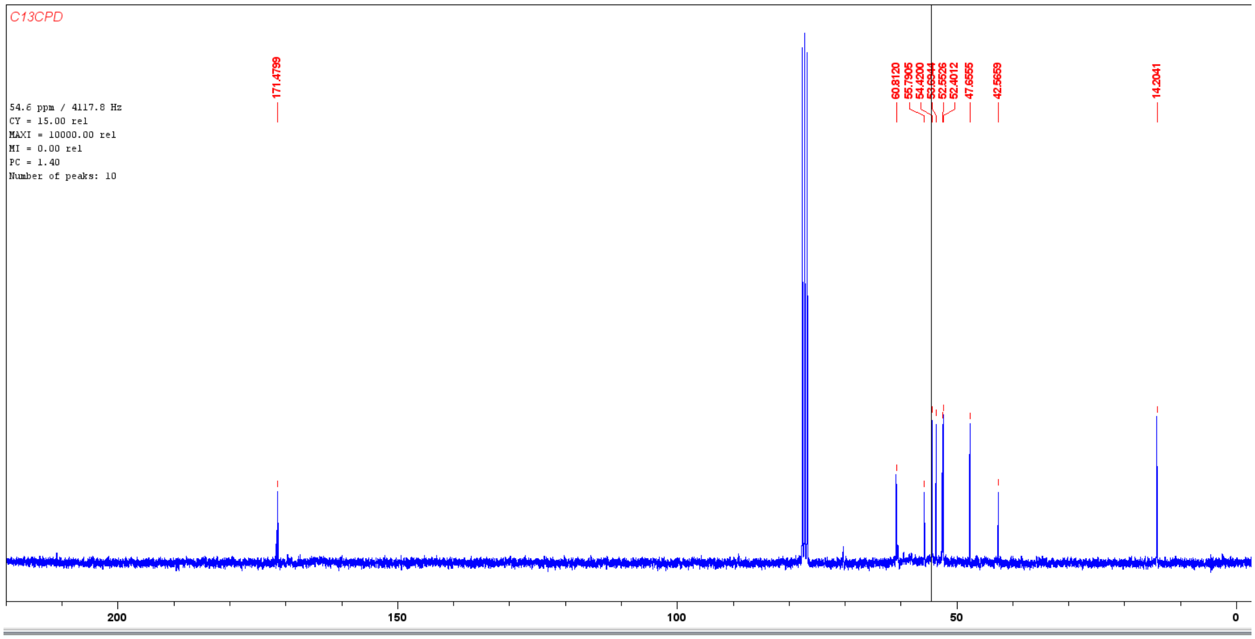

Figure S3. a) ^{1}H NMR (a) and ^{13}C NMR (b) spectra for **L$_1$**.

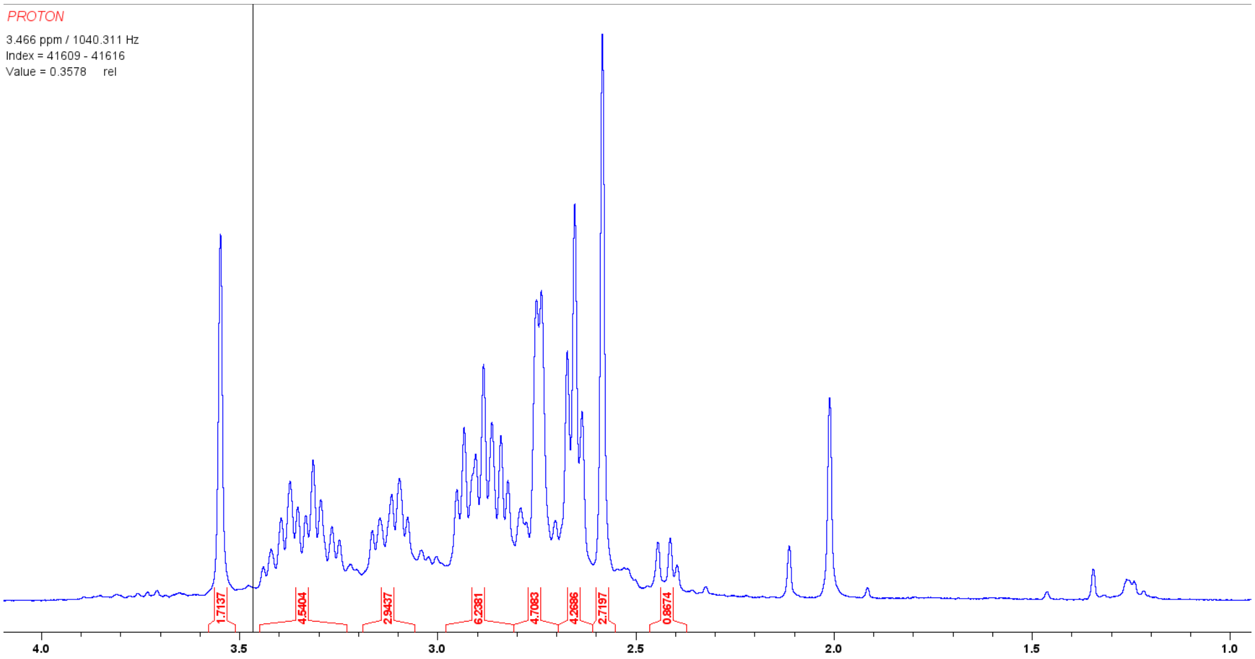

(a)

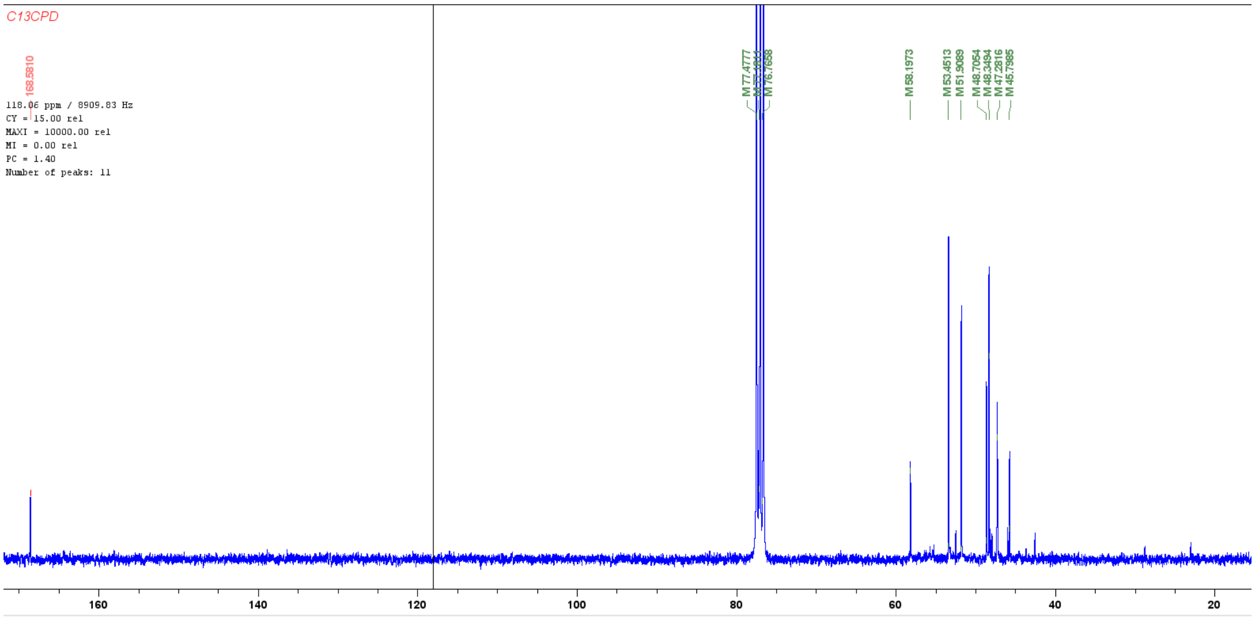

(b)

Figure S4. a) ^{1}H NMR (a) and ^{13}C NMR (b) spectra for **B₂**. This is a known compound [“MRI image enhancement compositions containing tetraazabicyclohexadecane manganese complexes” Perkins, Christopher Mark; Kitko, David Jonathan. World Intellectual Property Organization, WO2002026267 A2 2002-04-04], [“Copper(II) and zinc(II) complexation with N-ethylene hydroxycyclams and consequences on the macrocyclic backbone configuration” AlHaddad, Nancy; Lelong, Evan; Suh, Jong-Min; Cordier, Marie; Lim, Mi Hee; Royal, Guy; Platas-Iglesias, Carlos; Bernard, Helene; Tripier, Raphael Dalton Transactions (2022), 51(22), 8640-8656]

(a)

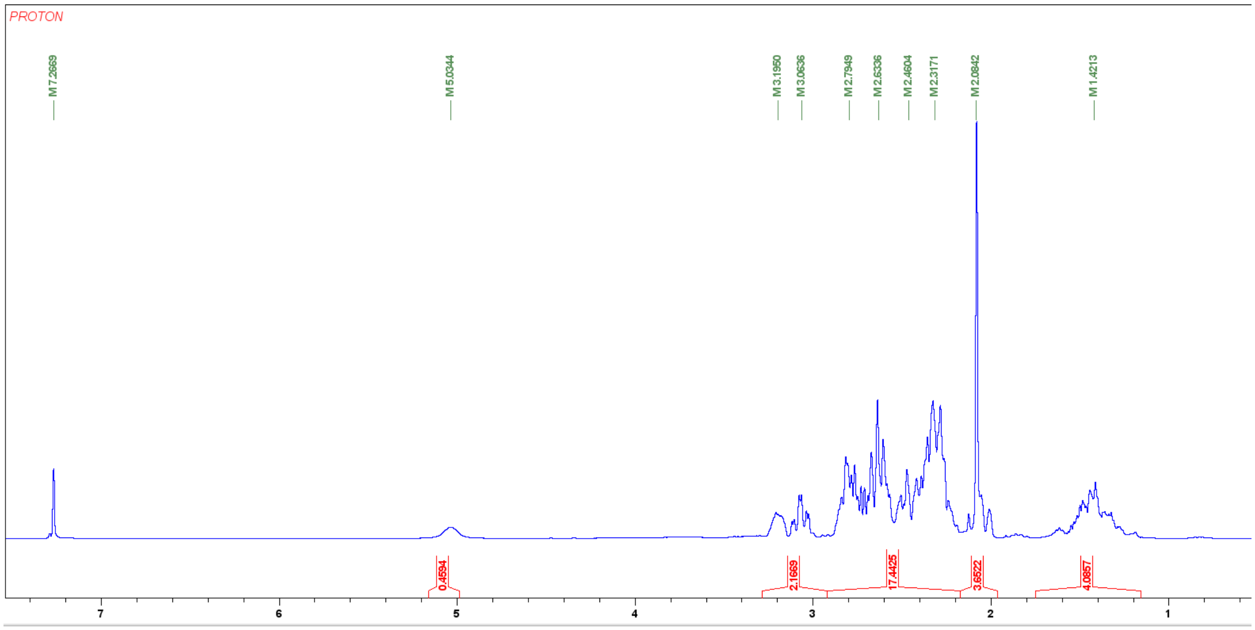

(b)

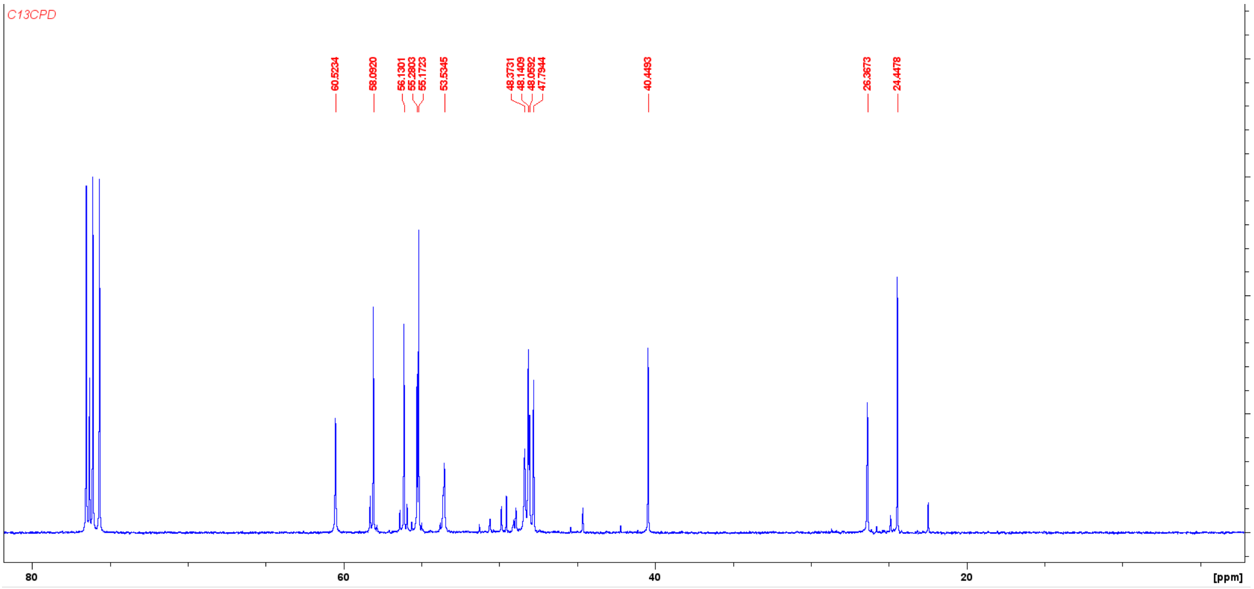

Figure S5. a) ^{1}H NMR (a) and ^{13}C NMR (b) spectra for $\mathbf{C_2}$.

(a)

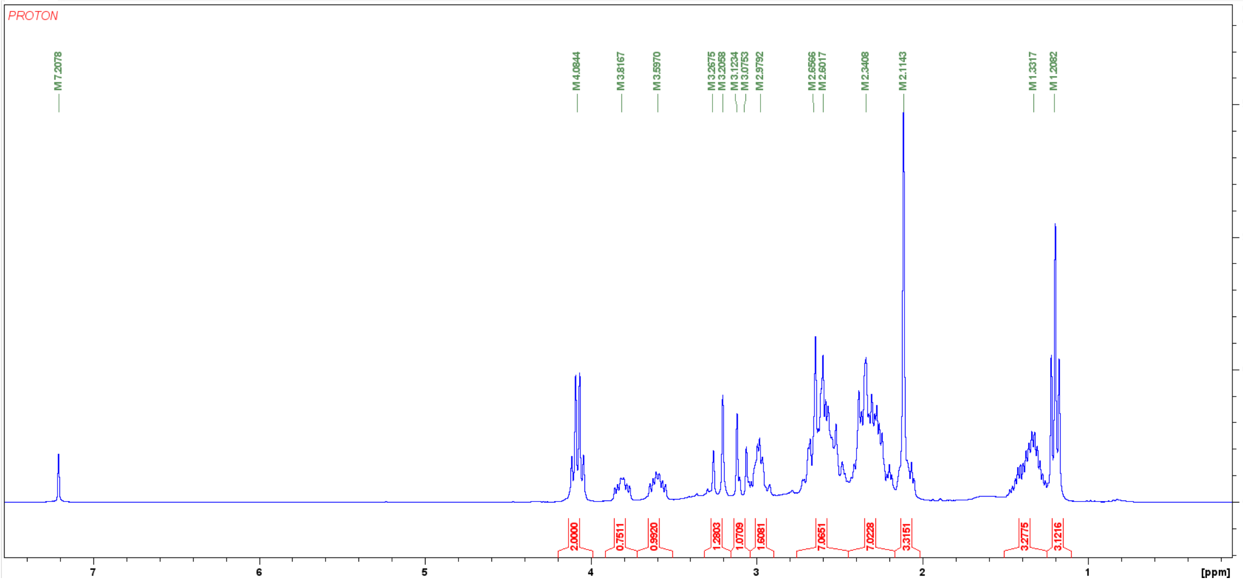

(b)

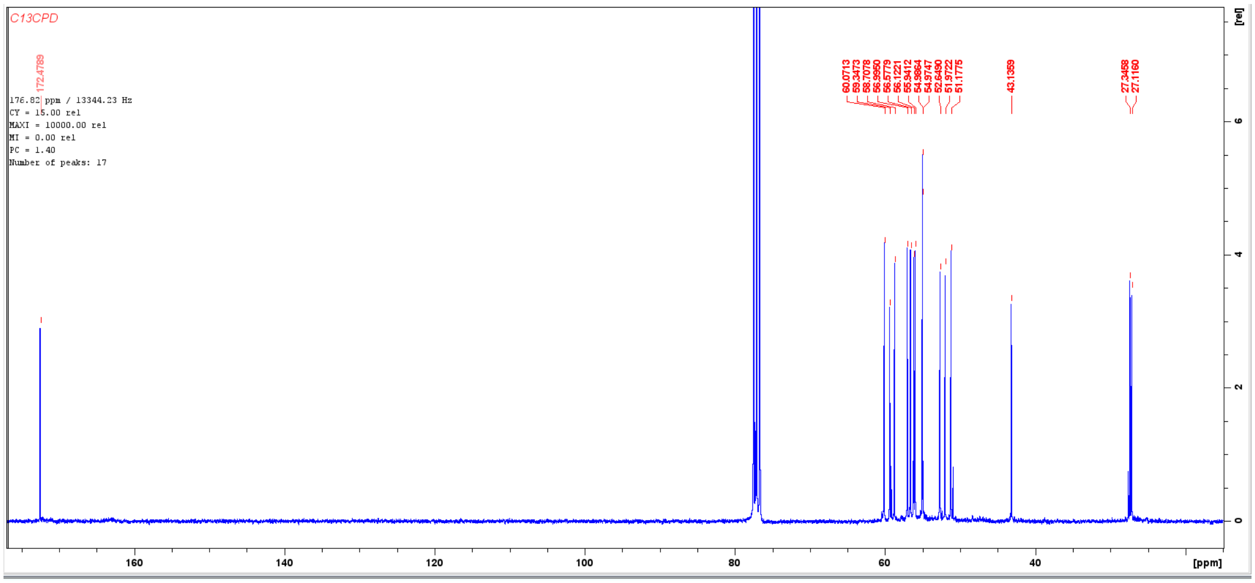

Figure S6. a) ^{1}H NMR (a) and ^{13}C NMR (b) spectra for $\mathbf{L_2}$.

(a)

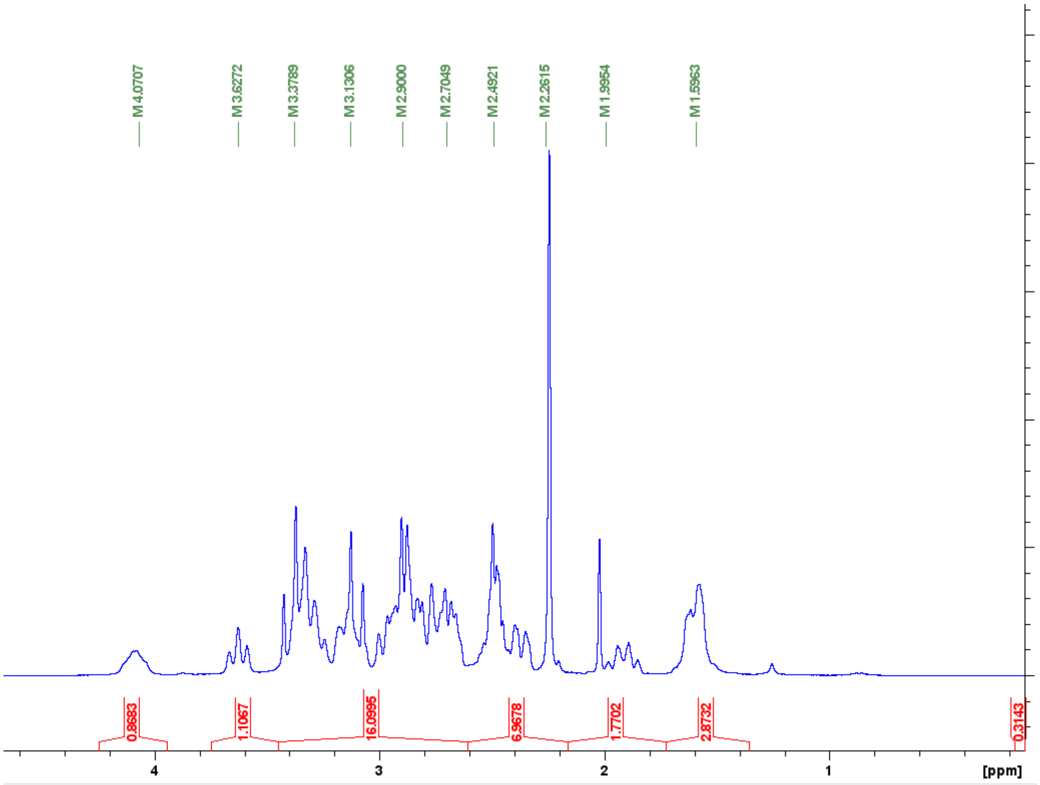

(b)

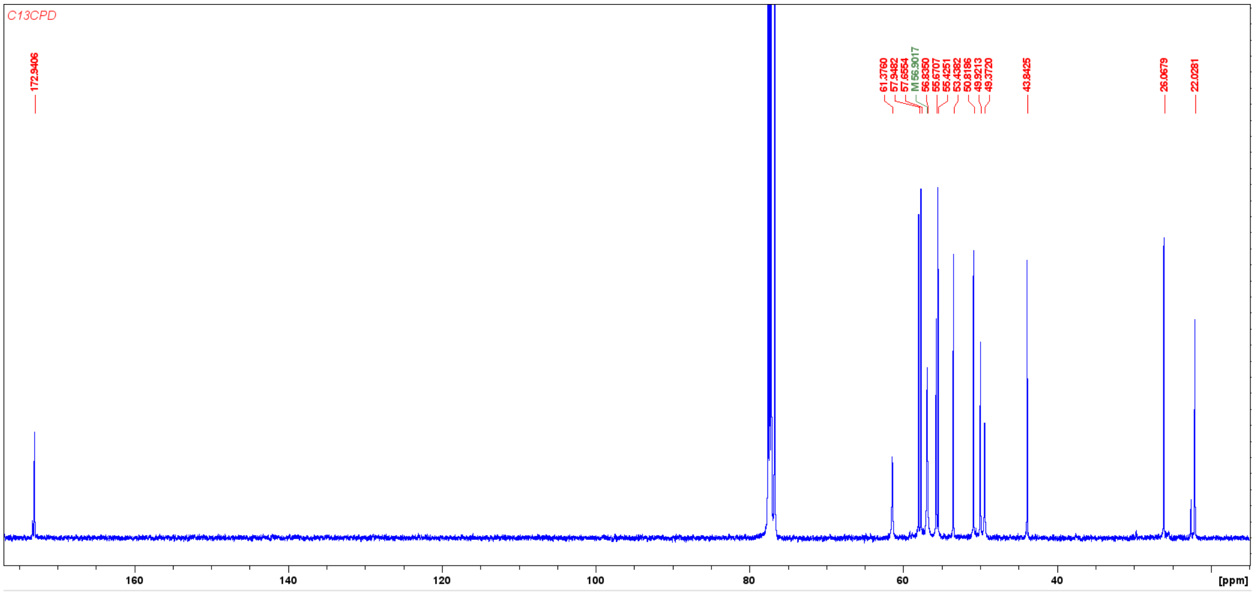

Figure S7. a) ^{1}H NMR (a) and ^{13}C NMR (b) spectra for $L_1ZnClPF_6$.

(a)

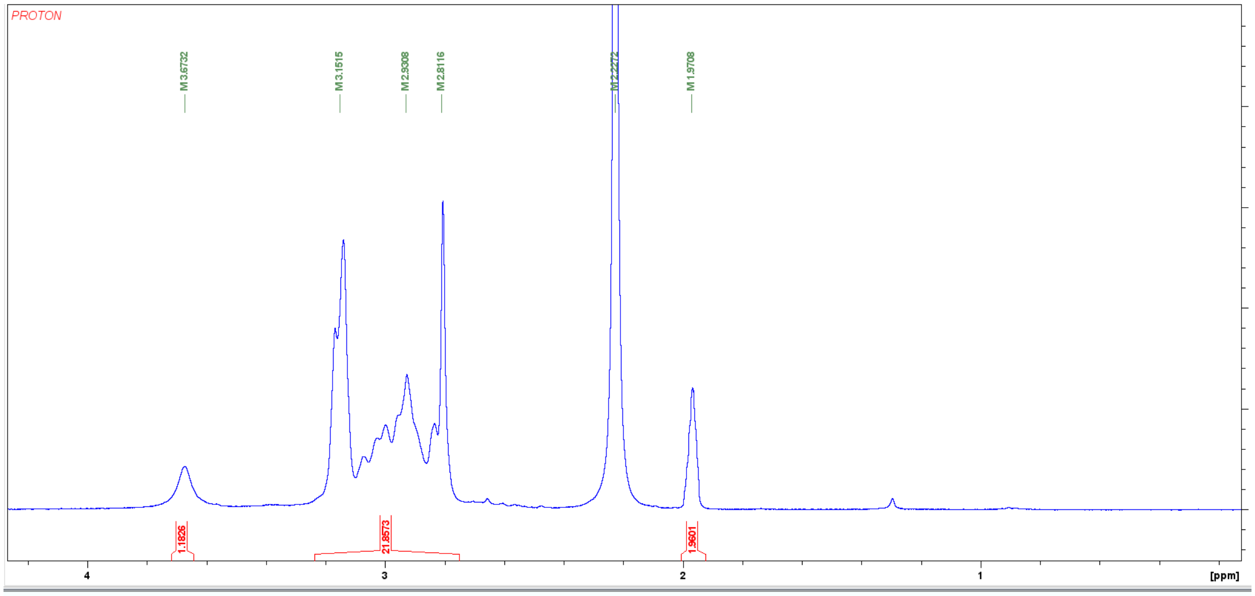

(b)

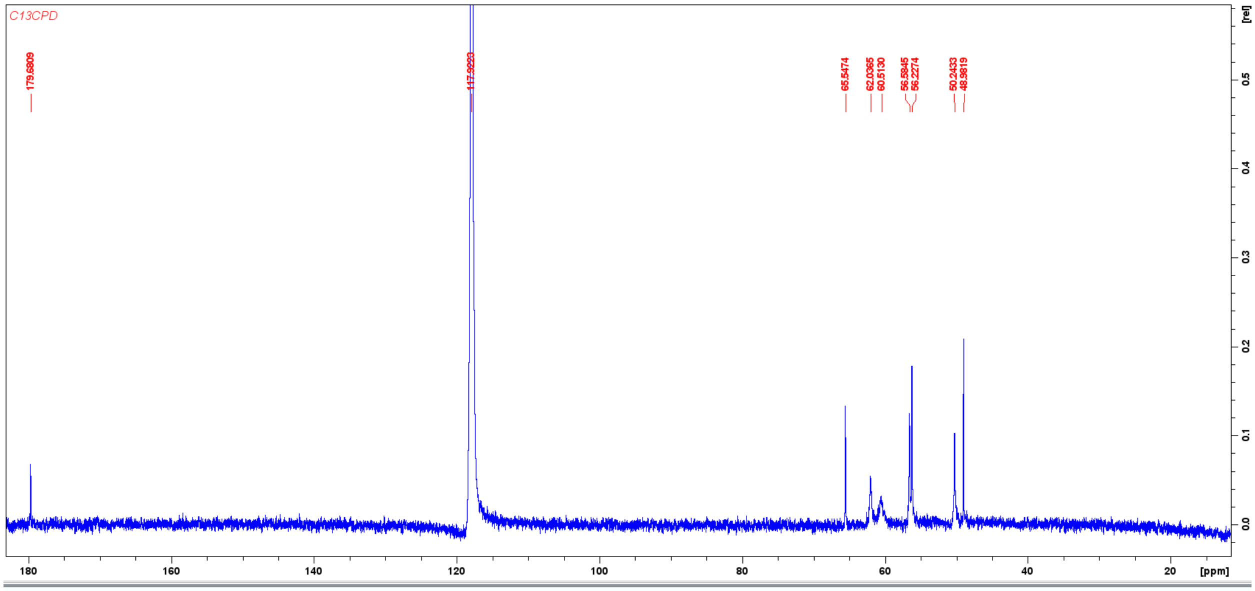

Figure S8. a) ^{1}H NMR (a) and ^{13}C NMR (b) spectra for $L_2ZnClPF_6$.

(a)

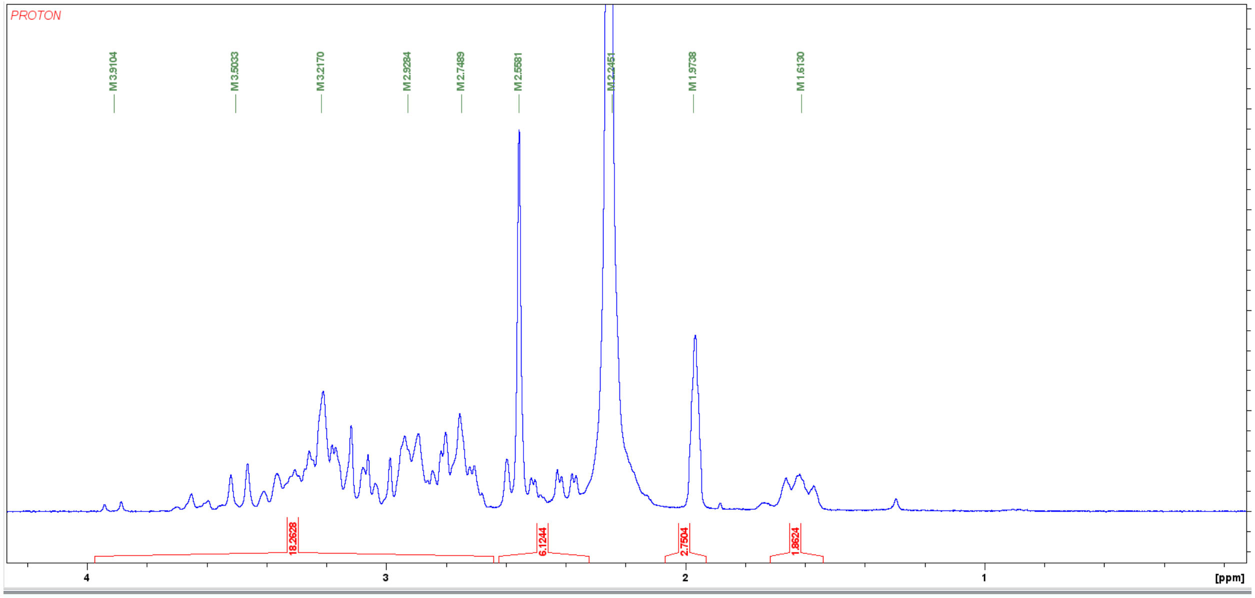

(b)

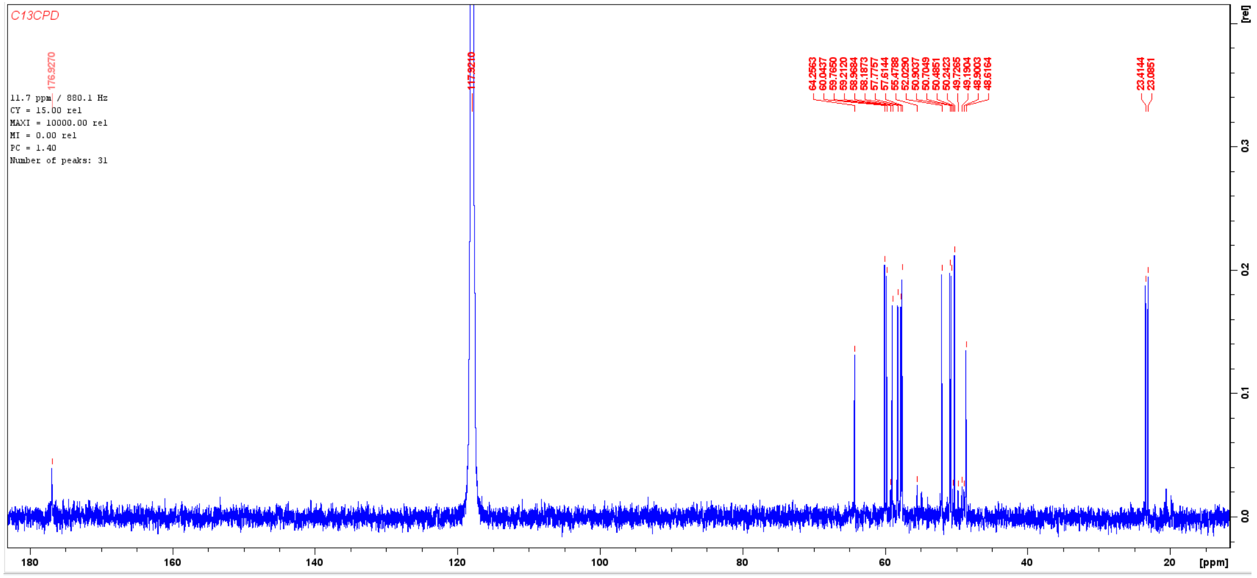

Figure S9. Cyclic Voltammogram in Acetonitrile for MnL1

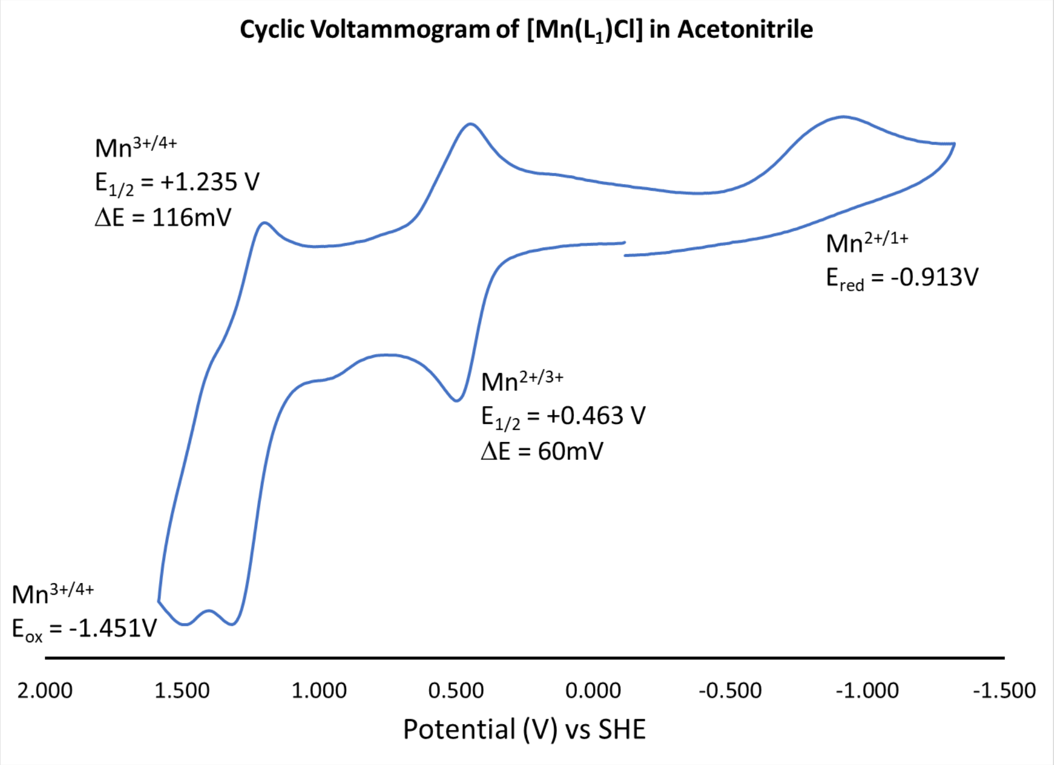

Figure S10. Cyclic Voltammogram in Acetonitrile for FeL1

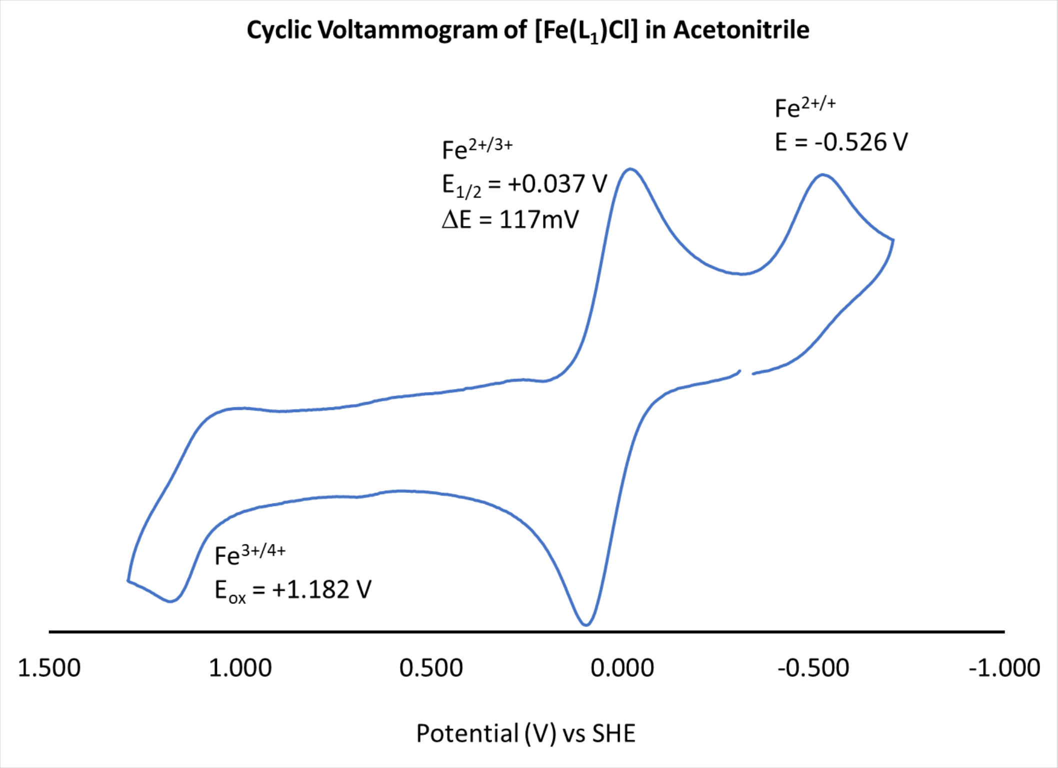

Figure S11. Cyclic Voltammogram in Acetonitrile for CoL1

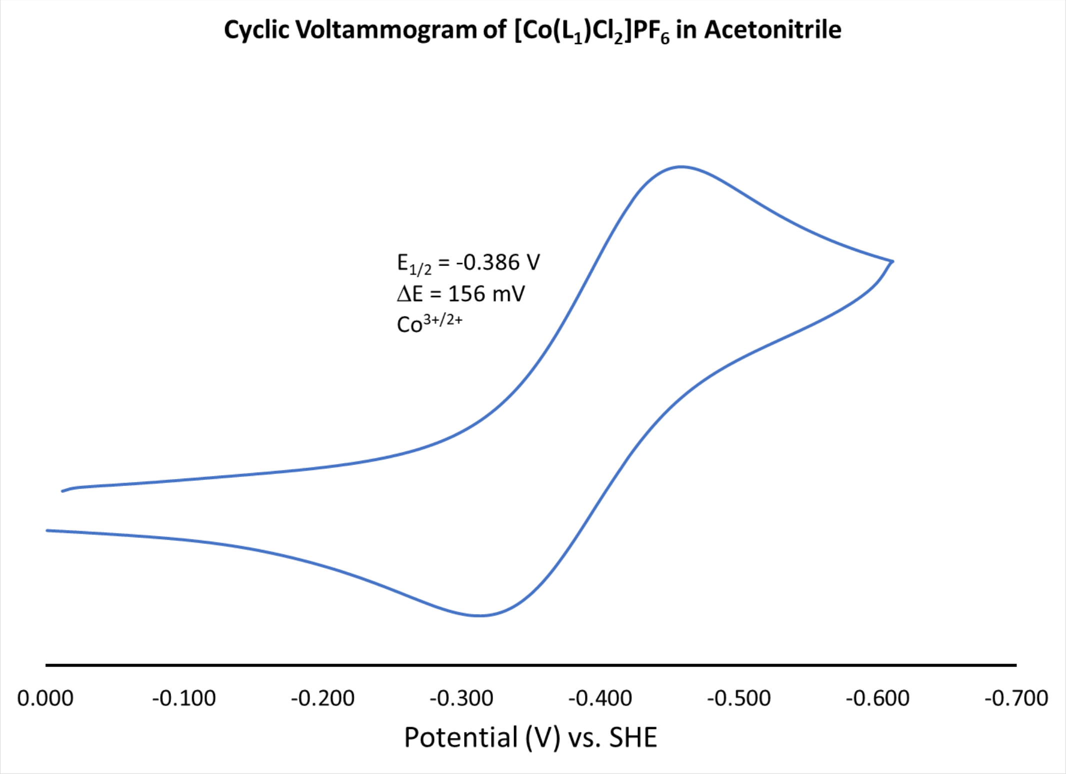

Figure S12. Cyclic Voltammogram in Acetonitrile for NiL1

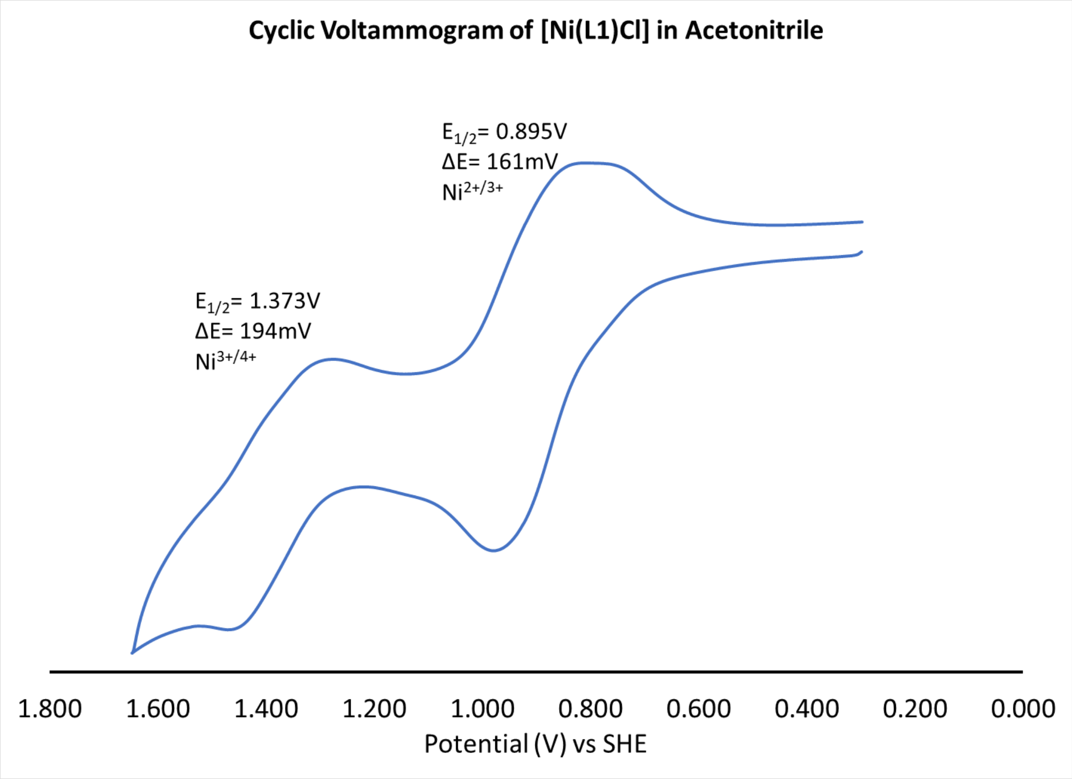

Figure S13. Cyclic Voltammogram in Acetonitrile for CuL1

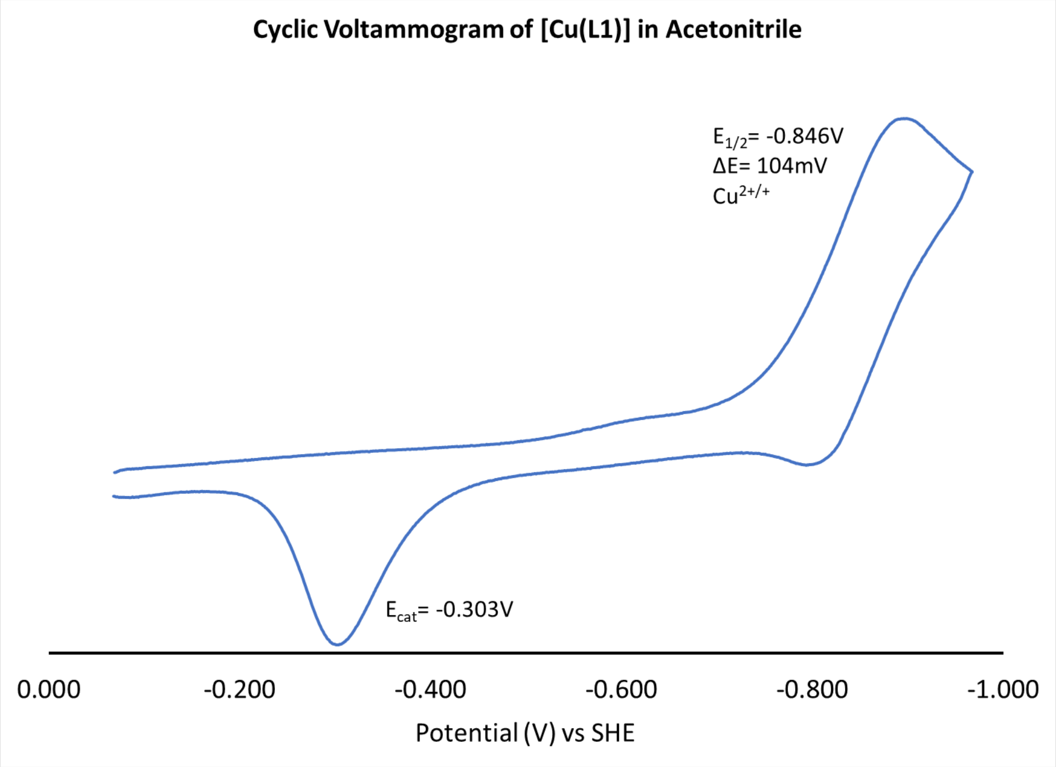

Figure S14. Cyclic Voltammogram in Acetonitrile for MnL2

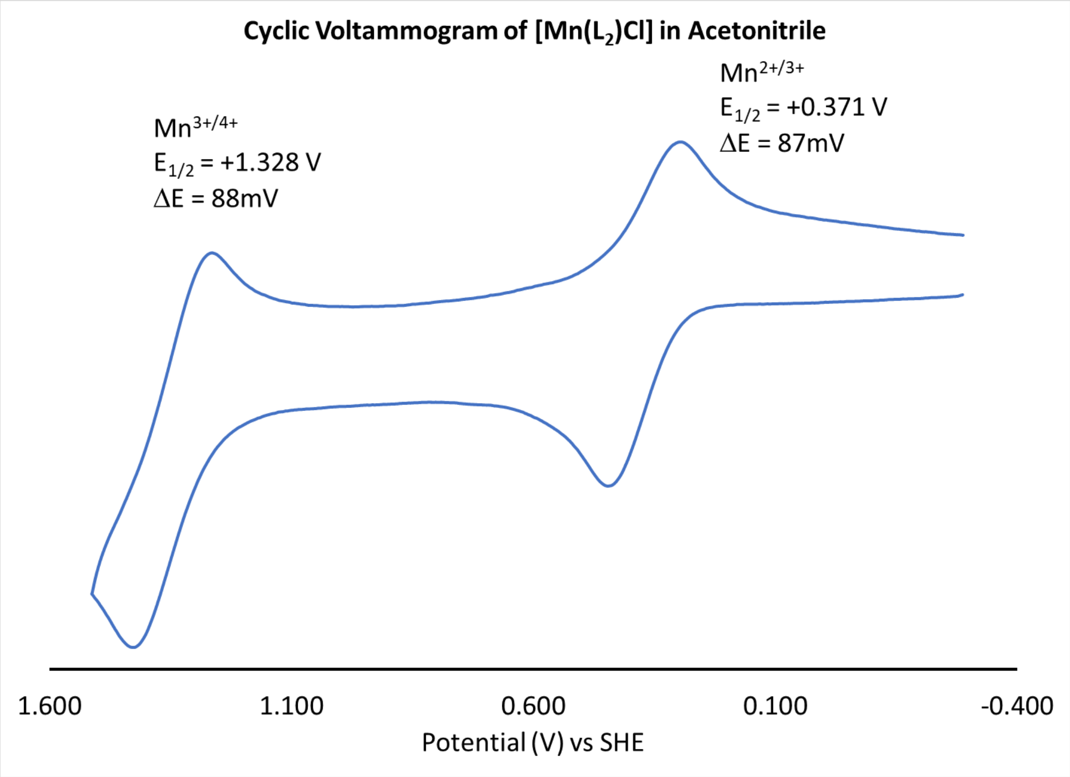

Figure S15. Cyclic Voltammogram in Acetonitrile for FeL2

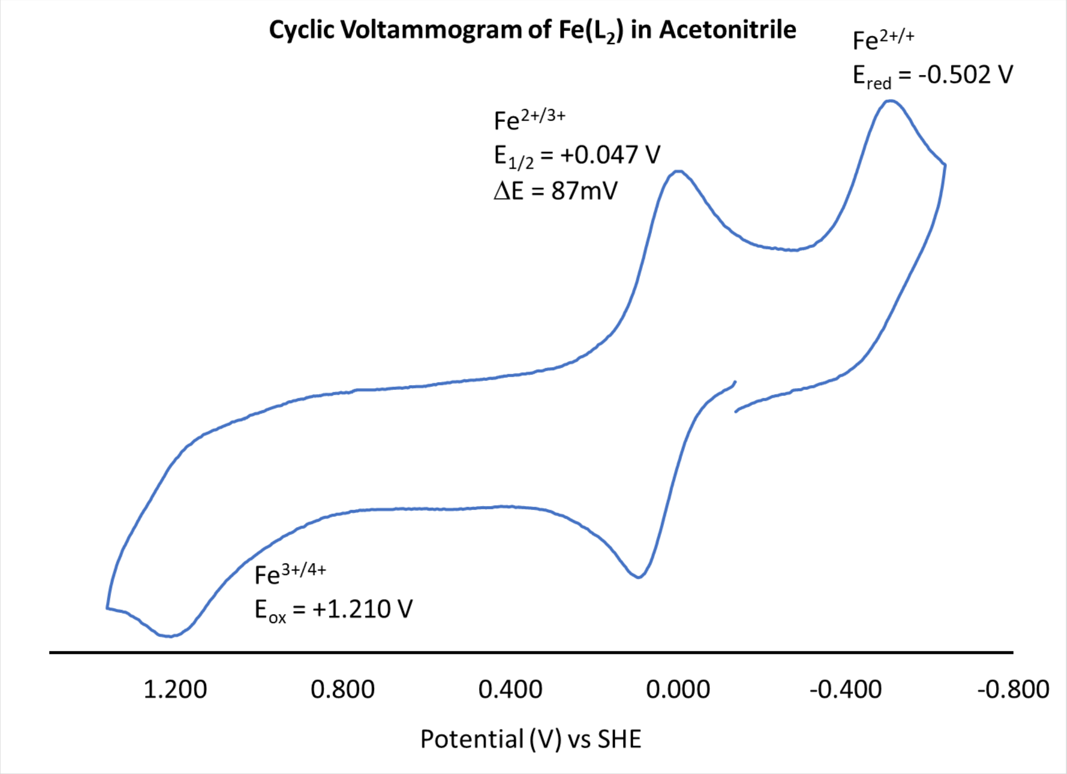

Figure S16. Cyclic Voltammogram in Acetonitrile for CoL2

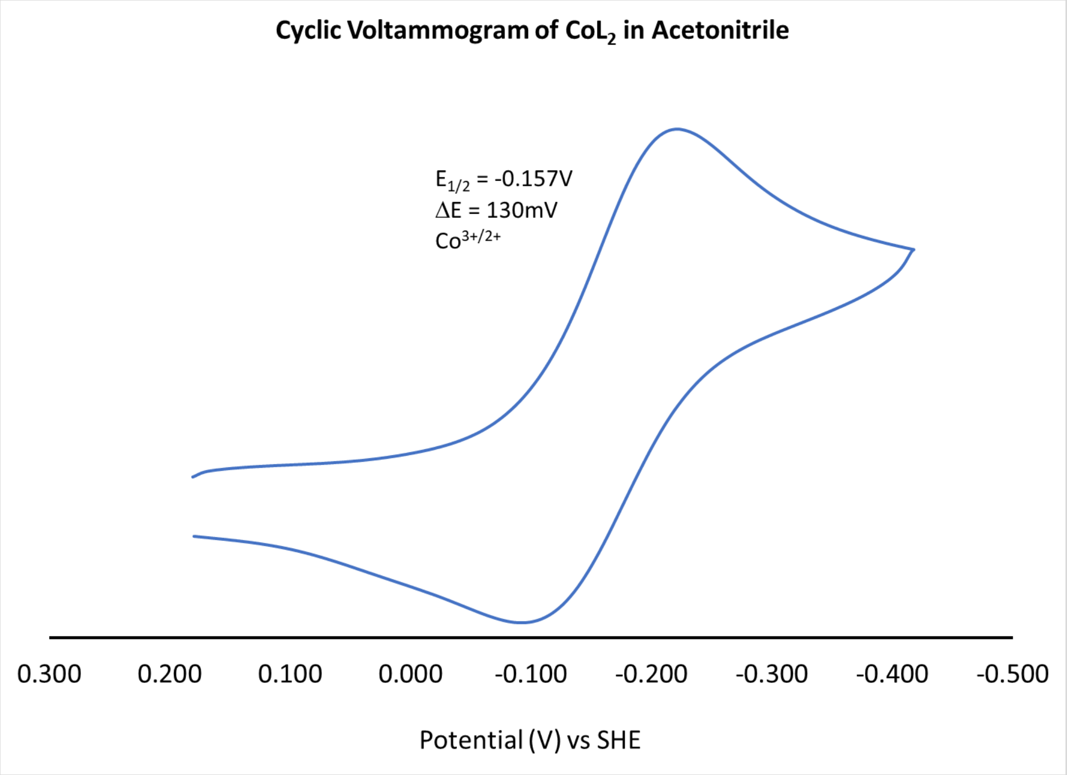

Figure S17. Cyclic Voltammogram in Acetonitrile for NiL2

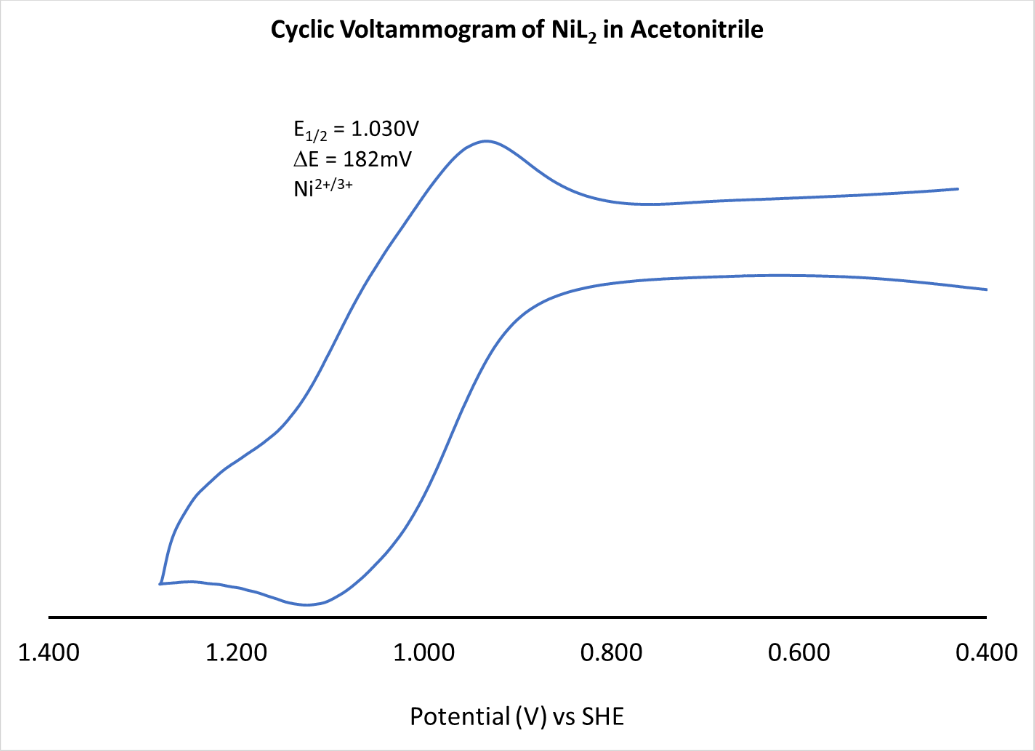

Figure S18. Cyclic Voltammogram in Acetonitrile for CuL2

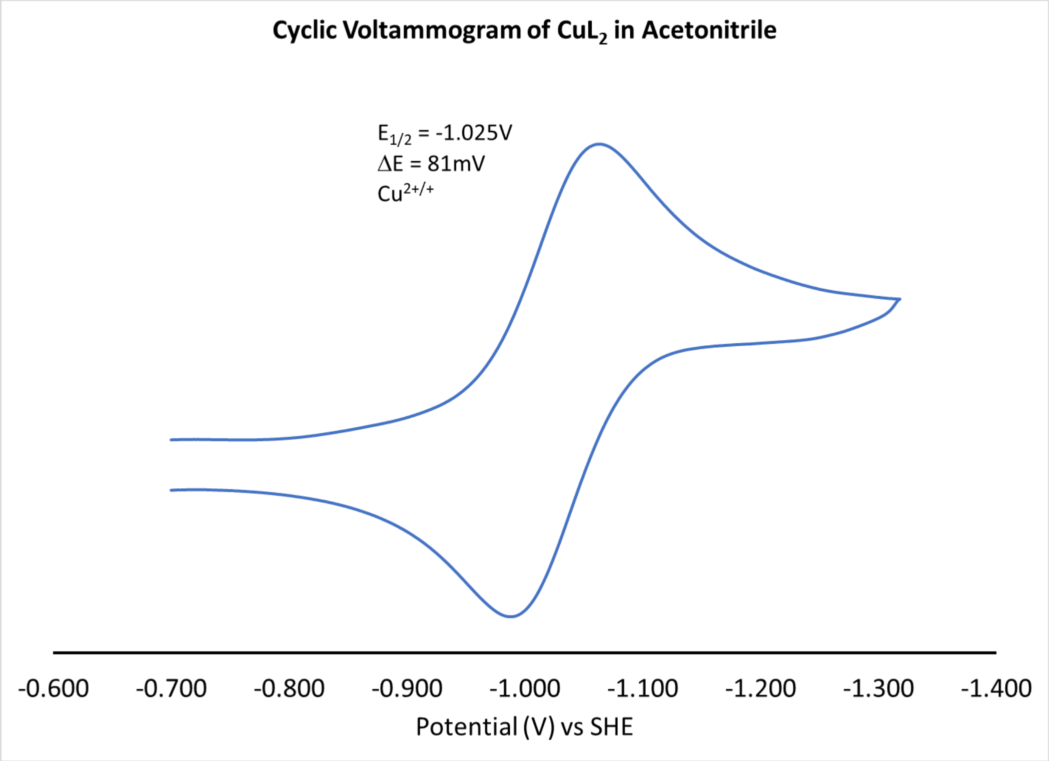

Figure S19. Kinetics of dissociation plots at 40 ^{o}C in 1 M $HClO_4$ for [Cu**L₁**]

(a) Individual UV-Vis Spectra at 2-hour intervals

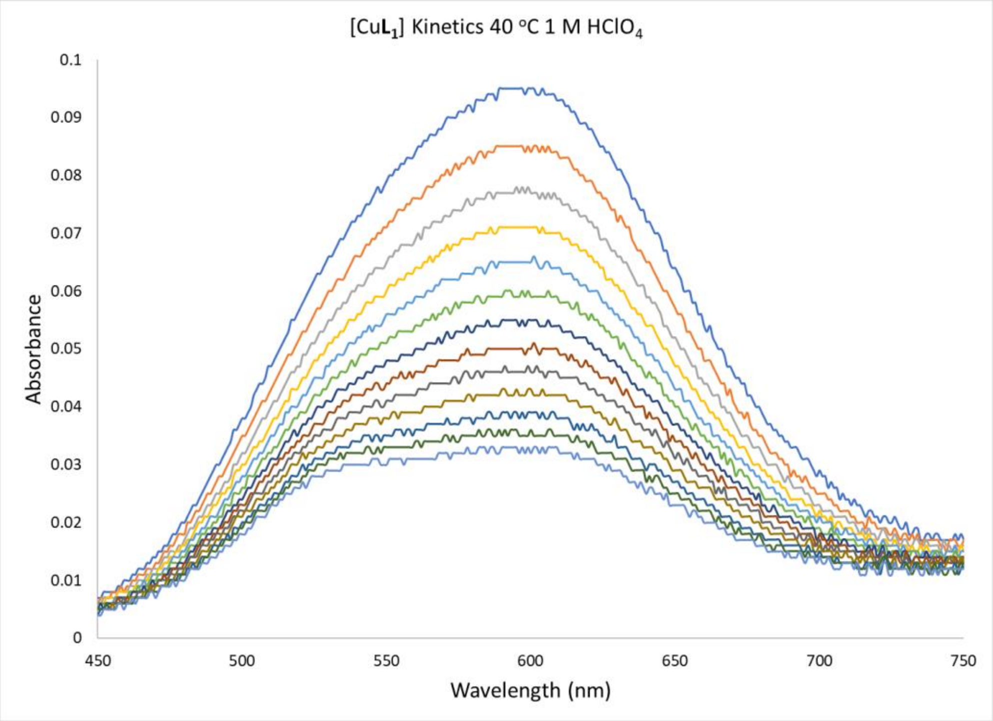

(b) Plot of lnA vs t to Determine Half-Life

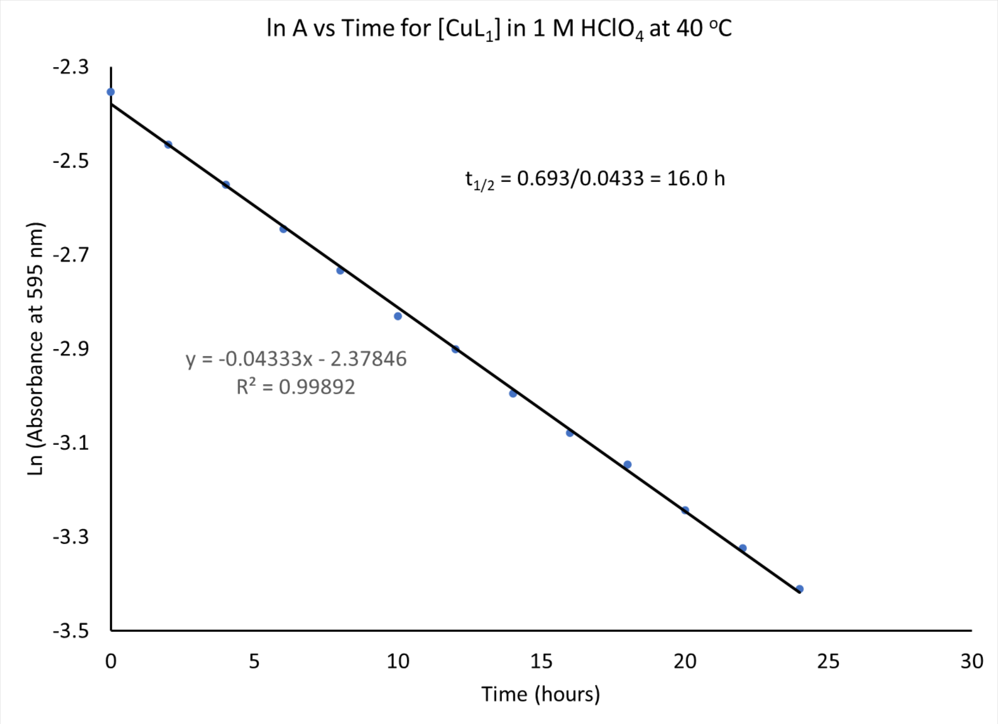

Figure S20. Plot of A_t/A_0 vs time for degradation of Methylene Blue by Fe-L1 complex

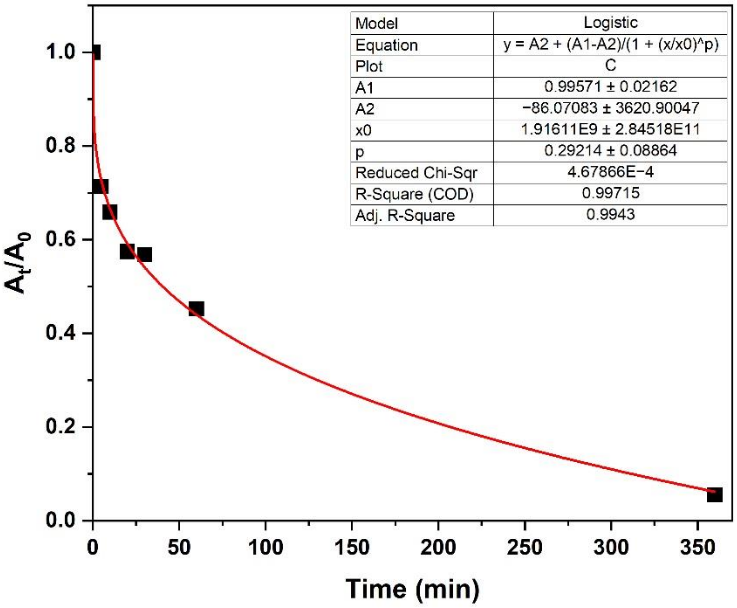

Figure S21. Plot of A_t/A_o vs time for degradation of Methylene Blue by Fe-L2 complex

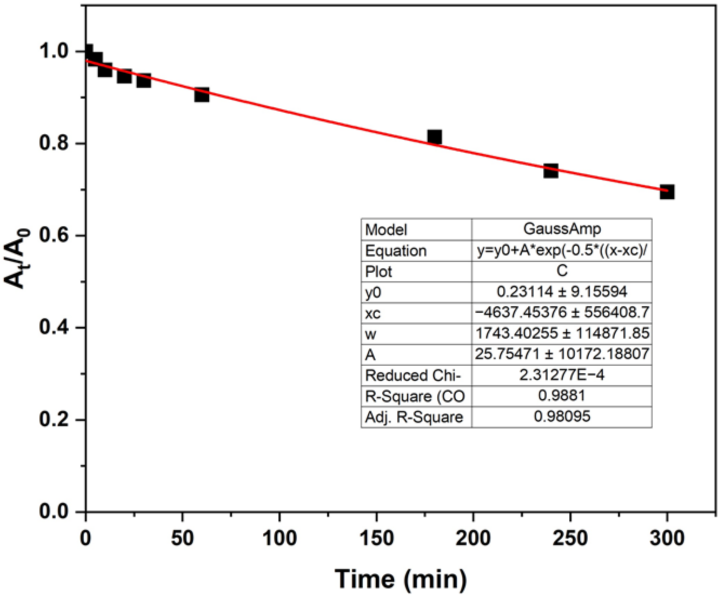

Figure S22. Plot of A_t/A_o vs time for degradation of Methylene Blue by Mn-L1 complex

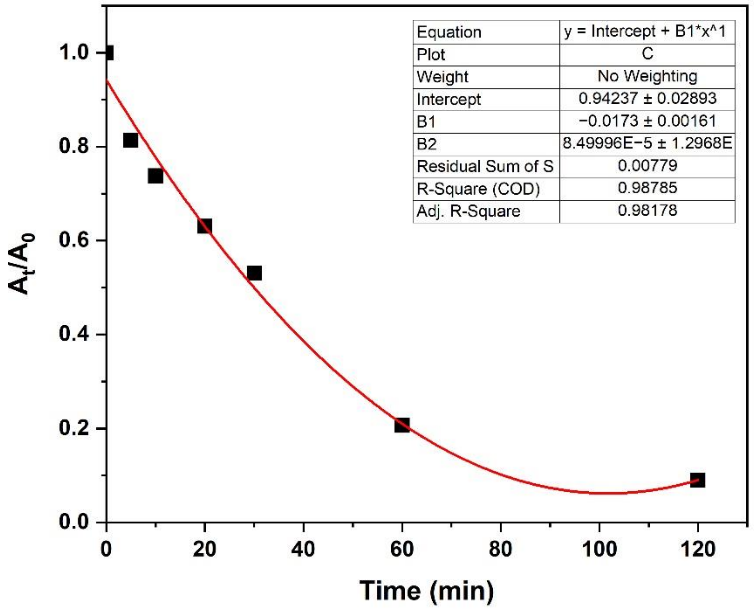

Figure S23. Plot of A_t/A_o vs time for degradation of Methylene Blue by Mn-L2 complex

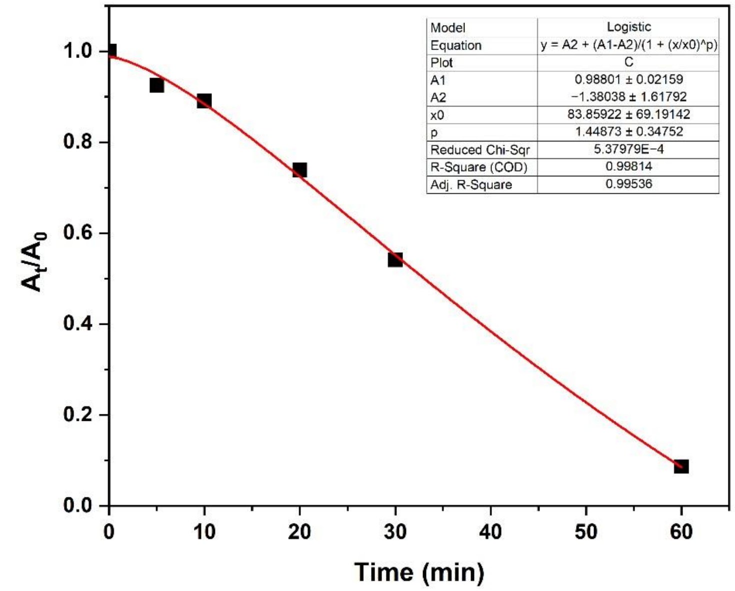

Figure S24. Plot of A_t/A_o vs time for degradation of Methyl Orange by Fe-L1 complex

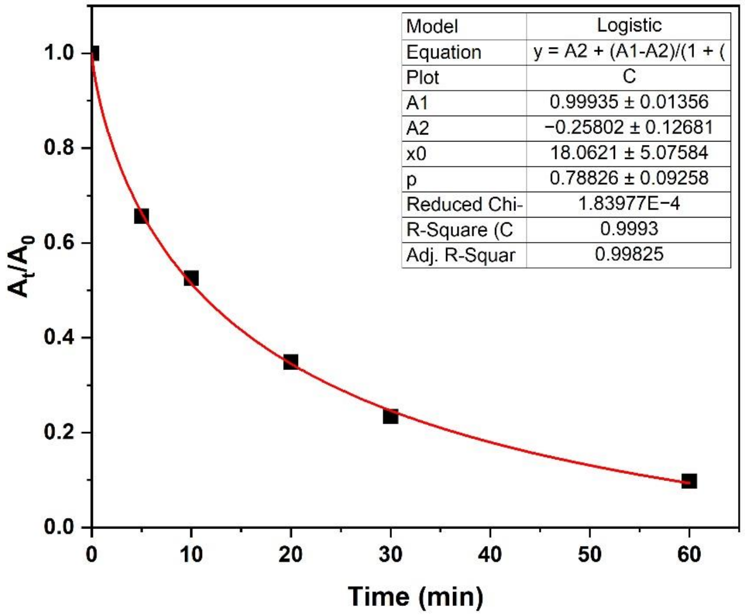

Figure S25. Plot of A_t/A_o vs time for degradation of Methyl Orange by Fe-L2 complex

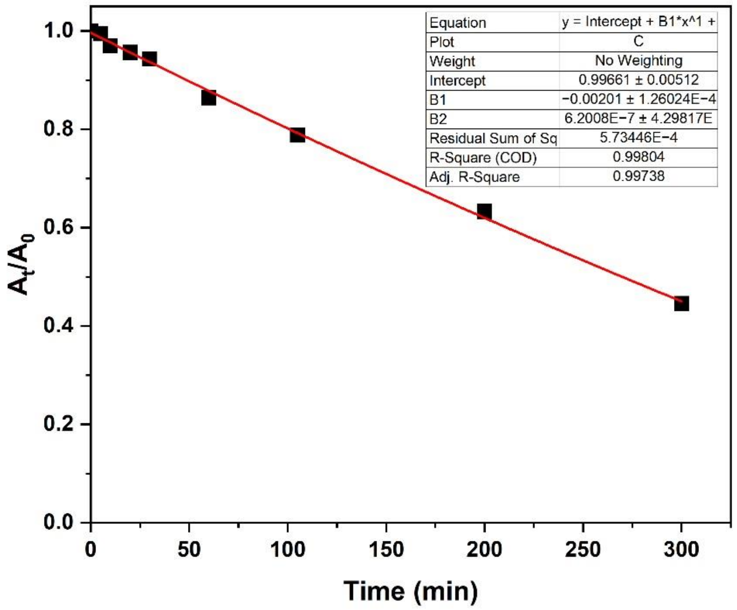

Figure S26. Plot of A_t/A_o vs time for degradation of Methyl Orange by Mn-L1 complex

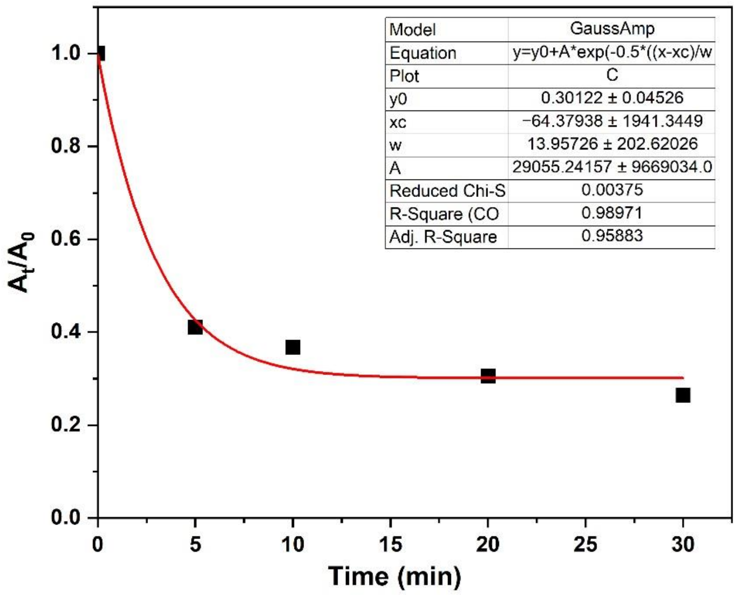

Figure S27. Plot of A_t/A_o vs time for degradation of Methyl Orange by Mn-L2 complex

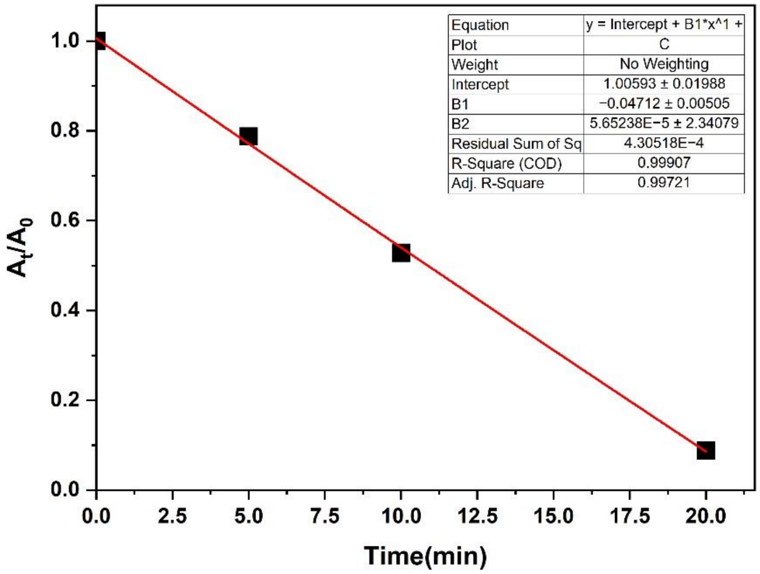

Figure S28. Plot of A_t/A_o vs time for degradation of Rhodamine B by Fe-L1 complex

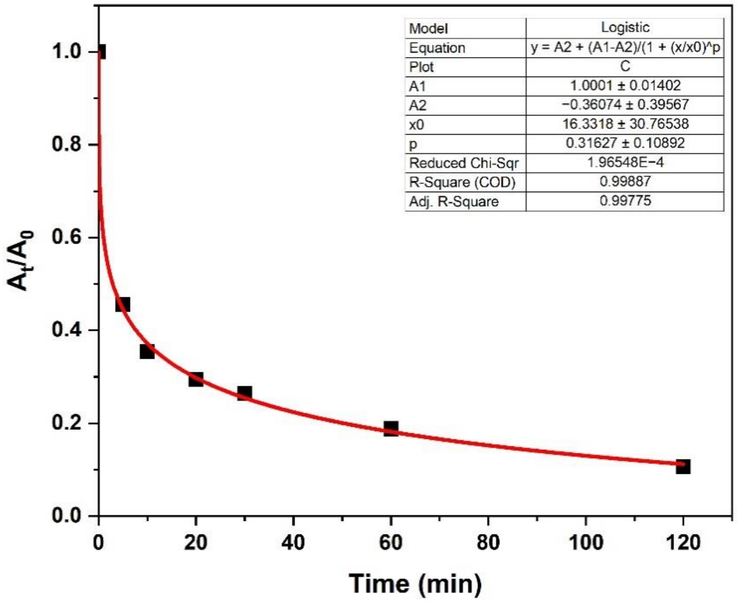

Figure S29. Plot of A_t/A_o vs time for degradation of Rhodamine B by Fe-L2 complex

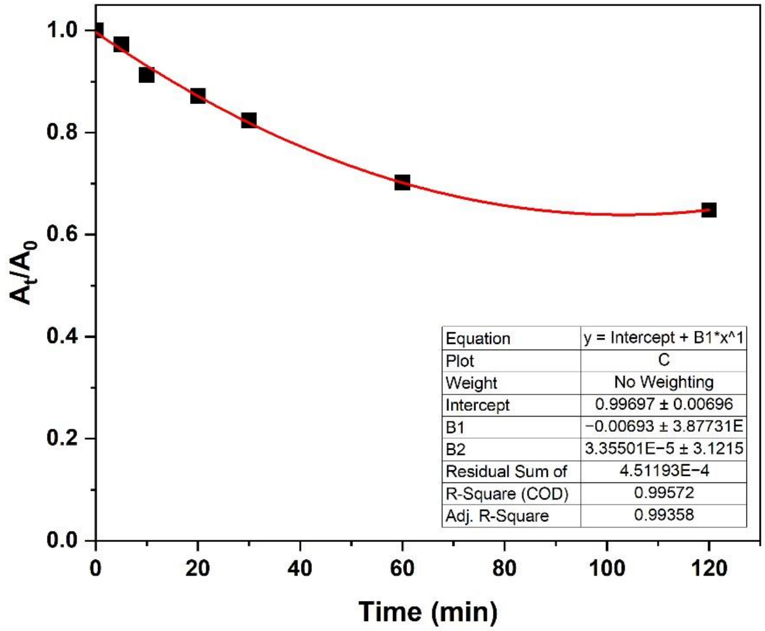

Figure S30. Plot of A_t/A_o vs time for degradation of Rhodamine B by Mn-L1 complex

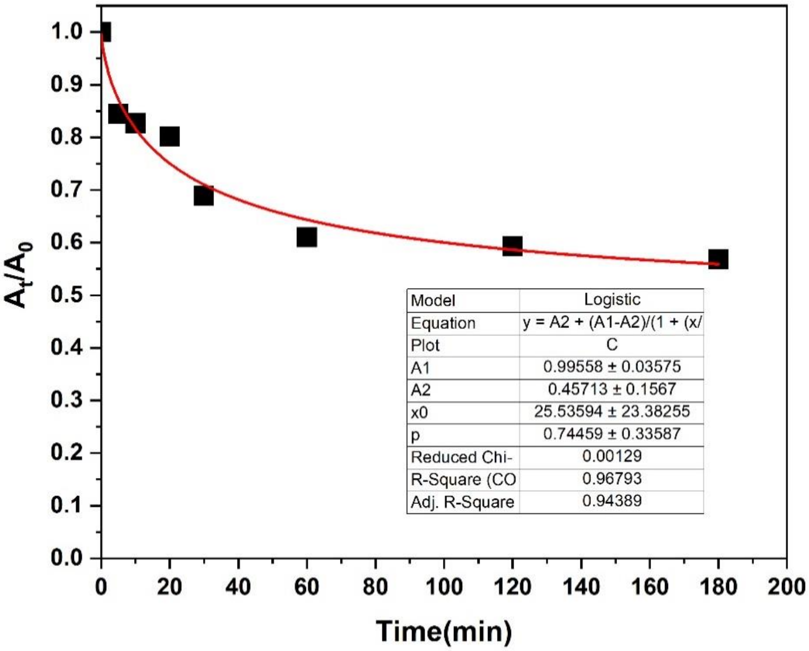

Figure S31. Plot of A_t/A_o vs time for degradation of Rhodamine B by Mn-L2 complex

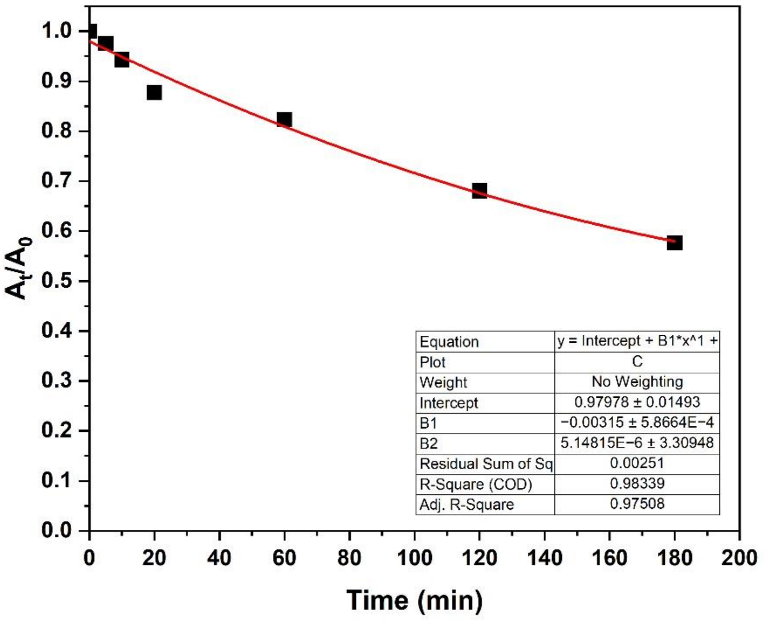

Table S1. Crystal data and structure refinement for [CoL_1Cl]PF_6..

Identification code	th68bp21n
Empirical formula	$C_{13}H_{25}ClCoF_6N_4O_2P$
Structural formula	$C_{13}H_{25}N_4O_2CoCl$, PF_6
Formula weight	508.72
Temperature/K	127
Crystal system	monoclinic
Space group	$P2_1/n$
a/Å	7.9000(2)
b/Å	19.3699(5)
c/Å	12.2722(3)
α/°	90
β/°	98.2620(10)
γ/°	90
Volume/Å^3	1858.43(8)
Z	4
ρ_{calc}g/cm^3	1.818
μ/mm^{-1}	1.230
F(000)	1040.0
Crystal size/mm^3	0.325 × 0.098 × 0.035
Radiation	MoKα (λ = 0.71073Å)
2Θ range for data collection/°	3.958 to 56.564
Index ranges	-10 ≤ h ≤ 10, -25 ≤ k ≤ 25, -14 ≤ l ≤ 16
Reflections collected	45049
Independent reflections	4620 [R_{int} = 0.0546, R_{sigma} = 0.0293]
Data/restraints/parameters	4620/0/254
Goodness-of-fit on F^2	1.177
Final R indexes [I>=2σ (I)]	R_1 = 0.0500, wR_2 = 0.0947
Final R indexes [all data]	R_1 = 0.0599, wR_2 = 0.0979
Largest diff. peak/hole / e Å^{-3}	0.71/-0.58

Table S2. Fractional Atomic Coordinates (×10^4) and Equivalent Isotropic Displacement Parameters ($Å^2$×10^3) for $[CoL_1Cl]PF_6$. U_{eq} is defined as 1/3 of the trace of the orthogonalised U_{IJ} tensor.

Atom	*x*	*y*	*z*	U(eq)
Co1	7559.8(4)	3257.5(2)	1791.5(3)	9.59(9)
Cl1	7886.4(9)	2667.3(4)	222.6(6)	18.47(15)
P1	7320.0(9)	4334.7(4)	6826.9(6)	17.37(16)
F4	9133(2)	4511.5(11)	6440.1(17)	31.3(5)
O1	5183(2)	3367.9(11)	1205.8(17)	17.9(4)
F2	7320(3)	3611.1(11)	6189.7(18)	38.0(5)
F1	5531(3)	4159.4(11)	7220(2)	39.6(5)
O2	3528(3)	3994.9(11)	-53.6(17)	20.8(4)
F5	7372(3)	5042.4(11)	7491(2)	46.7(6)
F6	8287(3)	3960.1(15)	7894.4(17)	54.4(8)
N1	7967(3)	4133.0(12)	1109(2)	14.9(5)
N4	9976(3)	3245.3(12)	2306.6(18)	12.1(4)
F3	6381(3)	4687.8(15)	5757(2)	55.9(7)
N2	7207(3)	3755.2(14)	3103.9(19)	20.6(5)
N3	7326(3)	2413.6(14)	2693(2)	21.8(6)
C12	4935(4)	3842.7(15)	455(2)	15.6(5)
C10	10395(3)	3669.7(15)	3342(2)	16.2(6)
C9	8832(4)	3763.5(18)	3922(2)	22.2(6)
C6	10405(4)	2500.9(14)	2519(2)	16.6(6)
C7	10850(3)	3549.7(16)	1409(2)	17.3(6)
C5	9125(4)	2213.4(16)	3199(3)	22.4(6)
C11	6537(4)	4204.3(15)	170(2)	17.6(6)
C8	9730(4)	4109.3(16)	770(3)	20.5(6)
C1	7832(4)	4697.9(16)	1938(3)	26.3(7)
C3	5833(4)	3365(2)	3560(3)	30.9(8)
C2	6672(4)	4463.6(17)	2738(3)	27.6(7)
C4	6267(4)	2604(2)	3587(3)	35.4(9)
C13	6530(5)	1809.0(19)	2066(3)	37.4(9)

Table S3. Anisotropic Displacement Parameters (Å^2×10^3) for [CoL$_1$Cl]PF$_6$. The Anisotropic displacement factor exponent takes the form: -2π^2[h^{2}a*^{2}U$_{11}$+2hka*b*U$_{12}$+…].

Atom	U_{11}	U_{22}	U_{33}	U_{23}	U_{13}	U_{12}
Co1	8.29(16)	11.21(17)	9.08(16)	1.98(14)	0.55(12)	0.52(13)
Cl1	19.6(3)	19.5(3)	15.3(3)	-5.8(3)	-0.9(2)	2.4(3)
P1	14.8(3)	18.1(4)	19.2(4)	-0.9(3)	2.3(3)	-1.6(3)
F4	22.1(9)	35.1(11)	39.2(12)	-14.0(9)	12.9(8)	-8.9(8)
O1	14.2(9)	21.2(11)	18.5(10)	4.3(8)	2.8(8)	3.1(8)
F2	47.5(13)	28.3(11)	43.1(13)	-14.0(10)	23.2(10)	-12.2(10)
F1	30.0(11)	32.7(11)	61.6(15)	-5.6(10)	25.1(10)	-9.6(9)
O2	16.5(10)	25.5(11)	19.2(10)	-0.8(9)	-1.6(8)	5.2(8)
F5	36.5(12)	31.8(12)	77.7(18)	-32.2(12)	28.0(12)	-12.8(10)
F6	61.2(16)	85.3(19)	19.1(10)	14.1(11)	13.7(10)	45.5(15)
N1	16.1(11)	11.2(11)	16.2(11)	2.4(9)	-1.9(9)	-0.4(9)
N4	11.6(10)	12.8(11)	11.6(10)	0.9(9)	0.4(8)	0.7(9)
F3	47.1(14)	72.3(18)	44.3(14)	28.2(13)	-6.9(11)	17.0(13)
N2	16.5(12)	34.4(15)	10.8(11)	-4.0(11)	1.4(9)	6.5(11)
N3	16.8(12)	24.7(13)	22.7(13)	11.1(11)	-1.6(10)	-5.1(10)
C12	16.0(13)	15.8(13)	14.4(13)	-5.9(10)	0.6(10)	4.0(10)
C10	14.4(13)	18.9(14)	13.7(13)	-2.1(11)	-3.0(10)	-1.2(11)
C9	18.5(14)	34.9(18)	12.1(13)	-4.6(12)	-1.5(11)	6.2(13)
C6	15.7(13)	14.0(13)	18.5(14)	3.1(11)	-3.3(11)	4.5(10)
C7	11.1(12)	25.4(15)	15.7(13)	2.8(11)	3.3(10)	-3.6(11)
C5	21.4(15)	18.8(14)	25.3(16)	9.3(12)	-1.8(12)	-1.4(12)
C11	19.2(14)	15.8(13)	16.6(13)	4.1(11)	-1.7(11)	2.3(11)
C8	15.6(13)	24.2(15)	21.2(14)	11.4(12)	1.0(11)	-4.4(12)
C1	29.5(17)	13.7(14)	31.0(18)	-4.6(13)	-11.9(14)	3.4(12)
C3	17.3(14)	60(2)	16.6(15)	4.7(15)	7.8(12)	2.1(15)
C2	27.2(16)	26.8(17)	26.1(17)	-12.8(14)	-5.5(13)	15.1(14)
C4	21.8(16)	59(3)	26.2(18)	20.9(17)	6.9(13)	-2.0(16)
C13	34.3(19)	26.3(18)	46(2)	14.4(16)	-12.8(16)	-17.8(15)

Table S4. Bond Lengths for [CoL_1Cl]PF_6.

Atom	Atom	Length/Å	Atom	Atom	Length/Å
Co1	Cl1	2.2854(7)	N1	C1	1.508(4)
Co1	O1	1.924(2)	N4	C10	1.509(3)
Co1	N1	1.939(2)	N4	C6	1.495(3)
Co1	N4	1.922(2)	N4	C7	1.503(3)
Co1	N2	1.931(2)	N2	C9	1.512(4)
Co1	N3	1.997(3)	N2	C3	1.496(4)
P1	F4	1.6095(19)	N2	C2	1.486(4)
P1	F2	1.605(2)	N3	C5	1.517(4)
P1	F1	1.594(2)	N3	C4	1.518(4)
P1	F5	1.592(2)	N3	C13	1.490(4)
P1	F6	1.593(2)	C12	C11	1.530(4)
P1	F3	1.569(2)	C10	C9	1.522(4)
O1	C12	1.296(3)	C6	C5	1.508(4)
O2	C12	1.230(3)	C7	C8	1.540(4)
N1	C11	1.500(3)	C1	C2	1.507(5)
N1	C8	1.510(4)	C3	C4	1.513(6)

Table S5. Bond Angles for [CoL_1Cl]PF_6.

Atom	Atom	Atom	Angle/˚	Atom	Atom	Atom	Angle/˚
O1	Co1	Cl1	87.89(7)	C1	N1	Co1	108.14(19)
O1	Co1	N1	87.43(9)	C1	N1	C8	111.4(2)
O1	Co1	N2	90.35(10)	C10	N4	Co1	110.94(16)
O1	Co1	N3	97.77(9)	C6	N4	Co1	104.99(16)
N1	Co1	Cl1	91.60(7)	C6	N4	C10	111.2(2)
N1	Co1	N3	171.70(11)	C6	N4	C7	112.9(2)
N4	Co1	Cl1	92.52(7)	C7	N4	Co1	107.05(16)
N4	Co1	O1	173.64(9)	C7	N4	C10	109.6(2)
N4	Co1	N1	86.22(10)	C9	N2	Co1	110.42(18)
N4	Co1	N2	89.26(10)	C3	N2	Co1	105.0(2)
N4	Co1	N3	88.52(10)	C3	N2	C9	110.5(2)
N2	Co1	Cl1	178.21(8)	C2	N2	Co1	106.15(18)
N2	Co1	N1	88.64(11)	C2	N2	C9	111.6(3)

Table S5. Bond Angles for [CoL_1Cl]PF_6.

Atom	Atom	Atom	Angle/°	Atom	Atom	Atom	Angle/°
N2	Co1	N3	84.87(12)	C2	N2	C3	112.9(3)
N3	Co1	Cl1	95.05(8)	C5	N3	Co1	106.27(17)
F2	P1	F4	88.85(11)	C5	N3	C4	110.4(2)
F1	P1	F4	179.55(14)	C4	N3	Co1	107.8(2)
F1	P1	F2	91.33(11)	C13	N3	Co1	115.0(2)
F5	P1	F4	90.52(11)	C13	N3	C5	108.1(3)
F5	P1	F2	177.99(15)	C13	N3	C4	109.2(3)
F5	P1	F1	89.29(12)	O1	C12	C11	116.2(2)
F5	P1	F6	89.85(15)	O2	C12	O1	124.4(3)
F6	P1	F4	89.85(13)	O2	C12	C11	119.4(3)
F6	P1	F2	88.24(14)	N4	C10	C9	111.4(2)
F6	P1	F1	89.75(14)	N2	C9	C10	110.9(2)
F3	P1	F4	89.67(13)	N4	C6	C5	107.4(2)
F3	P1	F2	90.33(15)	N4	C7	C8	111.0(2)
F3	P1	F1	90.74(14)	C6	C5	N3	110.0(2)
F3	P1	F5	91.57(16)	N1	C11	C12	109.8(2)
F3	P1	F6	178.51(15)	N1	C8	C7	111.5(2)
C12	O1	Co1	112.60(18)	C2	C1	N1	109.3(3)
C11	N1	Co1	104.99(17)	N2	C3	C4	109.1(3)
C11	N1	C8	114.4(2)	N2	C2	C1	107.5(2)
C11	N1	C1	109.4(2)	C3	C4	N3	111.5(3)
C8	N1	Co1	108.03(17)				

Table S6. Torsion Angles for [CoL_1Cl]PF_6.

A	B	C	D	Angle/°	A	B	C	D	Angle/°
Co1	O1	C12	O2	178.3(2)	C10	N4	C7	C8	-87.7(3)
Co1	O1	C12	C11	1.1(3)	C9	N2	C3	C4	-71.1(3)
Co1	N1	C11	C12	30.8(3)	C9	N2	C2	C1	75.7(3)
Co1	N1	C8	C7	-20.4(3)	C6	N4	C10	C9	-95.3(3)
Co1	N1	C1	C2	-26.6(3)	C6	N4	C7	C8	147.7(2)
Co1	N4	C10	C9	21.1(3)	C7	N4	C10	C9	139.1(2)
Co1	N4	C6	C5	-49.1(2)	C7	N4	C6	C5	-165.3(2)
Co1	N4	C7	C8	32.6(3)	C5	N3	C4	C3	112.0(3)
Co1	N2	C9	C10	24.4(3)	C11	N1	C8	C7	-136.9(3)
Co1	N2	C3	C4	47.9(3)	C11	N1	C1	C2	87.2(3)
Co1	N2	C2	C1	-44.7(3)	C8	N1	C11	C12	149.0(2)
Co1	N3	C5	C6	-23.4(3)	C8	N1	C1	C2	-145.2(2)

Table S6. Torsion Angles for $[CoL_1Cl]PF_6$.

A	B	C	D	Angle/˚	A	B	C	D	Angle/˚
Co1	N3	C4	C3	-3.7(3)	C1	N1	C11	C12	-85.1(3)
O1	C12	C11	N1	-22.3(3)	C1	N1	C8	C7	98.3(3)
O2	C12	C11	N1	160.3(2)	C3	N2	C9	C10	140.1(3)
N1	C1	C2	N2	47.3(3)	C3	N2	C2	C1	-159.2(3)
N4	C10	C9	N2	-29.5(3)	C2	N2	C9	C10	-93.5(3)
N4	C6	C5	N3	48.4(3)	C2	N2	C3	C4	163.1(3)
N4	C7	C8	N1	-7.9(3)	C4	N3	C5	C6	-140.0(3)
N2	C3	C4	N3	-28.5(4)	C13	N3	C5	C6	100.6(3)
C10	N4	C6	C5	71.0(3)	C13	N3	C4	C3	-129.3(3)

Table S7. Hydrogen Atom Coordinates (Å×10^4) and Isotropic Displacement Parameters (Å^2×10^3) for$[CoL_1Cl]PF_6$.

Atom	*x*	*y*	*z*	U(eq)
H10A	10823.88	4128.05	3151.88	19
H10B	11311.63	3438.11	3845.91	19
H9A	8792.75	3387.83	4464.87	27
H9B	8920.99	4207.83	4324.77	27
H6A	11578.83	2455.17	2920.69	20
H6B	10340.85	2246.74	1813.95	20
H7A	11088.37	3179.95	894.78	21
H7B	11955.95	3754.42	1734.56	21
H5A	9365.84	2395.76	3958.9	27
H5B	9224.31	1704.13	3233.45	27
H11A	6878.4	3995.98	-501.73	21
H11B	6289.55	4699.24	22.61	21
H8A	10287.97	4564.84	907.81	25
H8B	9627.06	4013.03	-29.33	25
H1A	8980.6	4805.38	2338.77	32
H1B	7366	5121.58	1555.09	32
H3A	4718.94	3442.23	3092.91	37
H3B	5742.16	3529.82	4312.72	37
H2A	6769.66	4779.78	3378.15	33
H2B	5467.44	4461.83	2377.75	33

Table S7. Hydrogen Atom Coordinates (Å×10^4) and Isotropic Displacement Parameters (Å^2×10^3) for[CoL_1Cl]PF_6.

Atom	*x*	*y*	*z*	U(eq)
H4A	5195.79	2330.97	3487.39	42
H4B	6911.6	2485.83	4315.81	42
H13A	5421.38	1944.51	1658.08	56
H13B	6363.88	1435.73	2579	56
H13C	7281.2	1649.31	1547.9	56

Table S8. Crystal data and structure refinement for [CuL_1]PF_6. .

Identification code	th70bp1
Empirical formula	$C_{13}H_{25}CuF_6N_4O_2P$
Structural formula	$C_{13}H_{25}N_4O_2Cu$, PF_6
Formula weight	477.88
Temperature/K	120
Crystal system	triclinic
Space group	P-1
a/Å	8.2596(8)
b/Å	8.7606(9)
c/Å	13.0051(12)
α/°	77.500(3)
β/°	81.329(3)
γ/°	87.627(3)
Volume/Å^3	908.19(15)
Z	2
ρ_{calc}g/cm^3	1.748
μ/mm^{-1}	1.367
F(000)	490.0
Crystal size/mm^3	0.451 × 0.097 × 0.018
Radiation	MoKα (λ = 0.71073 Å)
2Θ range for data collection/°	4.762 to 56.614
Index ranges	-11 ≤ h ≤ 10, -11 ≤ k ≤ 11, -17 ≤ l ≤ 17
Reflections collected	24102
Independent reflections	4507 [R_{int} = 0.0283, R_{sigma} = 0.0217]
Data/restraints/parameters	4507/0/245
Goodness-of-fit on F^2	1.029
Final R indexes [I>=2σ (I)]	R_1 = 0.0238, wR_2 = 0.0551
Final R indexes [all data]	R_1 = 0.0304, wR_2 = 0.0574
Largest diff. peak/hole / e Å^{-3}	0.51/-0.33

Table S9 Fractional Atomic Coordinates (×10^4) and Equivalent Isotropic Displacement Parameters (Å^2×10^3) for [CuL_1]PF_6. U_{eq} is defined as 1/3 of the trace of the orthogonalised U_{IJ} tensor.

Atom	*x*	*y*	*z*	U(eq)
Cu1	2448.5(2)	6898.7(2)	3462.5(2)	10.12(5)
P1	2288.7(5)	7403.2(5)	8498.6(3)	14.13(9)
F1	2541.9(13)	6194.3(11)	7722.7(8)	23.5(2)
F6	2053.9(14)	8623.5(12)	9269.7(8)	27.4(2)
F4	2291.2(14)	5984.9(12)	9497.6(8)	27.1(2)
F3	2301.3(15)	8830.0(12)	7492.6(8)	29.3(2)
F2	351.6(13)	7286.8(14)	8576.4(9)	31.4(3)
O1	2354.6(13)	4953.8(12)	4537.6(9)	14.7(2)
O2	956.7(15)	2724.1(13)	5005.5(10)	21.6(3)
F5	4238.2(13)	7536.7(12)	8410.2(10)	32.5(3)
N3	3666.0(16)	8234.8(15)	4134.6(10)	13.6(3)
N2	4505.6(16)	7303.5(15)	2163.4(10)	13.8(3)
N1	1482.7(15)	5686.0(14)	2605.8(10)	10.9(2)
N4	1609.3(16)	8885.5(14)	2538.2(10)	12.8(2)
C8	503.4(19)	6777.6(17)	1879.7(12)	13.1(3)
C12	1329.9(19)	3980.0(17)	4392.4(13)	14.3(3)
C11	493.4(19)	4484.1(17)	3395.4(12)	13.8(3)
C6	1427(2)	9892.4(17)	3330.8(13)	16.1(3)
C7	77.7(18)	8263.6(17)	2313.3(13)	14.9(3)
C9	4492(2)	8922.9(18)	1572.3(13)	17.4(3)
C1	2935.0(18)	4965.1(17)	2055.3(12)	13.9(3)
C13	3342(2)	7803(2)	5311.9(13)	20.2(3)
C4	5454.1(19)	7976.6(19)	3740.8(13)	17.7(3)
C3	5730.4(19)	6960.1(19)	2893.8(13)	17.9(3)
C5	3057(2)	9886.8(17)	3760.5(13)	15.8(3)
C2	4271.7(19)	6161.5(18)	1513.5(13)	15.6(3)
C10	2725(2)	9544.8(18)	1548.1(13)	16.7(3)

Table S10. Anisotropic Displacement Parameters (Å^2×10^3) for [CuL_1]PF_6. The Anisotropic displacement factor exponent takes the form: $-2\pi^2[h^2a^{*2}U_{11}+2hka^*b^*U_{12}+\ldots]$.

Atom	U_{11}	U_{22}	U_{33}	U_{23}	U_{13}	U_{12}
Cu1	11.28(9)	9.21(8)	10.46(9)	-2.37(6)	-2.73(6)	-1.21(6)
P1	17.0(2)	13.59(18)	12.5(2)	-3.74(14)	-2.95(15)	0.83(14)
F1	34.6(6)	19.2(5)	20.2(5)	-10.3(4)	-7.3(4)	3.8(4)
F6	42.7(6)	22.3(5)	20.9(5)	-11.9(4)	-7.8(5)	6.3(4)
F4	42.4(6)	20.2(5)	17.2(5)	1.0(4)	-7.3(5)	2.0(4)

Table S10. Anisotropic Displacement Parameters (Å^2×10^3) for [CuL$_1$]PF$_6$. The Anisotropic displacement factor exponent takes the form: $-2\pi^2[h^2a^{*2}U_{11}+2hka^*b^*U_{12}+…]$.

Atom	U_{11}	U_{22}	U_{33}	U_{23}	U_{13}	U_{12}
F3	48.9(7)	17.8(5)	16.9(5)	0.4(4)	1.2(5)	7.8(5)
F2	16.8(5)	39.6(6)	39.3(7)	-11.1(5)	-5.8(5)	1.8(4)
O1	16.8(5)	13.4(5)	14.3(5)	-2.2(4)	-4.4(4)	-1.1(4)
O2	25.8(6)	14.8(5)	22.2(6)	1.9(5)	-4.9(5)	-4.8(5)
F5	17.7(5)	25.4(5)	56.8(8)	-13.8(5)	-5.2(5)	-2.6(4)
N3	15.0(6)	13.1(6)	13.1(6)	-3.1(5)	-2.8(5)	-2.0(5)
N2	13.1(6)	14.0(6)	14.6(6)	-3.3(5)	-2.3(5)	-1.5(5)
N1	11.5(6)	10.6(5)	11.0(6)	-3.0(5)	-1.9(5)	0.7(4)
N4	14.0(6)	11.0(6)	14.3(6)	-3.4(5)	-3.8(5)	0.5(5)
C8	14.6(7)	13.2(7)	12.9(7)	-3.5(5)	-5.5(6)	1.2(5)
C12	14.3(7)	13.0(7)	15.3(8)	-4.2(6)	0.1(6)	1.2(5)
C11	14.4(7)	12.2(7)	15.2(8)	-3.4(6)	-1.8(6)	-3.7(5)
C6	19.7(8)	11.5(7)	18.4(8)	-5.9(6)	-3.5(6)	1.4(6)
C7	11.8(7)	15.0(7)	19.4(8)	-4.9(6)	-6.2(6)	2.1(5)
C9	18.8(8)	16.5(7)	15.2(8)	-1.3(6)	1.4(6)	-3.9(6)
C1	14.8(7)	12.7(7)	15.2(7)	-5.8(6)	-1.8(6)	2.7(5)
C13	25.7(9)	23.7(8)	13.1(8)	-5.4(6)	-4.6(7)	-6.0(7)
C4	13.1(7)	20.8(8)	21.1(8)	-6.1(6)	-4.9(6)	-2.5(6)
C3	10.6(7)	22.6(8)	21.8(8)	-7.2(6)	-3.1(6)	0.2(6)
C5	19.5(8)	11.1(7)	18.1(8)	-5.4(6)	-3.1(6)	-1.5(6)
C2	14.8(7)	17.9(7)	15.2(8)	-7.1(6)	-1.1(6)	0.8(6)
C10	21.0(8)	13.7(7)	14.3(8)	0.7(6)	-3.6(6)	-1.6(6)

Table S11. Bond Lengths for [CuL$_1$]PF$_6$.

Atom	Atom	Length/Å	Atom	Atom	Length/Å
Cu1	O1	1.9522(11)	N2	C9	1.4605(19)
Cu1	N3	1.9876(13)	N2	C3	1.469(2)
Cu1	N2	2.1891(13)	N2	C2	1.4778(19)
Cu1	N1	1.9640(12)	N1	C8	1.4953(18)
Cu1	N4	2.0495(13)	N1	C11	1.4769(18)
P1	F1	1.6013(10)	N1	C1	1.4914(19)
P1	F6	1.6045(10)	N4	C6	1.4830(19)
P1	F4	1.5917(10)	N4	C7	1.4900(19)
P1	F3	1.6010(11)	N4	C10	1.485(2)
P1	F2	1.5940(11)	C8	C7	1.535(2)
P1	F5	1.6043(11)	C12	C11	1.533(2)

Table S11. Bond Lengths for [CuL$_1$]PF$_6$.

Atom	Atom	Length/Å	Atom	Atom	Length/Å
O1	C12	1.2874(18)	C6	C5	1.533(2)
O2	C12	1.2311(19)	C9	C10	1.539(2)
N3	C13	1.481(2)	C1	C2	1.535(2)
N3	C4	1.513(2)	C4	C3	1.546(2)
N3	C5	1.5144(19)			

Table S12. Bond Angles for [CuL$_1$]PF$_6$.

Atom	Atom	Atom	Angle/˚	Atom	Atom	Atom	Angle/˚
O1	Cu1	N3	100.26(5)	C9	N2	Cu1	110.33(10)
O1	Cu1	N2	119.60(5)	C9	N2	C3	114.38(12)
O1	Cu1	N1	85.39(5)	C9	N2	C2	113.12(13)
O1	Cu1	N4	158.06(5)	C3	N2	Cu1	93.03(9)
N3	Cu1	N2	85.38(5)	C3	N2	C2	118.76(12)
N3	Cu1	N4	88.88(5)	C2	N2	Cu1	104.42(9)
N1	Cu1	N3	171.41(5)	C8	N1	Cu1	108.29(9)
N1	Cu1	N2	86.20(5)	C11	N1	Cu1	104.55(9)
N1	Cu1	N4	88.09(5)	C11	N1	C8	113.88(12)
N4	Cu1	N2	80.73(5)	C11	N1	C1	110.87(11)
F1	P1	F6	179.36(7)	C1	N1	Cu1	103.45(9)
F1	P1	F5	89.74(6)	C1	N1	C8	114.71(12)
F4	P1	F1	89.81(6)	C6	N4	Cu1	98.37(9)
F4	P1	F6	90.51(6)	C6	N4	C7	116.97(12)
F4	P1	F3	179.56(7)	C6	N4	C10	113.29(12)
F4	P1	F2	90.73(6)	C7	N4	Cu1	99.35(9)
F4	P1	F5	89.89(6)	C10	N4	Cu1	115.21(9)
F3	P1	F1	90.16(6)	C10	N4	C7	112.16(12)
F3	P1	F6	89.53(6)	N1	C8	C7	109.63(12)
F3	P1	F5	89.67(6)	O1	C12	C11	116.10(13)
F2	P1	F1	90.31(6)	O2	C12	O1	124.74(15)
F2	P1	F6	90.24(6)	O2	C12	C11	119.12(14)
F2	P1	F3	89.72(6)	N1	C11	C12	109.89(12)
F2	P1	F5	179.38(7)	N4	C6	C5	108.49(12)
F5	P1	F6	89.70(6)	N4	C7	C8	108.63(12)
C12	O1	Cu1	112.28(10)	N2	C9	C10	110.74(12)
C13	N3	Cu1	112.19(10)	N1	C1	C2	112.54(12)
C13	N3	C4	110.53(12)	N3	C4	C3	113.58(12)
C13	N3	C5	109.34(12)	N2	C3	C4	111.56(13)

Table S12. Bond Angles for $[CuL_1]PF_6$.

Atom	Atom	Atom	Angle/˚	Atom	Atom	Atom	Angle/˚
C4	N3	Cu1	105.02(9)	N3	C5	C6	111.24(12)
C4	N3	C5	114.06(12)	N2	C2	C1	112.82(12)
C5	N3	Cu1	105.59(9)	N4	C10	C9	112.67(12)

Table S13. Torsion Angles for $[CuL_1]PF_6$.

A	B	C	D	Angle/˚	A	B	C	D	Angle/˚
Cu1	O1	C12	O2	-174.22(13)	C8	N1	C1	C2	68.06(16)
Cu1	O1	C12	C11	3.60(16)	C11	N1	C8	C7	95.52(14)
Cu1	N3	C4	C3	6.12(15)	C11	N1	C1	C2	-161.23(12)
Cu1	N3	C5	C6	18.01(15)	C6	N4	C7	C8	-156.45(13)
Cu1	N2	C9	C10	-33.31(15)	C6	N4	C10	C9	94.51(15)
Cu1	N2	C3	C4	-54.17(12)	C7	N4	C6	C5	158.55(12)
Cu1	N2	C2	C1	-10.03(14)	C7	N4	C10	C9	-130.37(13)
Cu1	N1	C8	C7	-20.30(14)	C9	N2	C3	C4	59.79(17)
Cu1	N1	C11	C12	-34.32(13)	C9	N2	C2	C1	-130.02(14)
Cu1	N1	C1	C2	-49.66(13)	C1	N1	C8	C7	-135.25(13)
Cu1	N4	C6	C5	53.53(12)	C1	N1	C11	C12	76.54(15)
Cu1	N4	C7	C8	-52.02(12)	C13	N3	C4	C3	127.32(14)
Cu1	N4	C10	C9	-17.69(16)	C13	N3	C5	C6	-102.88(15)
O1	C12	C11	N1	21.70(18)	C4	N3	C5	C6	132.80(13)
O2	C12	C11	N1	-160.36(14)	C3	N2	C9	C10	-136.59(14)
N3	C4	C3	N2	37.89(18)	C3	N2	C2	C1	91.70(16)
N2	C9	C10	N4	33.93(18)	C5	N3	C4	C3	-109.00(15)
N1	C8	C7	N4	50.97(16)	C2	N2	C9	C10	83.24(16)
N1	C1	C2	N2	40.50(17)	C2	N2	C3	C4	-162.43(13)
N4	C6	C5	N3	-51.18(17)	C10	N4	C6	C5	-68.61(15)
C8	N1	C11	C12	-152.31(12)	C10	N4	C7	C8	70.21(15)

Table S14.Hydrogen Atom Coordinates (Å×10^4) and Isotropic Displacement Parameters (Å^2×10^3) for [CuL$_1$]PF$_6$.

Atom	*x*	*y*	*z*	U(eq)
H8A	-515.46	6260.49	1827.4	16
H8B	1141.74	7055.02	1158.22	16
H11A	369.85	3567.16	3083.68	17
H11B	-611.87	4910.56	3590.11	17
H6A	545.06	9489.43	3920.99	19
H6B	1133.73	10972.13	2992.6	19
H7A	-420.27	9055.42	1783.5	18
H7B	-720.83	8018.68	2973.85	18
H9A	5049.34	8986.4	835.03	21
H9B	5100.88	9580.55	1911.58	21
H1A	2579.84	4466.71	1513.15	17
H1B	3398.05	4137.53	2580.62	17
H13A	2180.03	7975.31	5553.24	30
H13B	4009.64	8450.74	5609.42	30
H13C	3622.91	6698.61	5552.05	30
H4A	6003.61	7467.34	4354.36	21
H4B	5974.3	9004.83	3433.75	21
H3A	6838.8	7156.06	2481.66	21
H3B	5670.27	5841.64	3253.52	21
H5A	3884.09	10463.17	3194.22	19
H5B	2910.69	10430.96	4361.23	19
H2A	5318.32	5599.99	1369.82	19
H2B	3976.44	6730.14	821.26	19
H10A	2727.49	10698.04	1444.37	20
H10B	2305.19	9283.52	933.78	20

Table S15. Crystal data and structure refinement for $[CoL_2Cl]PF_6$.

Identification code	th117bp21c
Empirical formula	$C_{15}H_{29}ClCoF_6N_4O_2P$
Structural formula	$C_{15}H_{29}N_4O_2CoCl$, PF_6
Formula weight	536.77
Temperature/K	120
Crystal system	monoclinic
Space group	$P2_1/c$
a/Å	8.6543(8)
b/Å	12.5675(12)
c/Å	18.5750(18)
α/°	90
β/°	92.778(2)
γ/°	90
Volume/Å^3	2017.9(3)
Z	4
ρ_{calc}g/cm^3	1.767
μ/mm^{-1}	1.138
F(000)	1104.0
Crystal size/mm^3	0.232 × 0.09 × 0.056
Radiation	MoKα (λ = 0.71073Å)
2Θ range for data collection/°	3.914 to 54.454
Index ranges	-11 ≤ h ≤ 11, -15 ≤ k ≤ 16, -23 ≤ l ≤ 23
Reflections collected	41924
Independent reflections	4491 [R_{int} = 0.0507, R_{sigma} = 0.0317]
Data/restraints/parameters	4491/0/272
Goodness-of-fit on F^2	1.097
Final R indexes [I>=2σ (I)]	R_1 = 0.0681, wR_2 = 0.1693
Final R indexes [all data]	R_1 = 0.0883, wR_2 = 0.1793
Largest diff. peak/hole / e Å^{-3}	1.82/-1.43

Table S16. Fractional Atomic Coordinates (×10^4) and Equivalent Isotropic Displacement Parameters (Å^2×10^3) for $[CoL_2Cl]PF_6$. U_{eq} is defined as 1/3 of the trace of the orthogonalised U_{IJ} tensor.

Atom	*x*	*y*	*z*	U(eq)
Co1	7118.7(6)	2076.4(5)	6578.9(3)	25.12(19)
P1	8196.2(15)	6994.2(10)	5960.5(6)	30.8(3)
F2	8757(4)	6123(2)	6541.6(15)	40.6(7)

Table S16. Fractional Atomic Coordinates (×10[4]) and Equivalent Isotropic Displacement Parameters (Å[2]×10[3]) for $[CoL_2Cl]PF_6$. U_{eq} is defined as 1/3 of the trace of the orthogonalised U_{IJ} tensor.

Atom	*x*	*y*	*z*	U(eq)
F3	6493(3)	6514(3)	5955.5(15)	39.7(7)
F4	7792(4)	7787(2)	6594.4(15)	41.3(7)
F1	8596(4)	6200(3)	5324.1(16)	48.1(8)
O1	5154(3)	1401(3)	6480.8(17)	27.6(7)
F6	9905(4)	7468(3)	5963.9(19)	57.3(10)
F5	7628(5)	7854(3)	5374.4(17)	53.2(9)
O2	4000(4)	-37(3)	6035(2)	40.4(9)
N2	6499(5)	3006(3)	5738(2)	28.0(8)
N3	6309(5)	3114(3)	7313(2)	32.6(9)
N1	7845(5)	1034(4)	5867(2)	35.1(10)
N4	9104(5)	2830(4)	6665(2)	43.3(12)
C14	5141(6)	526(4)	6139(2)	31.0(10)
C2	6458(6)	2280(4)	5095(2)	35.7(11)
C1	7801(6)	1550(5)	5146(3)	37.6(12)
C4	4625(6)	4166(4)	6408(3)	41.1(12)
C11	7689(6)	3857(4)	5679(3)	38.5(12)
C3	4917(6)	3488(4)	5753(3)	35.1(11)
C13	6667(6)	158(4)	5863(3)	38.0(12)
C15	6105(7)	2600(5)	8033(3)	42.6(13)
C5	4771(6)	3574(4)	7114(3)	37.4(12)
C6	7511(6)	3957(5)	7408(3)	47.2(15)
C7	9057(6)	3432(6)	7356(3)	51.1(16)
C12	9239(6)	3578(5)	6034(3)	43.1(14)
C10	9409(6)	551(6)	6011(3)	51.9(16)
C9	10685(6)	1356(7)	6112(4)	64(2)
C8	10524(6)	2113(6)	6726(3)	58.5(19)
Cl1	7797.0(17)	808.2(14)	7430.4(8)	51.9(4)

Table S17. Anisotropic Displacement Parameters (Å[2]×10[3]) for $[CoL_2Cl]PF_6$. The Anisotropic displacement factor exponent takes the form: $-2\pi^2[h^2a^{*2}U_{11}+2hka^*b^*U_{12}+\ldots]$.

Atom	U_{11}	U_{22}	U_{33}	U_{23}	U_{13}	U_{12}
Co1	17.8(3)	42.2(4)	15.5(3)	0.7(3)	2.9(2)	0.0(3)
P1	38.4(7)	32.2(7)	22.1(6)	4.0(5)	4.4(5)	-5.0(5)
F2	46.4(17)	41.9(18)	33.5(15)	10.0(13)	2.2(13)	3.9(14)
F3	36.5(15)	47.9(18)	34.9(16)	-1.0(14)	2.6(12)	-6.5(14)
F4	59.6(19)	35.8(17)	28.5(15)	-3.1(12)	1.2(13)	-0.6(14)

Table S17. Anisotropic Displacement Parameters (Å^2×10^3) for [CoL_2Cl]PF_6. The Anisotropic displacement factor exponent takes the form: $-2\pi^2[h^2a^{*2}U_{11}+2hka^*b^*U_{12}+\ldots]$.

Atom	U_{11}	U_{22}	U_{33}	U_{23}	U_{13}	U_{12}
F1	68(2)	46.6(19)	30.8(16)	-5.1(14)	20.3(15)	-1.5(16)
O1	25.1(15)	29.0(17)	28.8(16)	-0.9(13)	2.9(12)	1.9(13)
F6	50(2)	75(2)	48(2)	4.0(18)	10.3(16)	-26.7(19)
F5	88(3)	39.1(18)	31.5(16)	11.1(14)	-11.0(16)	-9.4(18)
O2	47(2)	32(2)	42(2)	1.9(16)	6.8(16)	-7.2(17)
N2	33(2)	29(2)	21.8(18)	0.1(15)	-2.8(15)	-3.9(17)
N3	29(2)	41(2)	28(2)	-7.4(17)	8.0(16)	-10.1(17)
N1	34(2)	45(3)	27(2)	8.0(18)	11.1(17)	11.5(19)
N4	21.5(19)	84(4)	25(2)	3(2)	3.5(16)	-11(2)
C14	36(2)	30(3)	26(2)	9(2)	1.4(19)	0(2)
C2	51(3)	40(3)	16(2)	3.2(19)	-2(2)	0(2)
C1	46(3)	46(3)	22(2)	0(2)	12(2)	-2(2)
C4	41(3)	31(3)	52(3)	-1(2)	5(2)	0(2)
C11	47(3)	38(3)	31(3)	2(2)	5(2)	-14(2)
C3	33(2)	32(3)	40(3)	6(2)	-8(2)	4(2)
C13	49(3)	32(3)	34(3)	4(2)	11(2)	7(2)
C15	46(3)	58(4)	24(2)	-8(2)	11(2)	-6(3)
C5	32(2)	41(3)	40(3)	-11(2)	10(2)	-1(2)
C6	48(3)	64(4)	31(3)	-17(3)	9(2)	-27(3)
C7	36(3)	87(5)	31(3)	-12(3)	0(2)	-28(3)
C12	33(3)	66(4)	32(3)	-6(3)	11(2)	-17(3)
C10	38(3)	74(4)	46(3)	13(3)	15(2)	25(3)
C9	26(3)	115(6)	52(4)	15(4)	15(2)	19(3)
C8	21(2)	108(6)	46(3)	13(4)	-2(2)	-4(3)
Cl1	44.7(7)	67.3(10)	43.5(8)	4.6(7)	1.5(6)	4.2(7)

Table S18. Bond Lengths for [CoL_2Cl]PF_6

Atom	Atom	Length/Å	Atom	Atom	Length/Å
Co1	O1	1.901(3)	N3	C15	1.504(6)
Co1	N2	2.002(4)	N3	C5	1.482(7)
Co1	N3	2.035(4)	N3	C6	1.489(6)
Co1	N1	1.986(4)	N1	C1	1.487(6)
Co1	N4	1.961(4)	N1	C13	1.500(7)
Co1	Cl1	2.3014(17)	N1	C10	1.495(6)
P1	F2	1.597(3)	N4	C7	1.492(7)
P1	F3	1.592(3)	N4	C12	1.512(7)

Table S18. Bond Lengths for [CoL$_2$Cl]PF$_6$

Atom	Atom	Length/Å	Atom	Atom	Length/Å
P1	F4	1.594(3)	N4	C8	1.524(8)
P1	F1	1.598(3)	C14	C13	1.512(7)
P1	F6	1.594(3)	C2	C1	1.480(8)
P1	F5	1.595(3)	C4	C3	1.517(7)
O1	C14	1.270(6)	C4	C5	1.508(8)
O2	C14	1.222(6)	C11	C12	1.508(8)
N2	C2	1.502(6)	C6	C7	1.499(9)
N2	C11	1.492(6)	C10	C9	1.503(10)
N2	C3	1.498(6)	C9	C8	1.496(10)

Table S19. Bond Angles for [CoL$_2$Cl]PF$_6$.

Atom	Atom	Atom	Angle/˚	Atom	Atom	Atom	Angle/˚
O1	Co1	N2	88.92(15)	C3	N2	C2	105.9(4)
O1	Co1	N3	90.85(15)	C15	N3	Co1	112.3(3)
O1	Co1	N1	87.26(16)	C5	N3	Co1	114.7(3)
O1	Co1	N4	177.55(18)	C5	N3	C15	104.2(4)
O1	Co1	Cl1	87.36(11)	C5	N3	C6	111.5(4)
N2	Co1	N3	93.48(17)	C6	N3	Co1	105.9(3)
N2	Co1	Cl1	171.78(12)	C6	N3	C15	108.2(4)
N3	Co1	Cl1	93.90(13)	C1	N1	Co1	108.5(3)
N1	Co1	N2	86.96(16)	C1	N1	C13	109.1(4)
N1	Co1	N3	178.04(17)	C1	N1	C10	108.8(4)
N1	Co1	Cl1	85.55(12)	C13	N1	Co1	104.5(3)
N4	Co1	N2	89.00(18)	C10	N1	Co1	117.5(4)
N4	Co1	N3	87.97(18)	C10	N1	C13	108.2(4)
N4	Co1	N1	93.94(19)	C7	N4	Co1	104.8(3)
N4	Co1	Cl1	94.87(15)	C7	N4	C12	110.9(5)
F2	P1	F1	90.12(18)	C7	N4	C8	106.9(4)
F3	P1	F2	89.68(17)	C12	N4	Co1	109.7(3)
F3	P1	F4	90.31(17)	C12	N4	C8	109.4(4)
F3	P1	F1	89.56(18)	C8	N4	Co1	114.9(4)
F3	P1	F6	179.6(2)	O1	C14	C13	116.6(4)
F3	P1	F5	89.92(19)	O2	C14	O1	124.6(5)
F4	P1	F2	89.99(17)	O2	C14	C13	118.8(5)
F4	P1	F1	179.8(2)	C1	C2	N2	109.8(4)
F4	P1	F6	90.0(2)	C2	C1	N1	108.3(4)
F4	P1	F5	90.53(18)	C5	C4	C3	114.2(4)

Table S19. Bond Angles for [CoL_2Cl]PF_6.

Atom	Atom	Atom	Angle/˚	Atom	Atom	Atom	Angle/˚
F6	P1	F2	90.12(19)	N2	C11	C12	113.8(4)
F6	P1	F1	90.1(2)	N2	C3	C4	115.4(4)
F6	P1	F5	90.3(2)	N1	C13	C14	112.3(4)
F5	P1	F2	179.35(19)	N3	C5	C4	116.5(4)
F5	P1	F1	89.36(19)	N3	C6	C7	107.4(5)
C14	O1	Co1	114.9(3)	N4	C7	C6	109.9(4)
C2	N2	Co1	105.1(3)	C11	C12	N4	112.5(4)
C11	N2	Co1	108.4(3)	N1	C10	C9	113.7(5)
C11	N2	C2	111.4(4)	C8	C9	C10	115.3(5)
C11	N2	C3	110.3(4)	C9	C8	N4	115.0(5)
C3	N2	Co1	115.6(3)				

Table S20.Torsion Angles for [CoL_2Cl]PF_6.

A	B	C	D	Angle/˚	A	B	C	D	Angle/˚
Co1	O1	C14	O2	178.2(4)	C1	N1	C10	C9	68.0(6)
Co1	O1	C14	C13	-0.2(5)	C11	N2	C2	C1	75.7(5)
Co1	N2	C2	C1	-41.6(4)	C11	N2	C3	C4	-65.7(5)
Co1	N2	C11	C12	22.1(5)	C3	N2	C2	C1	-164.5(4)
Co1	N2	C3	C4	57.8(5)	C3	N2	C11	C12	149.6(4)
Co1	N3	C5	C4	-57.7(5)	C3	C4	C5	N3	61.9(6)
Co1	N3	C6	C7	-35.8(5)	C13	N1	C1	C2	78.5(5)
Co1	N1	C1	C2	-34.8(5)	C13	N1	C10	C9	-173.6(5)
Co1	N1	C13	C14	21.7(5)	C15	N3	C5	C4	179.1(4)
Co1	N1	C10	C9	-55.7(6)	C15	N3	C6	C7	84.9(5)
Co1	N4	C7	C6	-44.4(6)	C5	N3	C6	C7	-161.1(4)
Co1	N4	C12	C11	21.2(6)	C5	C4	C3	N2	-61.1(6)
Co1	N4	C8	C9	58.1(6)	C6	N3	C5	C4	62.6(5)
O1	C14	C13	N1	-15.6(6)	C7	N4	C12	C11	-94.1(5)
O2	C14	C13	N1	165.9(4)	C7	N4	C8	C9	174.0(5)
N2	C2	C1	N1	51.5(6)	C12	N4	C7	C6	74.0(6)
N2	C11	C12	N4	-29.1(6)	C12	N4	C8	C9	-65.8(7)
N3	C6	C7	N4	54.8(6)	C10	N1	C1	C2	-163.7(5)
N1	C10	C9	C8	60.3(7)	C10	N1	C13	C14	147.6(4)
C2	N2	C11	C12	-93.1(5)	C10	C9	C8	N4	-62.5(8)
C2	N2	C3	C4	173.7(4)	C8	N4	C7	C6	-166.7(5)
C1	N1	C13	C14	-94.2(5)	C8	N4	C12	C11	148.1(5)

Table S21. Hydrogen Atom Coordinates (Å×10^4^) and Isotropic Displacement Parameters (Å^2^×10^3^) for $[CoL_2Cl]PF_6$.

Atom	*x*	*y*	*z*	U(eq)
H2A	5486.15	1863.31	5075.85	43
H2B	6487.34	2706.82	4648.17	43
H1A	8768.94	1951.23	5083.19	45
H1B	7699.06	1003.08	4762.42	45
H4A	5367.8	4765.86	6425.2	49
H4B	3571.01	4470.73	6351.47	49
H11A	7304.71	4516.32	5899.64	46
H11B	7826.16	4004.2	5161.99	46
H3A	4144.72	2906.14	5726.58	42
H3B	4742.83	3931.52	5315.95	42
H13A	7072.52	-435.82	6166.46	46
H13B	6495.22	-112.31	5364.5	46
H15A	5360.3	2014.47	7977.04	64
H15B	5718.81	3127.93	8368.42	64
H15C	7101.17	2322.27	8222.7	64
H5A	4489.6	4066.05	7502.45	45
H5B	4003.1	2989.04	7097.53	45
H6A	7440.04	4303.66	7883.68	57
H6B	7363.24	4505.72	7028.44	57
H7A	9882.56	3978.01	7377.2	61
H7B	9242.68	2940.11	7767.22	61
H12A	9761.05	4239.72	6203.81	52
H12B	9891.68	3242.99	5674.13	52
H10A	9642.3	78.74	5604.04	62
H10B	9384.66	105.42	6450.29	62
H9A	11678.03	971.52	6186.25	77
H9B	10738.48	1774.06	5662.29	77
H8A	11458.87	2567.17	6765.71	70
H8B	10488.94	1696.19	7177.53	70

Table S22. Crystal data and structure refinement for $[CuL_2]PF_6$.

Identification code	TH19b - twin4a
Empirical formula	$C_{15}H_{29}CuF_6N_4O_2P$
Formula weight	505.93
Structural formula	$C_{15}H_{29}N_4O_2Cu$, PF_6
Temperature/K	127
Crystal system	monoclinic
Space group	$P2_1/c$
a/Å	9.5256(3)
b/Å	8.0586(3)
c/Å	25.2288(8)
α/°	90
β/°	99.776(2)
γ/°	90
Volume/Å^3	1908.52(11)
Z	4
ρ_{calc}g/cm^3	1.761
μ/mm^{-1}	3.145
F(000)	1044.0
Crystal size/mm^3	0.222 × 0.156 × 0.008
Radiation	CuKα (λ = 1.54178 Å)
2Θ range for data collection/°	7.11 to 133.158
Index ranges	-11 ≤ h ≤ 11, 0 ≤ k ≤ 9, 0 ≤ l ≤ 29
Reflections collected	3322
Independent reflections	3322 [R_{int} = 0.0552, R_{sigma} = 0.0424]
Data/restraints/parameters	3322/0/264
Goodness-of-fit on F^2	1.070
Final R indexes [I>=2σ (I)]	R_1 = 0.0417, wR_2 = 0.1008
Final R indexes [all data]	R_1 = 0.0465, wR_2 = 0.1034
Largest diff. peak/hole / e Å^{-3}	0.52/-0.38

Table S23.Fractional Atomic Coordinates (×10^4) and Equivalent Isotropic Displacement Parameters (Å^2×10^3) for $[CuL_2]PF_6$. U_{eq} is defined as 1/3 of the trace of the orthogonalised U_{IJ} tensor.

Atom	*x*	*y*	*z*	U(eq)
Cu1	8415.5(6)	1017.5(6)	6581.1(2)	19.13(15)
P1	2888.7(10)	5796.8(10)	5798.6(3)	21.5(2)
F6	2883(3)	7678(2)	5579.2(9)	31.1(5)

Table S23.Fractional Atomic Coordinates (×10^4) and Equivalent Isotropic Displacement Parameters (Å^2×10^3) for [CuL_2]PF_6. U_{eq} is defined as 1/3 of the trace of the orthogonalised U_{IJ} tensor.

Atom	*x*	*y*	*z*	U(eq)
F5	1863(3)	6342(3)	6207.5(10)	34.8(5)
F3	2911(3)	3926(3)	6020.7(10)	35.9(5)
F2	1530(3)	5385(3)	5354.7(10)	35.3(6)
F4	4249(3)	6209(3)	6248.3(10)	33.7(5)
F1	3915(3)	5269(3)	5391.2(10)	38.6(6)
O1	9086(3)	-1055(3)	6926.1(10)	26.3(6)
O2	9113(4)	-2500(3)	7674.9(11)	35.0(7)
N1	8015(4)	3351(3)	6294.2(12)	21.5(6)
N2	9661(4)	688(4)	6002.5(12)	21.6(6)
N3	6430(4)	290(4)	6036.7(13)	24.6(7)
N4	7154(4)	1237(4)	7163.4(13)	26.0(7)
C1	9189(4)	3680(5)	5985.0(15)	25.1(8)
C5	6608(5)	-1196(5)	5703.1(16)	27.7(8)
C2	9428(4)	2186(4)	5648.7(15)	22.3(7)
C10	8115(5)	4649(4)	6725.8(15)	27.6(8)
C14	8681(5)	-1346(4)	7370.2(14)	26.6(8)
C15	11184(4)	590(5)	6254.6(15)	26.3(8)
C4	7804(5)	-1083(4)	5377.1(15)	28.3(8)
C6	5481(5)	-123(5)	6422.0(16)	32.7(9)
C12	5989(4)	1768(5)	5701.2(15)	27.8(8)
C7	5637(4)	1106(5)	6888.5(16)	30.0(8)
C3	9305(4)	-887(5)	5692.4(15)	25.1(8)
C11	6584(4)	3424(5)	5947.4(16)	27.5(8)
C13	7554(6)	-199(5)	7536.2(17)	35.6(10)
C8	7408(5)	2806(5)	7480.9(16)	29.9(9)
C9	7150(5)	4390(5)	7144.4(16)	30.5(9)

Table S24. Anisotropic Displacement Parameters (Å^2×10^3) for [CuL_2]PF_6. The Anisotropic displacement factor exponent takes the form: $-2\pi^2[h^2a^{*2}U_{11}+2hka^*b^*U_{12}+\dots]$.

Atom	U_{11}	U_{22}	U_{33}	U_{23}	U_{13}	U_{12}
Cu1	23.1(3)	16.1(2)	18.1(2)	1.70(17)	3.1(2)	1.1(2)
P1	23.6(4)	17.4(4)	23.2(4)	2.3(3)	3.4(4)	0.8(4)
F6	35.8(13)	20.9(10)	35.8(11)	10.0(8)	3.9(11)	0.5(10)
F5	36.9(13)	38.2(13)	31.8(12)	6.0(9)	13.3(11)	7.8(11)
F3	45.5(14)	19.1(10)	42.1(13)	7.6(9)	4.5(12)	0.3(12)
F2	33.4(13)	36.4(13)	32.3(13)	0.1(10)	-5.5(11)	-6.8(11)

Table S24. Anisotropic Displacement Parameters (Å^2×10^3) for [CuL$_2$]PF$_6$. The Anisotropic displacement factor exponent takes the form: $-2\pi^2[h^2a^{*2}U_{11}+2hka^*b^*U_{12}+\ldots]$.

Atom	U_{11}	U_{22}	U_{33}	U_{23}	U_{13}	U_{12}
F4	33.2(12)	29.2(12)	34.6(12)	0.2(9)	-6.2(10)	1.0(11)
F1	39.1(14)	42.5(14)	37.5(13)	1.1(11)	15.7(11)	11.6(12)
O1	35.4(15)	18.3(12)	24.7(12)	3.5(9)	3.6(11)	1.3(11)
O2	54(2)	25.5(13)	25.3(13)	6.1(10)	4.9(14)	0.4(14)
N1	28.1(17)	14.7(13)	21.2(13)	-1.1(10)	3.2(13)	1.1(13)
N2	26.4(15)	18.8(15)	19.8(14)	0.1(11)	4.5(12)	5.2(13)
N3	24.4(16)	26.4(16)	22.4(15)	-3.5(12)	2.5(12)	-4.2(13)
N4	31.1(17)	22.3(16)	25.3(16)	-0.9(12)	7.1(13)	-1.3(15)
C1	27.8(19)	21.6(18)	26.6(18)	2.2(14)	6.3(16)	-1.6(15)
C5	32(2)	22.5(18)	27.8(19)	-5.9(14)	2.2(16)	-6.4(17)
C2	27.1(19)	19.5(17)	20.2(16)	3.3(13)	3.9(15)	1.5(15)
C10	37(2)	16.9(16)	28.5(17)	-5.0(13)	5.1(17)	-1.5(17)
C14	35(2)	20.1(16)	22.8(16)	-0.2(13)	0.6(16)	-7.4(17)
C15	24.3(18)	27.5(19)	26.6(18)	1.8(14)	2.9(15)	6.6(15)
C4	37(2)	20.8(17)	25.4(17)	-4.4(13)	1.4(16)	-3.0(19)
C6	30(2)	36(2)	32.9(19)	-9.5(16)	8.4(17)	-10.2(18)
C12	22.5(19)	33(2)	25.7(18)	-5.0(14)	-3.3(15)	2.3(16)
C7	24(2)	33(2)	34(2)	-5.5(16)	7.6(16)	-3.4(18)
C3	32(2)	18.1(17)	25.7(18)	-2.9(13)	6.1(16)	3.0(16)
C11	27(2)	25(2)	29(2)	4.8(14)	1.6(17)	7.6(16)
C13	49(3)	33(2)	27.6(19)	7.9(16)	16.2(19)	4(2)
C8	35(2)	32(2)	23.1(17)	-6.4(15)	6.9(17)	-0.6(18)
C9	39(2)	22.6(19)	29.7(19)	-5.7(14)	5.7(17)	1.4(18)

Table S25. Bond Lengths for [CuL$_2$]PF$_6$.

Atom	Atom	Length/Å	Atom	Atom	Length/Å
Cu1	O1	1.941(3)	N2	C15	1.483(5)
Cu1	N1	2.028(3)	N2	C3	1.500(5)
Cu1	N2	2.049(3)	N3	C5	1.490(5)
Cu1	N3	2.219(3)	N3	C6	1.473(5)
Cu1	N4	2.057(3)	N3	C12	1.480(5)
P1	F6	1.613(2)	N4	C7	1.497(5)
P1	F5	1.598(3)	N4	C13	1.499(5)
P1	F3	1.607(2)	N4	C8	1.494(5)
P1	F2	1.596(3)	C1	C2	1.512(5)
P1	F4	1.605(2)	C5	C4	1.517(6)

Table S25. Bond Lengths for [CuL_2]PF_6.

Atom	Atom	Length/Å	Atom	Atom	Length/Å
P1	F1	1.592(3)	C10	C9	1.527(6)
O1	C14	1.267(5)	C14	C13	1.528(6)
O2	C14	1.232(5)	C4	C3	1.522(6)
N1	C1	1.492(5)	C6	C7	1.526(5)
N1	C10	1.501(4)	C12	C11	1.539(6)
N1	C11	1.490(5)	C8	C9	1.529(6)
N2	C2	1.496(4)			

Table S26. Bond Angles for [CuL_2]PF_6.

Atom	Atom	Atom	Angle/˚	Atom	Atom	Atom	Angle/˚
O1	Cu1	N1	170.34(13)	C2	N2	C3	112.0(3)
O1	Cu1	N2	91.23(12)	C15	N2	Cu1	110.1(2)
O1	Cu1	N3	103.38(12)	C15	N2	C2	109.5(3)
O1	Cu1	N4	86.65(12)	C15	N2	C3	107.3(3)
N1	Cu1	N2	87.86(13)	C3	N2	Cu1	112.0(2)
N1	Cu1	N3	86.27(13)	C5	N3	Cu1	113.3(2)
N1	Cu1	N4	94.46(13)	C6	N3	Cu1	101.8(2)
N2	Cu1	N3	93.48(12)	C6	N3	C5	109.4(3)
N2	Cu1	N4	177.48(12)	C6	N3	C12	114.4(3)
N4	Cu1	N3	85.69(13)	C12	N3	Cu1	105.7(2)
F5	P1	F6	89.77(13)	C12	N3	C5	111.9(3)
F5	P1	F3	90.35(14)	C7	N4	Cu1	107.3(2)
F5	P1	F4	89.71(13)	C7	N4	C13	111.0(3)
F3	P1	F6	179.41(16)	C13	N4	Cu1	105.3(3)
F2	P1	F6	89.97(13)	C8	N4	Cu1	113.2(3)
F2	P1	F5	89.91(15)	C8	N4	C7	111.3(3)
F2	P1	F3	90.61(13)	C8	N4	C13	108.4(3)
F2	P1	F4	179.59(15)	N1	C1	C2	110.5(3)
F4	P1	F6	90.18(13)	N3	C5	C4	115.2(3)
F4	P1	F3	89.24(13)	N2	C2	C1	109.2(3)
F1	P1	F6	89.80(14)	N1	C10	C9	115.8(3)
F1	P1	F5	179.52(16)	O1	C14	C13	117.6(3)
F1	P1	F3	90.07(14)	O2	C14	O1	124.9(4)
F1	P1	F2	90.30(14)	O2	C14	C13	117.5(4)
F1	P1	F4	90.07(15)	C5	C4	C3	116.6(3)
C14	O1	Cu1	115.4(2)	N3	C6	C7	111.8(3)
C1	N1	Cu1	103.8(2)	N3	C12	C11	114.7(3)

Table S26. Bond Angles for $[CuL_2]PF_6$.

Atom	Atom	Atom	Angle/˚	Atom	Atom	Atom	Angle/˚
C1	N1	C10	107.1(3)	N4	C7	C6	111.3(3)
C10	N1	Cu1	113.7(2)	N2	C3	C4	118.0(3)
C11	N1	Cu1	110.1(2)	N1	C11	C12	116.1(3)
C11	N1	C1	112.2(3)	N4	C13	C14	114.2(3)
C11	N1	C10	109.7(3)	N4	C8	C9	114.4(3)
C2	N2	Cu1	106.0(2)	C10	C9	C8	116.0(4)

Table S27. Torsion Angles for $[CuL_2]PF_6$.

A	B	C	D	Angle/˚	A	B	C	D	Angle/˚
Cu1	O1	C14	O2	-172.0(3)	C1	N1	C11	C12	92.3(4)
Cu1	O1	C14	C13	9.1(5)	C5	N3	C6	C7	161.1(3)
Cu1	N1	C1	C2	43.6(3)	C5	N3	C12	C11	-146.1(3)
Cu1	N1	C10	C9	-57.0(4)	C5	C4	C3	N2	-66.2(4)
Cu1	N1	C11	C12	-22.8(4)	C2	N2	C3	C4	-57.9(4)
Cu1	N2	C2	C1	33.8(3)	C10	N1	C1	C2	164.2(3)
Cu1	N2	C3	C4	61.0(4)	C10	N1	C11	C12	-148.7(3)
Cu1	N3	C5	C4	-54.0(4)	C15	N2	C2	C1	-84.9(4)
Cu1	N3	C6	C7	41.0(4)	C15	N2	C3	C4	-178.1(3)
Cu1	N3	C12	C11	-22.4(4)	C6	N3	C5	C4	-166.9(3)
Cu1	N4	C7	C6	35.7(4)	C6	N3	C12	C11	88.8(4)
Cu1	N4	C13	C14	-1.5(4)	C12	N3	C5	C4	65.3(4)
Cu1	N4	C8	C9	59.2(4)	C12	N3	C6	C7	-72.5(4)
O1	C14	C13	N4	-4.8(6)	C7	N4	C13	C14	114.3(4)
O2	C14	C13	N4	176.2(4)	C7	N4	C8	C9	-61.9(5)
N1	C1	C2	N2	-53.9(4)	C3	N2	C2	C1	156.2(3)
N1	C10	C9	C8	61.6(5)	C11	N1	C1	C2	-75.3(4)
N3	C5	C4	C3	61.8(4)	C11	N1	C10	C9	66.9(4)
N3	C6	C7	N4	-54.9(5)	C13	N4	C7	C6	-79.0(4)
N3	C12	C11	N1	31.7(5)	C13	N4	C8	C9	175.7(4)
N4	C8	C9	C10	-62.8(5)	C8	N4	C7	C6	160.1(3)
C1	N1	C10	C9	-171.1(3)	C8	N4	C13	C14	-123.0(4)

Table S28. Hydrogen Atom Coordinates (Å×10^4) and Isotropic Displacement Parameters (Å^2×10^3) for [CuL$_2$]PF$_6$.

Atom	*x*	*y*	*z*	U(eq)
H1A	10076.48	3937.81	6237.49	30
H1B	8943.51	4653.54	5748.08	30
H5A	5703.09	-1390.86	5453.18	33
H5B	6781.26	-2173.16	5943.23	33
H2A	8588.66	2013.53	5363.93	27
H2B	10269.84	2373.25	5473.88	27
H10A	7885.84	5739.27	6551.84	33
H10B	9113.88	4697.89	6914.74	33
H15A	11456.72	1610.06	6457.81	40
H15B	11771.91	461.15	5974.09	40
H15C	11327.86	-364.22	6498.4	40
H4A	7606.55	-128.63	5128.46	34
H4B	7779.91	-2096.85	5154.28	34
H6A	5705.97	-1253.83	6565.06	39
H6B	4481.32	-121.1	6233.16	39
H12A	4935.27	1829.14	5634.28	33
H12B	6299.45	1622.48	5349.06	33
H7A	5295.77	2211.2	6751.59	36
H7B	5037.77	740.41	7151.23	36
H3A	9484.73	-1824.12	5948.77	30
H3B	9979.46	-1003.29	5436.33	30
H11A	6633.34	4213.01	5650.42	33
H11B	5899.33	3881.03	6163.83	33
H13A	6687.49	-857.15	7556.35	43
H13B	7914.41	236.71	7900.86	43
H8A	8404.62	2810.16	7673.37	36
H8B	6777.43	2818.09	7754.86	36
H9A	6149.08	4385.37	6956.73	37
H9B	7265.24	5352.22	7391.83	37
